# Serotonin transporter (*SERT*) polymorphisms, personality and problem-solving in urban great tits

**DOI:** 10.1038/s41598-021-03466-7

**Published:** 2021-12-20

**Authors:** Andrea S. Grunst, Melissa L. Grunst, Nicky Staes, Bert Thys, Rianne Pinxten, Marcel Eens

**Affiliations:** 1 Department of Biology, Behavioural Ecology and Ecophysiology Group, University of Antwerp, Antwerp, Belgium; 2 Littoral Environnement Et Sociétés, La Rochelle Université, La Rochelle, France; 3 Centre for Research and Conservation, Royal Zoological Society of Antwerp, Antwerp, Belgium; 4 Faculty of Social Sciences, Antwerp School of Education, University of Antwerp, Antwerp, Belgium

**Keywords:** Behavioural genetics, Evolutionary ecology

## Abstract

Understanding underlying genetic variation can elucidate how diversity in behavioral phenotypes evolves and is maintained. Genes in the serotonergic signaling pathway, including the serotonin transporter gene (*SERT*), are candidates for affecting animal personality, cognition and fitness. In a model species, the great tit (*Parus major*), we reevaluated previous findings suggesting relationships between *SERT* polymorphisms, neophobia, exploratory behavior and fitness parameters, and performed a first test of the relationship between single nucleotide polymorphisms (SNPs) in SERT and problem-solving in birds. We found some evidence for associations between *SERT* SNPs and neophobia, exploratory behavior and laying date. Furthermore, several SNPs were associated with behavioral patterns and success rates during obstacle removal problem-solving tests performed at nest boxes. In females, minor allele homozygotes (AA) for nonsynonymous SNP226 in exon 1 made fewer incorrect attempts and were more likely to problem-solve. In both sexes, there was some evidence that minor allele homozygotes (CC) for SNP84 in exon 9 were more likely to problem-solve. Only one SNP-behavior relationship was statistically significant after correcting for multiple comparisons, but several were associated with substantial effect sizes. Our study provides a foundation for future research on the genetic basis of behavioral and cognitive variation in wild animal populations.

## Introduction

Animal personality is defined by consistent, among individual variation in behavioral traits such as neophobia, exploratory behavior and aggressiveness. Among individual variation in behavior was once discounted as noise around an adaptive mean. However, behavioral ecologists now recognize that personality variation descripts an integral part of the variation in behavioral phenotypes, and is shaped by selective pressures^1–6^. Personality variation reflects alternative behavioral strategies that involve differential balance of life-history tradeoffs, and affects how individuals respond to ecological and social challenges, with ultimate fitness implications^1–6^. For example, individuals that are less neophobic, bolder and more exploratory are often more successful in invading new habitats, but can also be susceptible to risks arising from novel predators and poisoning^7,8^. In addition, personality variation may be related to variation in cognitive strategies and innovative problem-solving performance, wherein individuals solve novel problems, independent of overall cognitive ability^9^. For instance, less neophobic and more exploratory individuals may learn more rapidly and perform better on innovative problem-solving tasks, because they are more likely to attempt novel tasks and interact with novel environmental features^9–11^. Thus, cognitive performance traits and personality may be interrelated, and might be affected by shared physiological and genetic mechanisms.

Personality and cognitive traits are often heritable and show genetic variance. Quantitative genetic studies present estimates as high as 50% for the amount of personality variation attributed to genetics^12–14^. Quantitative genetics and artificial selection studies also suggest that cognitive traits, including general cognitive ability and cognitive performance within specific domains, can show substantial genetic variation^15^. However, other studies have yielded low estimates for the heritability of cognitive performance traits, including innovative problem-solving^16^. Developmental, environmental and genetic factors combine to determine behavioral and cognitive traits, and multiple, interacting genes affect these complex phenotypes, with most having small effects^14,15^. The extent to which genetics underlie personality and cognitive variation is therefore challenging to elucidate, and specific genes underlying this variation are challenging to identify. For instance, personality traits, including boldness and aggressiveness^17–20^, and cognitive traits including problem-solving performance^21^, often differ between urban and rural bird populations. However, whether these differences are genetic in origin, and which genes might be involved, remains debated^22,23^. Nonetheless, there are a number of exciting cases in which specific genes underlying personality and cognitive variation have been identified, via approaches including candidate gene and genome wide association studies. For example, candidate gene studies in species ranging from humans^24–26^ to wild great tits^27–29^ have linked polymorphisms in the dopamine receptor D4 (*DRD4*) gene to personality variation in novelty seeking and exploratory behavior. Moreover, genes linked to variation in cognitive traits have also been identified in some cases. To give a few examples, catechol-*O*-methyltransferase (*COMT*) gene variants influence insight problem-solving in humans^30^, and polymorphism in the arginine vasopressin V1a receptor gene (*AVPR1A*) affects social cognition in chimpanzees^31^.

Though more limited in scope than genome wide association studies, candidate gene studies allow researchers to access specific hypotheses regarding relationships between behavioral traits and genes of interest^13,32^. Genes related to the dopaminergic and serotonergic neurotransmitter systems are good candidates for affecting variation in personality and cognitive performance patterns^23–29,33–41^. Serotonin is a monoamine hormone that has multifaceted physiological, behavioral and cognitive effects in both vertebrates and invertebrates. Serotonin acts as a critical neurotransmitter, but is also prevalent outside of the central nervous system and plays central roles in a diversity of physiological functions, including cardiovascular function and gut motility. Thus, effects of serotonin function on behavioral patterns may conceivably arise through modulation of physiological patterns, as well as via important effects on the central nervous system^42^. Effects of serotonin function on behavior and cognition have been most extensively studied in humans, wherein serotonin levels are inversely related to anxiety, impulsiveness and aggressiveness^43,44^. Serotonin dysfunction in nonhuman primates and model laboratory rodents has also been linked to similar behavioral traits^45,46^, and shown to interfere with cognitive processes^46–48^. The serotonin transporter gene (*SERT*), which was the focus of our study, is a member of the neurotransmitter sodium symporter family. The serotonin transporter (SERT) protein selectively cotransports serotonin (5-HT) out of the synaptic cleft and into nerve cells along with one Na^+^ and one Cl^-^ ion, while one K^+^ is transported in the opposite direction. Uptake of extracellular serotonin (5-HT) by SERT terminates the action of serotonin at receptor sites after its release, and thus serves as a key regulator of serotonergic signaling^49^. *SERT* polymorphism in humans^50–53^, nonhuman primates^54–56^ and model rodents^57^ has been implicated in an array of behavioral and cognition-related traits, especially sensitivity to stress, anxiety and social cognition. Variation in SERT function may thus affect both personality and cognitive traits in animals via changes in serotonergic signaling.

Avian studies that suggest a role for *SERT* polymorphisms in mediating genetic differences in behavior are growing in scope. Some avian studies have found differences in *SERT* polymorphisms between urban and rural bird populations^37^. Thus, *SERT* polymorphisms that differ between urban and rural populations could underlie some of the aforementioned behavioral differences between the two^17–22^, including differences in neophobia^18^, exploratory behavior^19^, and innovativeness^21^. A few other avian studies have linked *SERT* polymorphisms to individual differences in personality traits. In great tits (*Parus major*), *SERT* SNPs were related to neophobia during parental care^40^ and hissing behavior during nest defense^41^. Research that replicates and expands upon such studies is needed to elucidate whether *SERT* polymorphism is implicated in personality variation across multiple great tit populations, and to what extent the same polymorphisms are involved. Our research team recently failed to replicate the previous finding that *SERT* SNP187 in exon 1 is related to hissing behavior in great tits^41^, but found some association between two other *SERT* SNPs and hissing behavior (SNP66 in exon 13 and SNP144 in exon 12)^58^. In the same study, we found no associations between female-female aggression and *SERT* SNPs, even when taking age-related plasticity in aggression into account^58^. Thus, different *SERT* polymorphisms might be responsible for behavioral variation across different populations and behavioral contexts.

Avian studies regarding the genetics of problem-solving are few in number, and have not yet investigated effects of *SERT* polymorphisms on cognitive traits. This is despite a growing body of evidence linking serotonin and cognitive processes in other animal taxa^46,53,56^, although note that documented relationships between serotonin and cognitive processes are often indirect effects mediated via individual differences in stress-sensitivity^48,53^. In addition, although differences in problem-solving performance have been documented between urban and rural bird populations^21^, potential genetic underpinnings of these differences remain largely unidentified. However, a few previous avian studies in free-living populations have investigated the genetic underpinnings of problem-solving performance. For example, expression patterns of the *N*-methyl-D-aspartate (NMDA) glutamate receptor were linked to differences in innovative problem-solving between two species of closely related finches^59^. On the other hand, a study in a pedigreed population of great tits suggested little heritability of problem-solving performance^16^.

Using great tits as a study species, we aimed to expand upon past avian studies involving relationships between *SERT* polymorphisms and personality traits (boldness, neophobia and exploratory behavior), and perform a first test of relationships between *SERT* polymorphisms and problem-solving in birds. We previously found that birds occupying more disturbed territories, closer to roads and paths, are less neophobic and more likely to problem solve^60,61^. We thus specifically aimed to determine whether *SERT* polymorphisms could provide a genetic basis for differences in neophobia and problem-solving between birds on more and less disturbed territories. We assessed associations between SNPs in the promoter region and 13 exons of the great tit *SERT* gene and behaviors measured during three standardized tests: (1) boldness and novel object (neophobia) tests (2) novel environment exploration tests, and (3) obstacle removal problem-solving tests. Neophobia and exploratory behavior show moderate repeatability in our study population (*r* ≥ 0.30), and thus constitute putative personality traits^60–64^. We also evaluated whether *SERT* SNPs were related to reproductive fitness (nestlings fledged, laying date), as suggested by a previous study in great tits^41^. Understanding genetic underpinnings of fitness traits may grant insight into how behavioral variation is maintained in populations.

## Materials and methods

### Study site

We researched great tits from 5 study sites: Umicore (UM; 51°09′58.28″N, 4°21′23.76″E), Fort 7 (F7; 51°09′52.55″N, 4°22′40.46″E), Fort 6 (F6/Campus Drie Eiken), Fort 5 (F5; 51°10′02.90″N, 4°26′00.26″E) and Fort 4 (F4; 51°10′23.80″N, 4°27′38.46″E) located in the greater metropolis area of Antwerp, Belgium. Only novel environment exploration testing was performed at F6, to avoid conflicts with on-going studies. Study populations are not genetically isolated, with regular instances of dispersal occurring between sites. Most adults at all sites are color banded and females are equipped with passive integrated transponder (PIT) tags.

Study sites are located at varying distances from the Umicore smelter and metal refinery facility, a metal pollution point source, in Hoboken (south of Antwerp city center). In previous studies, we found no effect of proximity to Umicore on neophobia or problem-solving^60,61^, but found a relationship between metal exposure and exploratory behavior^63,64^. We do not consider gene-by-environment interactions mediated by metal exposure^65,66^ due to lack of individual metal exposure measurements and because testing for genotype-by-site interactions would require a larger sample size. Rather, we include site as a random effect in statistical models to account for potential site-level differences in behavior. Within study sites, territories differ in anthropogenic disturbance levels, with territories closer to site edges being closer to roads, paths and buildings^60,61,67^. As a proxy for disturbance level, we measured the distance of nest boxes from the nearest road or path using Google Earth measurement tools^60,61,67^.

### Behavioral tests

We tested 93 genotyped, incubating females on novel object tests^60^, 156 genotyped birds (92 females, 62 males) on novel environment exploration tests^62–64^, and 93 genotyped birds (52 females, 41 males) for problem-solving^61^. Novel object tests consisted of two trial types, in random order, zero to three days apart (2018 and 2019, March to April). During novel object trials, we flushed females from the nest, placed a novel object on the nest box, and video-recorded for 1-h to determine the female’s latency to return and enter^60^. Baseline trials followed the same protocol, but with no novel object. We determined the female’s boldness following disturbance at the nest from baseline trials, and additionally determined neophobia from novel object trials. However, note that return latencies during novel object trials are also affected by boldness as measured during baseline trials. Return latencies are individually repeatable across novel object and baseline trials, and across repeated novel object trials, suggesting repeatability of both boldness and neophobia^60^.

Novel environment exploration tests occurred during winter (2017 to 2019, December to early March). Birds sleeping in nest boxes were captured at night, transported to the University of Antwerp’s Campus Drie Eiken, and housed overnight in individual cages with food and water. The next morning (0800–1100), we performed tests using standard protocol^68^. Birds were individually released into a novel environment room containing 5 artificial trees via sliding doors that connect cages to the room. We counted hops and flights made within 2-min, and calculated exploration scores as the total number of movements. After all individuals were tested, birds were weighed, measured, aged, sexed and banded (if not already banded), and released at capture sites before noon. Novel environment exploration behavior is repeatable in our populations^62–64,68^.

Obstacle removal tests occurred in 2019 at UM, F7 and F4 during the nestling stage (nestlings 8–12 days old, April 26–May 22, 0930–1630)^61^. We first placed a barrier (a modified nest box trap) on top of the nest box secured it in an open position, and video-recorded nestling provisioning for 1-h. This period allowed parent birds to habituate to the barrier, to minimize effects of neophobia on problem-solving. After one hour, we returned to the nest box and lowered the barrier. To create a mechanism whereby birds could lift the barrier, a string was looped around the metal hinge at the top of the trap and a wooden popsicle stick attached to the string. Birds could raise the barrier by landing and pulling on the stick, while lifting the barrier with their heads. We video-recorded nest boxes for 1.25 h. with the barrier lowered, and determined latency to contact the apparatus, time on the nest box, incorrect attempts directed at the barrier (pecks at the barrier) and number of entries. Note that birds that entered the nest box multiple times during the problem-solving test mastered the problem-solving task faster, or more thoroughly, than those that entered only once. Thus, number of entries grants additional information about individual problem-solving performance. Latency to contact the barrier might be influenced by both the motivation of birds to enter the nest box, and neophobia. However, effects of neophobia are expected to be minimized, because we habituated birds to the test apparatus before lowering the barrier^61^. Obstacle removal tests were mostly performed with the barrier elevated 1.5 cm from the nest box face, to facilitate entry. A subset of 31 pairs (for 28 pairs at least one pair member was successful on the first test) was tested a second time with the barrier flat against the nest box^61^. Trials for which an individual was never observed by the nest box were dropped from analyses, and nests with similar brood sizes were selected whenever possible. Testing protocols are described in detail in previous publications^60–64^.

### Fitness-related breeding parameters

During 2018 and 2019, we monitored reproduction via nest box checks every other day (late March through May). We recorded the date that the first egg was laid, clutch size, brood size, and fledging success. We analyzed relationships between SNPs and female laying date, and number of nestlings fledged for both sexes, because these parameters were the most variable and least redundant.

### Genotyping

To identify SNPs, we first amplified the great tit *SERT* promoter region and thirteen exons using PCR. Most primers were as in Timm et al. (2018)^41^, but those for exon 1 and 12 were redesigned using Geneious Prime 2020.0.4 (Table S1). Thermocycling conditions and reaction mixes have been previously described^58^. Direct sequencing of PCR amplicons was performed at the University of Antwerp’s genomics core facility using a sanger sequencing platform. We used Geneious Prime 2020.0.4 to align sequences to the great tit reference genome (parus_major1.1:ENSPMJG00000019668, Ensembl genome browser) and identified SNPs via manual inspection. We initially genotyped between 46 and 48 individuals representing a wide range in behavioral phenotypes across all loci to determine the presence and frequencies of SNP. We then genotyped SNPs exceeding 5% minor allele frequency in the rest of the population (*N* = 181). SNPs with minor allele frequencies of < 10% in the whole population were excluded from analyses. SNPs for which there were < 4 individuals with the minor allele (minor allele homozygotes or heterozygotes) in behavioral datasets were also excluded from analyses due to model convergence problems. SNPs that deviated from Hardy–Weinberg equilibrium (assessed via Chi-square tests with a Holm’s correction, Table 1) were additionally excluded.

**Table 1:** Twenty-six SNPs in the *SERT* gene and associated amino acid (AA) changes for exonic SNPs found in great tits in Antwerp, Belgium.

Locus	Coordinate	Location	M/m	mm	Mm	MM	%m	X_1_^2^	P	Protein coding	AA change
SNP030	chr19:5979530	Promoter	c/t	8	30	145	12.57	11.81	0.001		
**SNP031**	chr19:5979529	Promoter	a/t	9	61	114	21.47	0.051	0.820		
**SNP100**	chr19:5979460	Promoter	a/g	4	32	147	10.93	1.898	0.168		
SNP115	chr19:5979445	Promoter	c/a	34	54	98	32.80	21.67	< 0.001		
**SNP250**	chr19:5979310	Promoter	g/a	12	54	125	20.42	3.230	0.072		
SNP288	chr19:5979272	Promoter	g/t	15	28	148	15.18	35.45	< 0.001		
SNP415	chr19:5979145	Promoter	c/t	3	37	150	11.32	0.168	0.682		
**SNP438**	chr19:5979122	Promoter	c/t	12	73	106	25.39	0.014	0.904		
SNP592	chr19:5978968	Promoter	t/c	16	46	129	20.42	12.80	< 0.001		
SNP663	chr19:5978897	Promoter	g/a	1	44	144	12.17	1.498	0.221		
**SNP163**	chr19:5978840	Exon 1	g/a	4	47	145	14.03	0.007	0.933	Synonymous	
**SNP187**	chr19:5978816	Exon 1	c/t	4	58	134	16.84	0.630	0.427	Synonymous	
**SNP226**	chr19:5978777	Exon 1	t/a	20	72	104	28.57	1.96	0.162	Non-synonymous	E26D
SNP32	chr19:5977679	Exon 2	c/a	0	19	176	4.87	0.511	0.475	Synonymous	
SNP100	chr19:5976873	Exon 3	c/t	2	28	168	8.08	0.457	0.499	Non-synonymous	A231V
SNP101	chr19:5976872	Exon 3	g/a	0	17	181	4.29	0.398	0.528	Synonymous	
SNP125	chr19:5976812	Exon 3	g/a	3	32	163	9.60	0.929	0.335	Synonymous	
**SNP170**	chr19:5976794	Exon 3	c/g	6	33	159	11.36	5.902	0.015	Synonymous	
SNP187	chr19:5976777	Exon 3	t/a	2	21	175	6.31	2.116	0.146	Non-synonymous	L260Q
SNP51	chr19:5975781	Exon 5	c/t	0	16	178	4.12	0.358	0.549	Synonymous	
**SNP36**	chr19:5973968	Exon 6	t/c	50	91	57	48.23	1.256	0.262	Synonymous	
SNP48	chr19:5971868	Exon 9	g/a	0	23	175	5.81	0.752	0.386	Synonymous	
**SNP51**	chr19:5971865	Exon 9	c/t	10	49	139	17.42	3.881	0.049	Synonymous	
**SNP84**	chr19:5971832	Exon 9	t/c	41	100	57	45.96	0.055	0.814	Synonymous	
SNP144	chr19:5968682	Exon 12	c/t	4	21	172	7.36	9.390	0.002	Synonymous	
**SNP66**	chr19:5967914	Exon 13	c/t	14	64	101	25.70	0.727	0.394	Synonymous	

Loci are named according to nucleotide positions within the promoter region or exons. The coordinate column gives the position within the great tit genome on chromosome 19. M = major allele, m = minor allele, %m = minor allele frequency. Chi-square tests were used to assess the null hypothesis of Hardy–Weinberg (HW) equilibrium. *P* values in red italics indicate significant deviations from HW (Holm’s corrected *P*_critical_ = 0.002). Sample sizes differ between loci due to sequencing failures. SNPs considered in statistical analyses are shown in bold text.

Linkage disequilibrium was assessed with the web-based application SNPStats, which uses matrices of genotype data to calculate haplotypes, linkage disequilibrium statistics (*D, D'*, Pearson’s *r*) and *P *values based on Chi-square tests^69^. For exonic SNPs, we also determined whether SNPs were synonymous (not changing the amino acid sequence) or nonsynonymous (changing the sequence). Sample sizes varied slightly across loci as not all SNPs were successfully sequenced for all individuals.

To infer putative functional consequences of coding variants, we used SNAP2 to predict effects of variants on protein function^70^. SNAP2 is a trained classifier based on a machine-learning device called neural network, which distinguishes between nonsynonymous SNPs that cause functional effects and neutral variants. The effect of a variant is believed to be important to native protein function and structure if the SNAP2 score exceeds 50; neutral if the score is below − 50; and not possible to reliably determine when the score is between 50 and − 50^70^. SNAP2 results were verified using a different function prediction tool, PROVEAN^71^. In addition, we also used the web-based tool Protter^72^ to visualize the location of nonsynonymous SNPs within SERT.

### Statistical analyses

We performed statistical analyses in R 3.6.1^73^. We first used linear (LMMs; for modeling log-transformed return latencies) and generalized linear mixed models (GLMMs; binomial for problem-solving success, Poisson for incorrect attempts)^74,75^ to investigate associations among behavioral and cognitive traits. Specifically, we tested the relationship between neophobia and boldness (log-transformed return latencies, dependent variable), and the first measured exploration score of females (predictor). Relationships between traits measured during problem-solving tests (dependent) and the putative personality traits were tested using results from first tests of personality traits as predictor variables. Relationships among traits measured during problem-solving tests were also tested. For all models, individual and study site were entered as random effects, and trial number as a covariate. When testing the above relationships, neophobia was adjusted for boldness by extracting residuals of an LMM predicting return latencies during novel object trials from return latencies during baseline trials. We also report the relationship between return latencies during novel object and baseline trials.

We assessed the repeatability of putative personality traits (neophobia, boldness, exploratory behavior) in the current dataset using R package rptR. Package rptR estimates repeatability based on variance components extracted from mixed models^76^. Models used to estimate repeatability were linear mixed models (LMMs)^75^ for boldness and neophobia, and a Poisson GLMM^74^ for exploratory behavior. Repeatability estimates were adjusted for effects of year, test date and test number for exploratory behavior, and year and test number for boldness and neophobia. For neophobia, we assessed repeatability both when adjusting for baseline return latencies (see above), and when not making this adjustment.

To model effects of SNPs on behaviors, problem-solving and breeding parameters, we constructed general generic models, wherein genotypes were entered as three level factors. If results from general generic models suggested dominance (e.g. CT = TT ≠ CC) or overdominance (heterozygote ≠ homozygotes), we reran models combining genotypes. We analyzed the relationship between each SNP (in separate models to avoid collinearity and over complexity) and six behaviors: (1) female latency to return to the nest during novel object tests, wherein latency during baseline trials measured boldness and latency during novel object tests measured neophobia, (2) exploratory behavior, (3) problem-solving success, (4) number of entries during obstacle removal tests (results only reported where significant relationships were found with problem-solving success), (5) incorrect attempts on the barrier, and (6) latency to contact the obstacle removal test apparatus. For behavioral patterns during obstacle removal tests (problem-solving success, number of entries, incorrect attempts and latency to contact), we performed separate models for males and females, because random effects were overparameterized with both sexes in models. For males, a low number of problem-solving individuals in our dataset made testing associations with problem-solving success difficult for some SNPs (18 genotyped females, but only 9 genotyped males were successful). We also constructed models predicting two fitness-related parameters: female laying date (log-transformed) and number of fledglings in both sexes.

We had repeated measures for many individuals, and thus adopted a mixed modeling approach^74^. Return latencies (log-transformed) during novel object tests, latency to contact during obstacle removal tests, and laying date (log-transformed) were analyzed using linear mixed effects models (LMMs) with Satterthwaite approximations for degrees of freedom (R package lmerTest)^75^. Success on obstacle removal tests was analyzed using a generalized linear mixed effects model (GLMM) with a binomial distribution. Exploratory behavior, nest box entries, incorrect attempts on the barrier, and fledgling number were analyzed using Poisson GLMMs. For most models, we used individual identity and study site as random effects, and added year as a random when predicting behaviors measured across multiple years (neophobia, exploratory behavior). However, for problem-solving success in males, we used only study site as a random effect, because attempting to include an individual-level random effect led to convergence problems. Thus, only results from first problem-solving tests on individual males were included in analyses. Binomial and Poisson GLMMs included an observation level random effect to control for overdispersion, where necessary^77^.

Some models contained additional interactions and covariates. Models predicting return latencies during novel object tests included two-way interactions between trial type (baseline, novel object) and genotype, and trial type and distance to the path/road (we previously found this interaction to be significant)^60^. If neophobia were affected by SNPs, we expected statistically significant relationships only within novel object trials, whereas if boldness were affected by SNPs, we expected that relationships would be present within baseline trials or across both trial types. Trial number (first versus second test on a bird) was included as a covariate, whereas clutch size, trial date and female age were not considered, as these variables were nonsignificant in a previous study^60^. Models for exploratory behavior included an interaction between sex and genotype, test date and trial number. Models for problem-solving success, nest box entries, incorrect attempts on the barrier, and latency to contact the barrier contained trial number and distance to paths/roads (square-root transformed). Brood size, nestling age, time and date were not included in these models, because these variables were found to be unrelated to problem-solving performance in a previous study^61^. However, to better understand potential behavioral mechanisms underlying relationships between SNPs and problem-solving success, we repeated models that included significant SNP-problem-solving associations with incorrect attempts on the barrier and latency to contact the test apparatus as additional predictor variables. Models involving incorrect attempts on the barrier as the dependent variable additionally used time on the nest box (log-transformed) as an offset variable. Lay date was initially included in models involving fledgling numbers, but was statistically nonsignificant, so was removed.

We performed posthoc tests for comparisons among genotypes using R package emmeans (Tukey method)^78^, and applied Bonferroni corrections to account for the number of SNPs (*N* = 12; significance threshold α = 0.004). Although Bonferroni corrections are stringent, applying a different method, such as false discovery rate^79^, would not qualitatively change conclusions. To obtain a measure of effect size for SNPs that showed substantial associations with behavior, we used marginal R^2^ (*R*^2^*m*), calculated using the method of Nakagawa et al. (2013)^80^ (function r2 in R package performance)^81^. *R*^2^*m* reflects the proportion of variance explained by fixed effects in a mixed effects model. We also report conditional *R*^2^ (*R*^2^*c*), which reflects the proportion of variance explained by random plus fixed effects. Models were reduced using a stepwise process (α = 0.05) involving removing nonsignificant interaction terms first, and then nonsignificant main effects when significant predictors were present. Model diagnostics were performed using R package DHARMa^82^. Sample sizes varied among the behavioral tests and among SNPs due to sequencing failures. We thus indicate sample sizes for all models in the results section.

### Ethical statement

Animal experiments were approved by the University of Antwerp’s ethical committee (ID 2016-71), and conducted in accordance with Belgian and Flemish laws. Methodology adhered to the ASAB/ABS guidelines for use of animals in behavioral research, and we made all possible efforts to minimize the stress experienced by birds during handling and behavioral testing. The Belgian Royal Institute for Natural Sciences (Koninklijk Belgisch Instituut voor Natuurwetenschappen; KBIN) provided banding licenses for authors and technical personnel.

## Results

### Relationships among traits and repeatability of behaviors

Novel object neophobia (adjusted for baseline return latency) was related to exploration behavior (*β* = −0.016 ± 0.007, *t*_78_ = −2.092, *P* = 0.039), but did not predict problem-solving success (*β* = 0.296 ± 0.303, *Z* = 0.978, *P* = 0.328), incorrect attempts (*β* = −0.288 ± 0.975, *Z* = −295, *P* = 0.768), or latency to contact the test apparatus (*β* = 0.075 ± 0.141, t_66_ = 0.532, *P* = 0.596). Problem solving success (*β* = −0.255 ± 0.321, Z = −0.794, *P* = 0.427), incorrect attempts (*β* = 0.119 ± 0.482, Z = 0.247, *P* = 0.805), and latency to contact the test apparatus (*β* = −0.867 ± 2.43, t_87_ = −0.356, *P* = 0.722) were also not predicted by exploratory behavior. Return latency during novel object trials (unadjusted for baseline behavior) was related to return latency during baseline trials (boldness) (*β* = 0.018 ± 0.004, *t*_146_ = 4.281, *P* < 0.001), but boldness was not significantly related to exploratory behavior (*β* = −0.001 ± 0.009, *t*_75_ = −0.113, *P* = 0.91) or traits measured during problem-solving tests (*P* > 0.30 in all cases). Problem-solving success was negatively associated with incorrect attempts (*β* = −0.905 ± 0.279, Z = −2.121, *P* = 0.033) and latency to contact the test apparatus (*β* = −0.960 ± 0.427, Z = −3.430, *P* < 0.001).

Exploratory behavior (*r* ± SE [95% CI] = 0.581 ± 0.041 [0.319, 0.481], *P* < 0.001) and novel object neophobia either adjusted (*r* ± SE [95% CI] = 0.432 ± 0.103 [0.221, 0.609], *P* < 0.001) or unadjusted (*r* ± SE [95% CI] = 0.473 ± 0.091 [0.290, 0.635], *P* < 0.001) for boldness showed statistically significant repeatability. Boldness was also significantly repeatable (*r* ± SE [95% CI] = 0.436 ± 0.176 [0.052, 0.758], *P* = 0.047).

### Genetic polymorphisms

Of 26 SNPs identified at a minor allele frequency of > 5% in our initial sample, 5 deviated from Hardy–Weinberg equilibrium, and 9 displayed a minor allele frequency of < 10% in the entire sample or too few individuals (< 4) with the minor allele in behavioral datasets, resulting in 12 SNPs retained for analysis (Table 1). Four retained SNPs were in the promoter region. Of the 8 exonic SNPs retained for analysis, only SNP226 in exon 1 was nonsynonymous, causing the amino acid change E26D (glutamic acid replaced by aspartic acid). Two other nonsynonymous SNPs located in exon 3 (SNP100, A231V and SNP187, L260Q) were present at < 10% minor allele frequency. None of the SNPs had been previously reported in great tits, although Timm et al. (2018, 2019)^40,41^ identified a different nucleotide change at one of the positions (SNP187 in exon 1; A/T in previous study, C/T in current study). There was no complete linkage disequilibrium, but substantial linkage disequilibrium existed between some loci within and between the promoter region and exon 1 (Supplementary Table S2).

SNAP2 analysis could not reliably predict the functional significance of the amino acid change caused by SNP226 in exon 1 (E26D: strength: − 42, reliability: 72%), the only nonsynonymous SNP retained in our analyses. For the other two amino acid changes identified (Table 1), SNAP2 analysis revealed a likely neutral effect for A231V caused by SNP100 in exon 3 (strength: − 70, reliability: 82%), and failed to reliably predict functionality for the change L260Q associated with SNP187 in exon 3 (strength: − 19, reliability: 57%). Visualization of the location of nonsynonymous SNPs using Protter demonstrated that E26D (SNP226 in exon 1) is located within the N terminus of SERT, whereas A231V (SNP100 exon 3) and L260Q (SNP187 exon 3) are located within extracellular loop 2 (Supplementary Fig. S1).

### Relationships between SNPs and behaviors

#### Novel object neophobia, boldness and exploratory behavior

**Figure 1:**
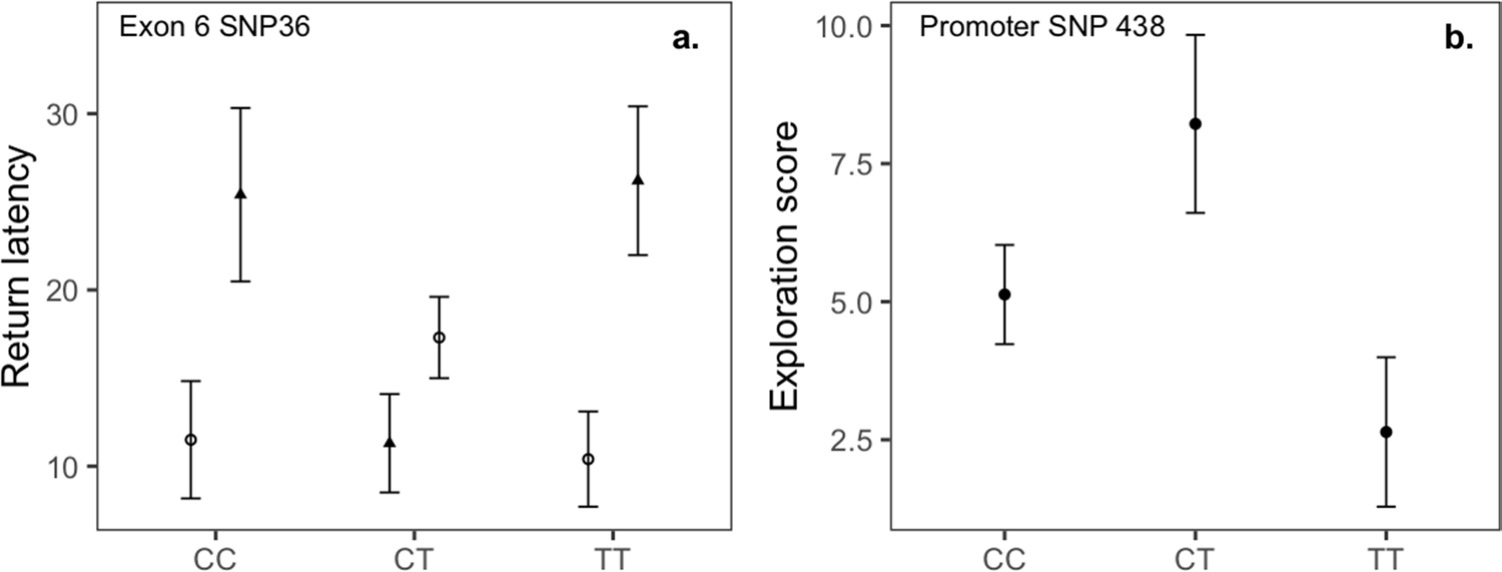
Relationships between *SERT* SNPs, neophobia/boldness and exploratory behavior. Mean values and standard errors (error bars) for groups were extracted using R package emmeans and back transformed from the log scale; (**a**) exon 6 SNP36 and return latency (minutes) during novel object tests. Triangles indicate baseline trials (boldness) and circles novel object trials (neophobia). *N* (tests, females) = baseline: CC: 24, 20; CT: 48, 41; TT: 37, 29; novel object: CC: 31, 21; CT: 74, 43; TT: 50, 28. (**b**) Promoter region SNP438 and exploratory behavior (movements/2 min). *N* (tests, birds) = CC: 133, 87; CT: 81:51; TT: 12, 9.

Genotype at SNP36 in exon 6 and trial type interacted to predict return latency during novel object tests (*F*_2,173_ = 3.570, *P* = 0.030). Females heterozygous for SNP36 (CT) returned to the nest box faster during novel object trials than homozygous birds, suggesting an effect on neophobia (genotype CC or TT; *β* = 0.402 ± 0.166, *t*_104_ = 2.418, *P* = 0.017; *R*^2^*m* = 0.048, *R*^2^*c* = 0.49), whereas heterozygotes and homozygotes did not differ in return latencies during baseline trials, suggesting no effect on boldness following disturbance at the nest (*β* = −0.049 ± 0.177, *t*_83_ = −0.276, *P* = 0.783) (Fig. 1a). However, the interaction was nonsignificant after accounting for multiple comparisons, as was also the case for all other SNP-behavior relationships examined, with one exception, noted below.

Promoter region SNP438 was associated with exploratory behavior (χ_2_^2^ = 6.830, *P* = 0.033), with heterozygotes (CT) tending to have higher exploration scores than homozygotes (*β* = −0.592 ± 0.246, *Z* = −2.405, *P* = 0.016; *R*^2^*m* = 0.035, *R*^2^*c* = 0.463) (Fig. 1b). We found no other relationships between promoter region or exonic SNPs and either neophobia/boldness (Table S3, S4) or exploratory behavior (Table S5, S6). Including distance to paths/roads in models did not alter conclusions.

#### Problem-solving performance

##### Incorrect attempts and latency to contact

**Figure 2:**
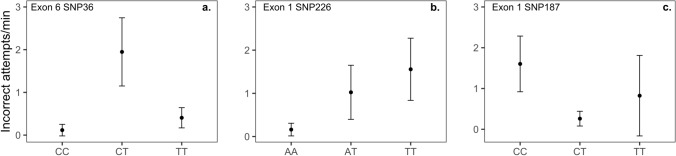
Relationships between *SERT* SNPs and incorrect attempts directed at the barrier during obstacle removal tests. Mean values and standard errors (error bars) for groups were extracted using R package emmeans and back transformed from the log scale. Values are incorrect attempts per minute on the nest box; (**a**) Exon 6 SNP36, *N* (tests, females) = CC: 12, 9; CT: 39, 28; TT: 22, 15. (**b**) Exon 1 SNP226, *N* = AA: 13, 9; AT: 20, 16; TT: 40, 27, (**c**) exon 1 SNP187, *N* = CC: 47, 34; CT: 21, 15; TT: 5, 3.

In females, but not males, there was a relationship between SNP36 in exon 6 and incorrect attempts during obstacle removal tests (Table 2a; Fig. 2a). As was also the case for novel object neophobia (see above), heterozygous females (CT) differed from homozygotes, with heterozygotes pecking at the barrier more (Table 2a; Fig. 2a). The overall relationship between SNP36 and incorrect attempts on the barrier was significant at the Bonferroni threshold level, although this was not the case for pairwise comparisons (Table 2a).

**Table 2:** General generic and dominance effect GLMMs for relationships between exonic *SERT* SNPs and incorrect attempts on the barrier by female great tits during obstacle removal problem-solving tests.

(a) SNP36 exon 6					
General generic model		*R*^2^*m* = 0.300, 0.181^a^		*R*^2^*c* = 0.381	
Fixed effects	*β* ± SE	*Z*	*P*	*χ*_2_^2^	*P*
Intercept	0.697 ± 1.235	0.565	0.572		
SNP36 CC	− 2.811 ± 1.128	2.491	0.034^b^		
SNP36 TT	− 1.570 ± 0.608	− 2.581	0.026^b^	11.34	0.003
Trial number	− 1.895 ± 0.641	− 2.954	0.003		

Random effects	Variance	SD	N		
Observation	2.754	1.659	73		
Individual	0.510	0.714	52		
Study site	< 0.001	0.002	3		

(b) SNP226 exon 1					
General generic model		*R*^2^*m* = 0.214, 0.145^a^		*R*^2^*c* = 0.416	
Fixed Effects	*β* ± SE	*Z*	*P*	*χ*_2_^2^	*P*
Intercept	0.494 ± 1.175	0.421	0.6739		
SNP226 AT	1.849 ± 1.002	1.845	0.155^c^		
SNP226 TT	2.268 ± 0.952	2.382	0.045^c^	6.335	0.042
Trial number	− 1.549 ± 0.612	− 2.53	0.011		

Random effects	Variance	SD	N		
Observation	2.385	1.544	73		
Individual	1.218	1.104	52		
Study site	< 0.001	< 0.001	3		

Dominance effect model		*R*^2^*m* = 0.212, 0.141^a^		*R*^2^*c* = 0.397	
Fixed effects	*β* ± SE	*Z*	*P*		
Intercept	0.510 ± 1.182	0.432	0.665		
SNP226 AT/TT	2.114 ± 0.913	2.315	0.020		
Trial number	− 1.555 ± 0.620	− 2.508	0.012		

Random effects	Variance	SD	N		
Observation	2.510	1.580	73		
Individual	1.110	1.060	52		
Study site	< 0.001	< 0.001	3		

(c) SNP187 exon 1					
General generic model		*R*^2^*m* = 0.210, 0.126^a^		*R*^2^*c* = 0.378	
Fixed effects	*β* ± SE	*Z*	*P*	*χ*_2_^2^	*P*
Intercept	2.959 ± 0.799	3.703	< 0.001		
SNP187 TC	− 1.814 ± 0.700	− 2.59	0.026^d^		
SNP187 TT	− 0.665 ± 1.235	− 0.538	0.852^d^	7.130	0.028
Trial number	− 1.660 ± 0.625	− 2.656	0.007		

Random effects	Variance	SD	N		
Observation	2.543	1.595	73		
Individual	0.993	0.996	52		
Study site	< 0.001	< 0.001	3		

Time on the nest box was entered as an offset variable. (a) SNP36 exon 6, (b) SNP226 exon 1, (c) SNP187 exon 1.

^a^Marginal R^2^ for all fixed effects, and the SNP alone.

^b^*P* values for post-hoc Tukey tests with CT as reference, *P* = 0.534 for the CC–TT contrast.

^c^*P* values for post-hoc Tukey tests, *P* = 0.843 for the AT–TT contrast.

^d^*P* values from posthoc Tukey tests, *P* = 0.657 for the TT–TC contrast.

*N* = 73 observations, 52 females; exon 6 SNP36: 9 CC, 28 CT, 15 TT (a); exon 1 SNP226*:* 9 AA, 16 AT, 27 (b) TT; exon 1 SNP187: 33 CC, 16 CT, 3 TT (c).

Nonsynonymous SNP226 (Table 2b; Fig. 2b) and synonymous SNP187 in exon 1 (Table 2c; Fig. 2c) were also associated with incorrect attempts by females. Females with rare genotype AA at SNP226 pecked the barrier less than females with AT or TT, although the *P* value for the AA–AT contrast was > 0.05 after posthoc correction (Table 2b; Fig. 2b). Females with genotype CC at SNP187 pecked the barrier more than birds with CT. Behavior did not differ between females with genotype TT at SNP187 and those with the other genotypes (Table 2c; Fig. 2c), but few (three) females exhibited genotype TT, making these comparisons difficult.

No other promoter region or exonic SNP was related to incorrect attempts on the barrier in either females (Table S7, S8) or males (Table S9, S10). We also found no statistically significant relationships between any of the SNPs and latency to contact the test apparatus in either females (S11, S12) or males (S13, S14).

##### Problem-solving success

**Figure 3:**
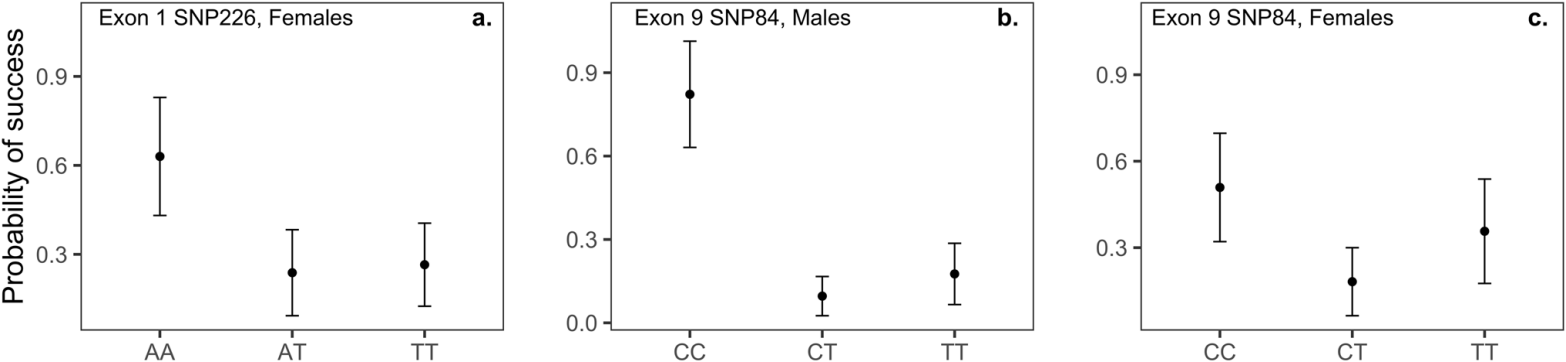
Relationships between *SERT* SNPs and problem-solving success. Mean values and standard errors (error bars) for groups were extracted using R package emmeans and back transformed from the logit scale; (**a**) exon 1 SNP226 in females, *N* (test, females) = AA: 13, 9; AT: 20, 16; TT: 40, 27, (**b**) exon 9 SNP84 in males, *N* (males) = CC: 5; CT: 20; TT: 16, (**c**) exon 9 SNP84 in females. *N* (tests, females) = CC: 19, 11; CT: 37, 28; TT: 17, 13.

Nonsynonymous SNP226 in exon 1 was related to female problem-solving success. Pairwise contrasts from the general generic model were nonsignificant after posthoc corrections for multiple comparisons (Table 3a), but in a dominance effect model, females with genotype AA were more likely to problem-solve than females with genotype AT or TT at α = 0.05 (Table 3b; Fig. 3a), and entered the nest box more times (dominance effect model, *β* = 1.714 ± 0.855, *Z* = 2.128, *P* = 0.045). With distance to paths/roads in the dominance model, there was an effect of both genotype (*β* = 1.694 ± 0.787, *Z* = 2.150, *P* = 0.031) and distance from paths/roads (*β* = −1.239 ± 0.588, *Z* = −2.106, *P* = 0.035) on female problem-solving success. We found no statistical support for an effect of SNP226 on male problem-solving success (Table S18). However, it was difficult to test the effect of SNP226 on male problem-solving, because only two males had genotype AA.

**Table 3:** Binomial GLMM for the relationship between nonsynonymous SNP226 in exon 1 and success on obstacle removal tests in female great tits.

(a) General generic model		*R*^2^*m* = 0.083		*R*^2^*c* = 0.280	
Fixed effects	*β* ± SE	*Z*	*P*	*χ*_2_^2^	*P*
Intercept	0.532 ± 0.851	0.625	0.532		
SNP226 AT vs AA	− 1.693 ± 0.872	− 1.942	0.127^a^		
SNP226 TT vs AA	− 1.551 ± 0.810	− 1.914	0.134^a^	5.238	0.072

Random effects	Variance	SD	N		
Individual	0.064	0.253	52		
Study site	0.832	0.912	3		

(b) Dominance effect model		*R*^2^*m* = 0.084		*R*^2^*c* = 0.280	
Fixed effects	*β* ± SE	*Z*	*P*		
Intercept	0.535 ± 0.849	0.630	0.528		
SNP226 (AT/TT vs AA)	− 1.609 ± 0.775	− 2.077	0.037		

Random effects	Variance	SD	N		
Individual	0.085	0.292	52		
Study site	0.811	0.901	3		

(a) General generic model, (b) dominance effect model with AT = TT.

^a^*P* values from posthoc Tukey test. *P* = 0.974 for the AT–TT contrast.

*N* = 73 observations on 52 females; 9 AA, 16 TA, 27 TT.

In addition, we found some evidence for an effect of SNP84 in exon 9 on problem-solving success, with this association being stronger in males than females (Table 4; Fig. 3b,c). In males, pairwise contrasts from the general generic model were nonsignificant after posthoc corrections for multiple comparisons (Table 4a), but in a dominance model males with genotype CC were more likely to problem-solve than males with CT or TT (*β* = 3.504 ± 0.1.526, *Z* = 2.296, *P* = 0.021; Table 4b), and tended to enter the nest box more times (*β* = 3.160 ± 1.807, *Z* = 1.749, *P* = 0.080). The effect size associated with the SNP84-problem-solving success association in males was large (*R*^2^*m* = 0.292, general generic model). In females, the relationship between SNP84 in exon 9 and problem-solving success was nonsignificant, but was in the same direction as the effect observed in males, and was associated with a substantial effect size, comparable to that for SNP226 in exon 1 (Table 4c, d). With distance to paths/roads in the dominance models, there was some evidence for an effect of both SNP84 (male: *β* = 3.727 ± 1.435, *Z* = 2.597, *P* = 0.009; females: *β* = 1.296 ± 0.7314, *Z* = −1.757, *P* = 0.087) and distance from paths/roads (males: *β* = −0.348 ± 0.205, *Z* = −1.694, *P* = 0.090; females: *β* = −0.190 ± 0.093, *Z* = −2.040, *P* = 0.041) on problem-solving success in both sexes. No other promoter region or exonic SNP was associated with problem-solving success in either females (Table S15, S16) or males (Table S17, S18).

**Table 4:** Binomial GLMM showing the relationship between synonymous SNP84 in exon 9 and success on the obstacle removal test in male and female great tits; (a) general generic model, males (b) dominance effect model, males with CT = TT, (c) general generic model, males (d) dominance effect model, females with CT = TT.

(a) General generic model, males		*R*^2^*m* = 0.292		*R*^2^*c* = 0.324	
Fixed effects	*β* ± SE	*Z*	*P*	*χ*_2_^2^	*P*
Intercept	1.531 ± 1.307	1.172	0.241		
SNP84 CT vs CC	− 3.771 ± 1.585	− 2.380	0.045^a^		
SNP84 TT vs CC	− 1.551 ± 0.810	− 1.914	0.134^a^	9.77	0.007

Random effects	Variance	SD	N		
Study site	0.155	0.394	3		

(b) Dominance effect, males		*R*^2^*m* = 0.277		*R*^2^*c* = 0.324	
Fixed effects	*β* ± SE	*Z*	*P*		
Intercept	1.595 ± 1.327	1.202	0.229		
SNP84 (CT/TT vs CC)	− 3.504 ± 1.526	− 2.296	0.021		

Random effects	Variance	SD	N		
Study site	0.228	0.478	3		

(c) General generic model, females		*R*^2^*m* = 0.092		*R*^2^*c* = 0.310	
Fixed Effects	*β* ± SE	*Z*	*P*	*χ*_2_^2^	*P*
Intercept	0.036 ± 0.752	0.049	0.960		
SNP84 CT vs CC	− 1.536 ± 0.801	− 1.917	0.133^a^		
SNP84 TT vs CC	− 0.624 ± 0.803	− 0.777	0.443^a^	4.969	0.083

Random effects	Variance	SD	N		
Individual	0.288	0.536	52		
Study site	0.753	0.868	3		

(d) Dominance model, females		*R*^2^*m* = 0.059		*R*^2^*c* = 0.296	
Fixed Effects	*β* ± SE	*Z*	*P*		
Intercept	0.036 ± 0.752	0.049	0.960		
SNP84 (TT/CT vs CC)	− 1.193 ± 0.708	− 1.684	0.092		

Random effects	Variance	SD	N		
Individual	0.306	0.553	52		
Study site	0.800	0.894	3		

^a^*P* values from posthoc Tukey test. *P* = 0.764, 0.717 for the CT–TT contrast in males and females, respectively.

*N* = 41 males, 5 CC, 20 CT, 16 TT (a, b); 73 observations on 52 females, 11 CC, 28 CT, 13 TT (c, d).

We added incorrect attempts and latency to contact the test apparatus to dominance models predicting female problem-solving success from SNP226 and SNP84, to investigate how considering these behavioral traits might modify the relationship between SNPs and problem-solving. In genotyped males, incorrect attempts and latency to contact unexpectedly did not have significant effects on problem-solving success (*P* > 0.20), so we did not add these variables to the model involving SNP84 and problem-solving in males. The effect of SNP226 on problem-solving success in females was no longer statistically significant (*β* = −1.066 ± 0.808, *Z* = −1.320, *P* = 0.186) when including these variables in the model, while both incorrect attempts (*β* = −0.048 ± 0.025, *Z* = −1.925, *P* = 0.054) and latency to contact (*β* = −0.01 ± 0.005, *Z* = −1.928, *P* = 0.054) had near significant effects. A similar pattern was observed when adding latency to contact and incorrect attempts to the model involving SNP84 and female problem-solving success.

### *SERT* polymorphisms, distribution patterns and breeding parameters

**Figure 4:**
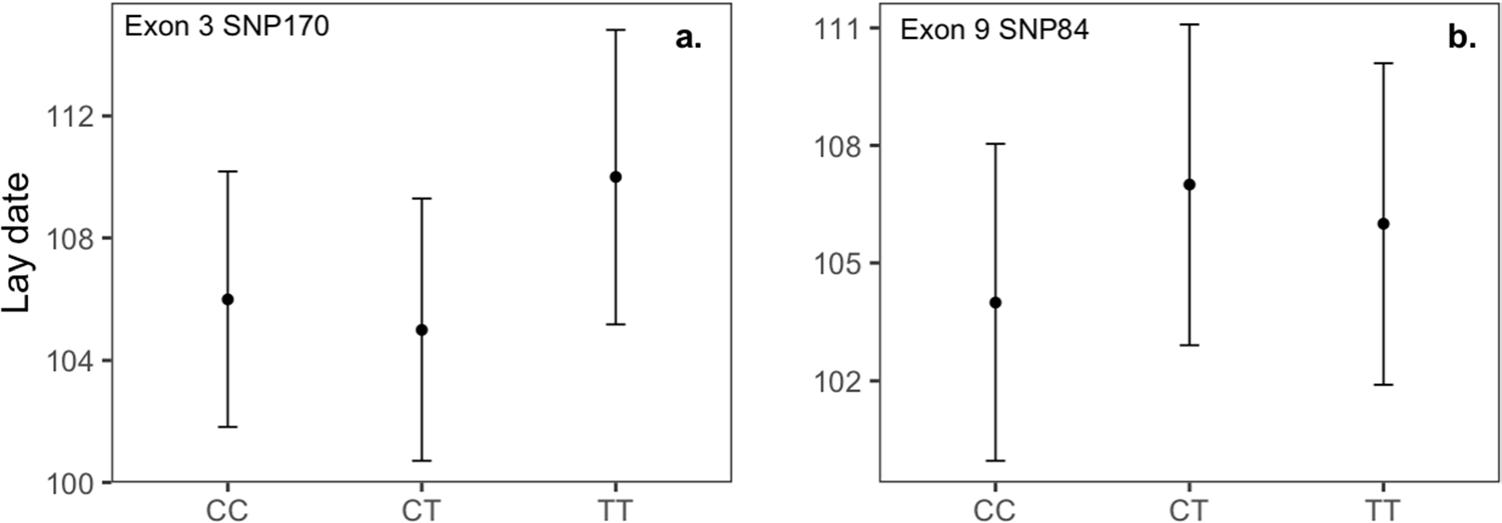
Relationships between *SERT* SNPs and lay date (days since January 1). Mean values and standard errors (error bars) for groups were extracted using R package emmeans and back transformed from the log scale; (**a**) exon 3 SNP170, *N* (observations, females) = CC: 99, 77; CG: 13, 11; GG: 5, 3; (**b**) exon 9 SNP84. *N* (observations, females) = CC: 25, 20; CT: 61, 47; TT: 31, 24.

We found no statistically significant relationships between any SNP and distance from paths/roads (Table S19, S20). SNP170 in exon 3 (*R*^2^*m* = 0.022, general generic model) and SNP84 in exon 9 (*R*^2^*m* = 0.016, general generic model) showed associations with laying date, but effect sizes were relatively small (Table 5a,b; Fig. 4; Table S21, S22 show models for all SNPs). In general generic models, posthoc pairwise comparisons were nonsignificant at α = 0.05 (Table 5). However, in dominance effects models, females with genotypes CG and CC at SNP170 in exon 3 lay earlier than females with GG (*β* = 0.041 ± 0.019 , *t*_114_ = 2.134, *P* = 0.034; Table 5a), and females with genotypes CT and TT at SNP84 in exon 9 lay later than females with CC (*β* = 0.022 ± 0.009 , *t*_74_ = 2.371, *P* = 0.020; Table 5b). We found no statistical support for relationships between SNPs and fledgling numbers (Tables S23–S26).

**Table 5:** General generic and dominance effect LMMs showing the relationship between, (a) SNP170 in exon 3, and (b) SNP84 in exon 9 and log-transformed laying date in female great tits.

(a) SNP170 exon 3							
General generic model		*R*^2^*m* = 0.017		*R*^2^*c* = 0.653			
Fixed effects	*β* ± SE	*t*	*d.f*	*P*	*F*	*d.f*	*P*
Intercept	4.66 ± 0.039	118.5	1.069	0.003			
SNP170 CG	− 0.039 ± 0.019	− 1.966	92.7	0.131^a^			
SNP170 CC	− 0.051 ± 0.022	2.285	58.5	0.065^a^	2.712	2.113	0.070

Random effects	Variance	SD	N				
Individual	< 0.001	< 0.001	91				
Study site	< 0.001	0.013	4				
Year	0.003	0.054	2				
Residual	0.002	0.041	117				

Dominance effect		*R*^2^*m* = 0.014		*R*^2^*c* = 0.654			
Fixed Effects	*β* ± SE	*t*	*d.f*	*P*			
Intercept	4.661 ± 0.039	118.6	1.075	0.003			
SNP170 (CG/CC vs GG)	0.041 ± 0.019	2.134	113.6	0.034			

Random effects	Variance	SD	N				
Individual	< 0.001	< 0.001	91				
Study site	< 0.001	0.014	4				
Year	0.002	0.054	2				
Residual	0.002	0.041	117				

(b) SNP84 exon 9							
General generic model		*R*^2^*m* = 0.016		*R*^2^*c* = 0.508			
Fixed effects	*β* ± SE	*t*	*d.f*	*P*	*F*	*d.f*	*P*
Intercept	4.646 ± 0.038	120.0	1.12	0.002			
SNP84 CT	− 0.024 ± 0.010	− 2.330	77.8	0.057^b^			
SNP84 TT	− 0.019 ± 0.011	− 1.664	80.3	0.225^b^	2.916	2,73	0.060

Random effects	Variance	SD	N				
Individual	< 0.001	0.008	91				
Study site	< 0.001	0.010	4				
Year	0.002	0.052	2				
Residual	0.002	0.040	117				

Dominance model		*R*^2^*m* = 0.016		*R*^2^*c* = 0.509			
Fixed effects	*β* ± SE	*t*	*d.f*	*P*			
Intercept	4.646 ± 0.038	120.1	1.12	0.002			
SNP84 (CT/TT vs CC)	0.022 ± 0.009	2.371	73.9	0.020			

Random effects	Variance	SD	N				
Individual	< 0.001	0.007	91				
Study site	< 0.001	0.010	4				
Year	0.002	0.052	2				
Residual	0.002	0.040	117				

^a^*P* values from posthoc Tukey test. *P* = 0.613 for the CC–CG contrast.

^b^*P* values from posthoc Tukey test. *P* = 0.864 for the CT–TT contrast.

*N* = 117 observations on 92 females; SNP170: 77 CC, 11 CG, 3 GG; SNP84: 20 CC, 47 CT, 24 TT.

## Discussion

We found relationships between *SERT* SNPs and multiple personality traits, problem-solving performance and breeding parameters. Only one of these relationships (between SNP36 in exon 6 and incorrect attempts in females) remained significant after adjusting for multiple comparisons, but several were associated with substantial effect sizes (*R*^2^ = 0.03–0.30). These relationships warrant further investigation, given the large sample sizes needed to robustly test for associations between behaviors and SNPs, each of which is expected to have a relatively small independent effect on behavioral or cognitive variation^13–15^.

Past studies have suggested that *SERT* polymorphisms might be involved in variation in neophobia, boldness and exploratory behavior, which are often interrelated traits^37,40^. We found some additional support for this hypothesis in the current study. With respect to neophobia behavior, the strongest gene-behavior association involved synonymous SNP36 in exon 6, which explained 4.8% of the variance in return latencies during novel object, but not baseline, trials. Heterozygous females (CT) were quicker to return to the nest box during novel object trials than homozygotes, suggesting that heterozygous females are less neophobic. However, SNP36 was not related to return latencies during baseline trials (boldness following disturbance at the nest), suggesting that SNP36 may be specifically related to neophobia, rather than affecting boldness-related responses across contexts (i.e. both neophobia and boldness following disturbance). The largest SNP-behavior association for exploratory behavior was with promoter region SNP438, which explained 3.5% of the behavioral variance, with heterozygotes (CT) having higher exploration scores than homozygotes. Our sample sizes for neophobia and exploratory behavior were similar to those in previous studies on great tits^37,40,41^. We thus had similar statistical power to detect a given effect size, and also found effect sizes similar to those reported by past studies^37,40,41^. However, past studies that found statistically significant relationships involved fewer SNPs, and thus lesser corrections for multiple comparisons. In sum, research on relationships between *SERT* polymorphisms, neophobia and exploratory behavior in great tits suggests no consistent sources of genetic control involving *SERT*, but rather that different *SERT* SNPs may be associated with behavioral variation in different populations. Also, despite interrelationships between neophobia, boldness and exploratory behavior, these traits were not related to the same SNPs, likely reflecting the fact that complex behavioral traits can be influenced by many different environmental and genetic effects^13–16^.

Our study represents a first attempt to characterize associations between problem solving and *SERT* polymorphisms in birds, and we found some evidence for such associations in the case of nonsynonymous SNP226 in exon 1 and synonymous SNP84 in exon 9. Nonsynonymous SNP226 in exon 1 was associated with 8% percent of the variance in female problem-solving success and 14% percent of the variance in female incorrect attempts during problem-solving. Females with rare genotype AA at SNP226 directed fewer incorrect attempts at the barrier and succeeded more on the test compared to those with genotype AT or TT. A higher rate of incorrect attempts directed at the barrier during the obstacle removal test (persistent pecking at the barrier) is associated with lower rates of success^61^. Therefore, SNP226 could affect problem-solving success by modulating the rate of incorrect attempts. Indeed, when including both incorrect attempts and latency to contact the test apparatus in statistical models, the effect of SNP226 on female problem-solving success was no longer statistically significant. This suggests that variance in both of these behavioral patterns could contribute to explaining the relationship between SNP226 and problem-solving success, although there was not a statistically significant relationship between SNP226 and latency to contact the test apparatus. The specific aspect of cognitive function which is reflected by incorrect attempts remains unclear, whereas latency to contact could be affected by both motivation and neophobia.

SNP226 is a nonsynonymous variant, and could thus affect serotonergic signaling, cognition and behavior during problem solving through direct effects on SERT protein structure and function. The E26D amino acid change caused by SNP226 is located within the intracellular N terminus of the SERT protein (Fig. S1). Extensive research has focused on the role of transmembrane helices and their connecting intracellular and extracellular loops in mediating substrate recognition and transport dynamics of transmembrane transport proteins, with relatively little work involving the function of transport protein tails (i.e. the N and C termini)^82^. However, emerging evidence suggests that the N and C termini can also play important roles in transporter function^82^, with research specifically indicating that the N terminus is involved in regulating transformational changes in SERT during substrate exchange^83,84^. However, SNAP2 analysis could not reliably predict functionality for the change in amino acid sequence from glutamic acid to aspartic acid caused by SNP226. Why relationships between SNP226, incorrect attempts and problem-solving success were present in females, but not males, also remains to be elucidated, but note that the low number of males with genotype AA at SNP226 made the effect of this SNP difficult to assess in males.

In addition to SNP226, synonymous SNP84 in exon 9 was also associated with problem-solving success, and there was some evidence for an effect of this SNP on problem-solving in both females and males. Specifically, 29% of the variance in male problem-solving and 9% of the variance in female problem-solving was associated with this SNP, although this effect was statistically nonsignificant in females at α = 0.05. SNP84 was not significantly related to the other behavioral patterns that we measured during the obstacle removal test, and might thus affect problem-solving through effects on traits not quantified through our protocol.

Synonymous SNP36 in exon 6 and synonymous SNP187 in exon 1 were also associated with female incorrect attempts directed at the barrier, although neither of these SNPs was significantly related to problem-solving success. The relationship between incorrect attempts on the barrier and SNP36 was associated with the largest effect size (*R*^2^ = 0.30) found in our study, and remained significant after accounting for multiple comparisons. As was also the case for neophobia behavior, females heterozygous at SNP36 differed in behavior from homozygotes, pecking the barrier more. Thus, one might expect that SNP36 could mediate an association between incorrect attempts and neophobia behavior. However, we did not find a statistically significant association between incorrect attempts and neophobia behavior. The relationship between SNP187 in exon 1 and incorrect attempts was associated with a smaller, but non-negligible, effect size (*R*^2^ = 0.108). A different nucleotide change at position 187 in exon 1 (A/T instead of C/T) was previously related to hissing behavior during nest defense in female great tits^41^. However, recent work in our study population showed no relationship between SNP187 and either hissing behavior or female-female aggression^58^. Thus, whether SNPs at this locus have significant behavioral effects across multiple traits remains equivocal. In our dataset, linkage between SNP226 and SNP187 in exon 1 made the effects of the two difficult to disentangle.

We hypothesized that genetic differences between birds could explain reduced neophobia and enhanced problem-solving among individuals occupying highly disturbed territories. However, we found no association between *SERT* SNPs and distance from roads/paths. Also, neophobia and problem-solving were not significantly associated. Rather, distance from roads/paths and *SERT* SNPs had independent effects on neophobia (for SNP36 exon 6) and problem-solving (for SNP226 exon 1 and SNP84 exon 9). Thus, multiple environmental and genetic effects may combine to determine behavior and problem-solving. Genotype-by-environment interactions also remain possible, but we had an insufficient sample size to test this possibility.

SNP170 in exon 3 and SNP84 in exon 9 showed associations with egg laying date, suggesting that *SERT* polymorphisms could affect this reproductive trait. However, these relationships had relatively small effect sizes (explaining ~ 2% of the variance), and there was no evidence for a relationship between SNPs and fledgling numbers. Egg laying date in great tits can be important to matching timing of the nestling period with peak caterpillar abundance, and can affect reproductive success^41,85^. One past study in great tits reported that two different *SERT* SNPs in exon 8 (not present in our population) correlated with laying date and nestlings hatched^41^. Relationships between *SERT* polymorphisms and laying date could be mediated through multiple pathways, including effects of serotonin on feeding behavior and endocrine systems, both of which affect the time at which individuals attain breeding condition^41^.

Besides SNP226 in exon 1, the SNPs for which we found associations with behavioral and fitness traits were synonymous SNPs. Synonymous SNPs do not affect the amino acid sequence of the SERT protein, and thus appear unlikely to affect SERT’s structure and enzymatic function. However, recent research suggests that synonymous SNPs can affect the expression level, structure and function of proteins, through mechanisms including effects on transcription, splicing and folding of proteins^86^. Alternatively, synonymous SNPs may be linked to functional variants. Synonymous SNPs are as frequently linked to disease in humans as are nonsynonymous SNPs^86^, and in birds nonsynonymous and synonymous SNPs have been linked to behavioral variation at similar rates^35,36^. In great tits, all of the past studies on *SERT*^37,40,41^ (and *DRD4*^23,27–29,36^) polymorphisms that have linked SNPs to behavioral variation have involved synonymous SNPs. These past results and our own findings suggest that synonymous SNPs may be linked to a nontrivial amount of genetic variation in behavioral traits.

## Conclusions

Our study revealed associations between *SERT* polymorphisms, personality traits, problem-solving and laying date. Associations were mostly non-significant after corrections for multiple comparisons, but some were associated with substantial effect sizes. Relationships between *SERT*, behavior and cognition in birds warrant further investigation, given the complex genetic control of behavior and large sample sizes required to robustly test for effects of any one genetic variant. Our study serves as a basis for future research, including meta-analyses aimed at synergizing evidence for effects of *SERT* on behavior, cognition and fitness.

## Supplementary Information

Supplementary Information.

## Data Availability

Data will be available through the Dryad Digital Repository^87^.
