# Supplementary material for: Serotonin transporter (SERT) polymorphisms, personality and problem-solving in urban great tits
Source: Sci Rep. 2021 Dec 20;11:24270. doi: 10.1038/s41598-021-03466-7 (PMC8688470; doi:10.1038/s41598-021-03466-7)
Supplement: Supplementary file 1 — Supplementary Information. [file 41598_2021_3466_MOESM1_ESM.docx]

**Online Supplementary Material**

**Serotonin transporter (*SERT*) polymorphisms, personality and problem-solving in urban great tits**

Andrea S. Grunst^1,2*#^, Melissa L. Grunst^1,2#^, Nicky Staes^1,3^, Bert Thys^1^, Rianne Pinxten^1,4^, Marcel Eens^1^

^1^Department of Biology, Behavioural Ecology and Ecophysiology Group, University of Antwerp, Belgium

^2^Littoral Environnement et Sociétés, La Rochelle Université, La Rochelle, France

^3^Centre for Research and Conservation, Royal Zoological Society of Antwerp, Antwerp, Belgium

^4^Faculty of Social Sciences, Antwerp School of Education, University of Antwerp, Belgium

**Caption:** We investigated the relationship between single nucleotide polymorphisms (SNPs) in the promoter and exonic regions of the serotonin transporter (*SERT*) gene and personality and problem-solving traits in urban populations of great tits (*Parus major*). We additionally explored whether *SERT* polymorphisms correlated to fitness-related breeding parameters. *SERT* is a good candidate gene for affecting behavioral and cognitive traits, because the serotonin transporter (SERT) is responsible for uptake of serotonin at synopses and thus has major effects on the serotonergic signaling pathway. We identified 26 SNPs in the promoter region and thirteen exons of the great tit *SERT* gene, and related 12 of these SNPs (which met criteria for statistical testing) to a suite of behavioral and problem-solving traits, and to breeding parameters. None of the SNPs that we identified had been previously reported in other great tit populations, although a different nucleotide substitution at the same position (187) of exon 1 was previously reported (Timm et al. 2019). These results indicate that the same *SERT* polymorphisms are not responsible for behavioral variation across different great tit populations. We identified some relationships between behaviors and laying date and SNPs in *SERT*, some of which were associated with relatively large effect sizes for behavioral traits. However, only one of these relationships was significant after correcting for multiple comparisons. Given the large sample sizes needed to robustly test for the effects of genetic variants on behavioral traits, we urge more research on the relationship between variation in the *SERT* gene and behavioral variation in birds. This Supplementary Material contains results primer sequences used to amplify the *SERT* promoter region and 13 exons (Table S1), results regarding patterns of linkage disequilibrium between SNPs (Table S2), a figure generated by the web-based application Protter showing the location of nonsynonymous SNPs within the great tit SERT (Figure S1), and results of linear and generalized linear mixed effects models (LMMs, GLMMs; Table S3-S24) that were too extensive to include in the manuscript.

**Table S1**. Sequences and product length (PL) for the primers used to amplify the promoter region and 13 exons of the great tit *SERT* gene.

| **Region** | **Forward primer** | **Reverse primer** | **PL (bp)** |
| --- | --- | --- | --- |
| Promoter | CATCTTCTCCTTTGCTACAGCC | ACAGAGCCTCAGAAGTTAGTTGA | 470 |
| Exon 1 | AAAGGTGTGGGAAGACAAA | CTGTCATTATCCTATCCC | 456 |
| Exon 2 | GGTGTATCTGGAGTTTTAGGAAAG | ATGAGAAATCCCACAGTAATTCAG | 134 |
| Exon 3 | CAAGATTTGAAGTGATTTGAAGTGA | TATCTCTGAAGTCACAAGAAATGC | 219 |
| Exon 4 | CTTTATTGCTTGGATAGGAGTAGC | GAAATGTCGTGATTTTGAAAGCTG | 138 |
| Exon 5 | AAGCTAAATTGAGGGTGGACT | GCTCTCTGGGCAGGAACAAC | 134 |
| Exon 6 | ATTTTGAGGTAGACAACACAGGA | CTAAGATTGTGTCAGAAGTGCAA | 103 |
| Exon 7 | CAATCTGGCTTGTAATCATGGTA | GATACAGCCAGCATTCAATCC | 127 |
| Exon 8 | CAGCATGACAGTGACAAATCTC | GGACACTTTTACACAATACAGCT | 112 |
| Exon 9 | TCATTTTGCAACATATTTCCTAGTG | CACTAAATCCCCACCCTAAAGA | 131 |
| Exon 10 | CTCCTGTTACTTTTAGATGCCTG | CTGTAAAATCCCCTTTGTTCACT | 96 |
| Exon 11 | GTCCTTAAAACTGTGCTTTAGTAAC | GCATCTAAAAGTAACAGGAGGTATA | 100 |
| Exon 12 | AAAGCGTGTGCCATCAAA | CCTAAGAGTCAAACAAGCAAC | 167 |
| Exon 13 | TGCTCAGTTCAGCCTGTTGGA | CAGAGGCCTGAAACGCTCCT | 74 |

**Table S2. Results regarding patterns of linkage disequilibrium:**  We assessed patterns of linkage disequilibrium among SNPs using the web-based application SNPStats, which uses matrices of genotype data to calculate haplotypes, linkage disequilibrium statistics and associated *P-*values based on Chi-square tests. These statistics (*D, D'*, Pearson’s *r*, *P*-values) are given in the tables below.

**
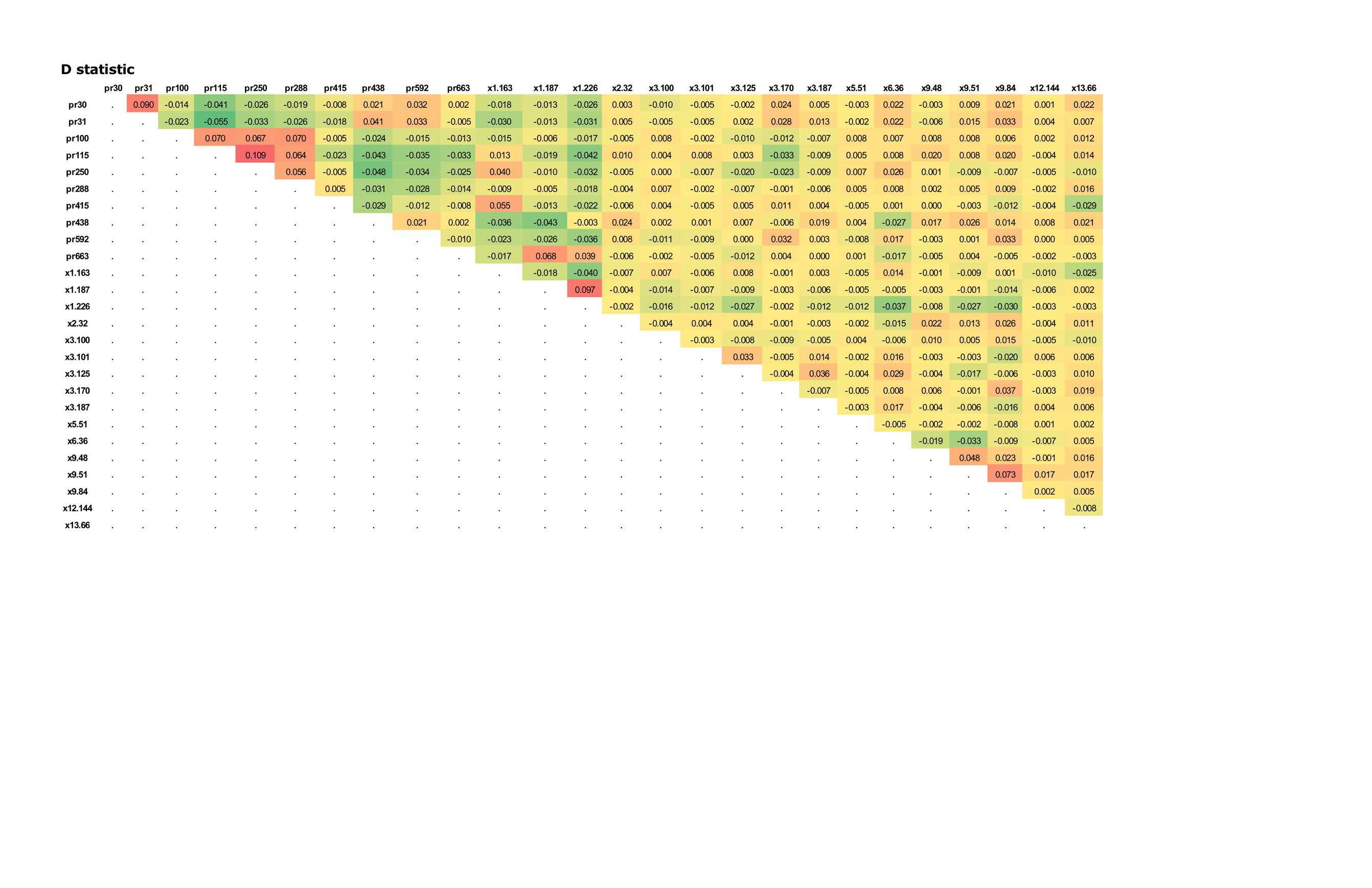
**

*Darker red colors indicate larger positive values and darker green colors indicate larger negative values

**
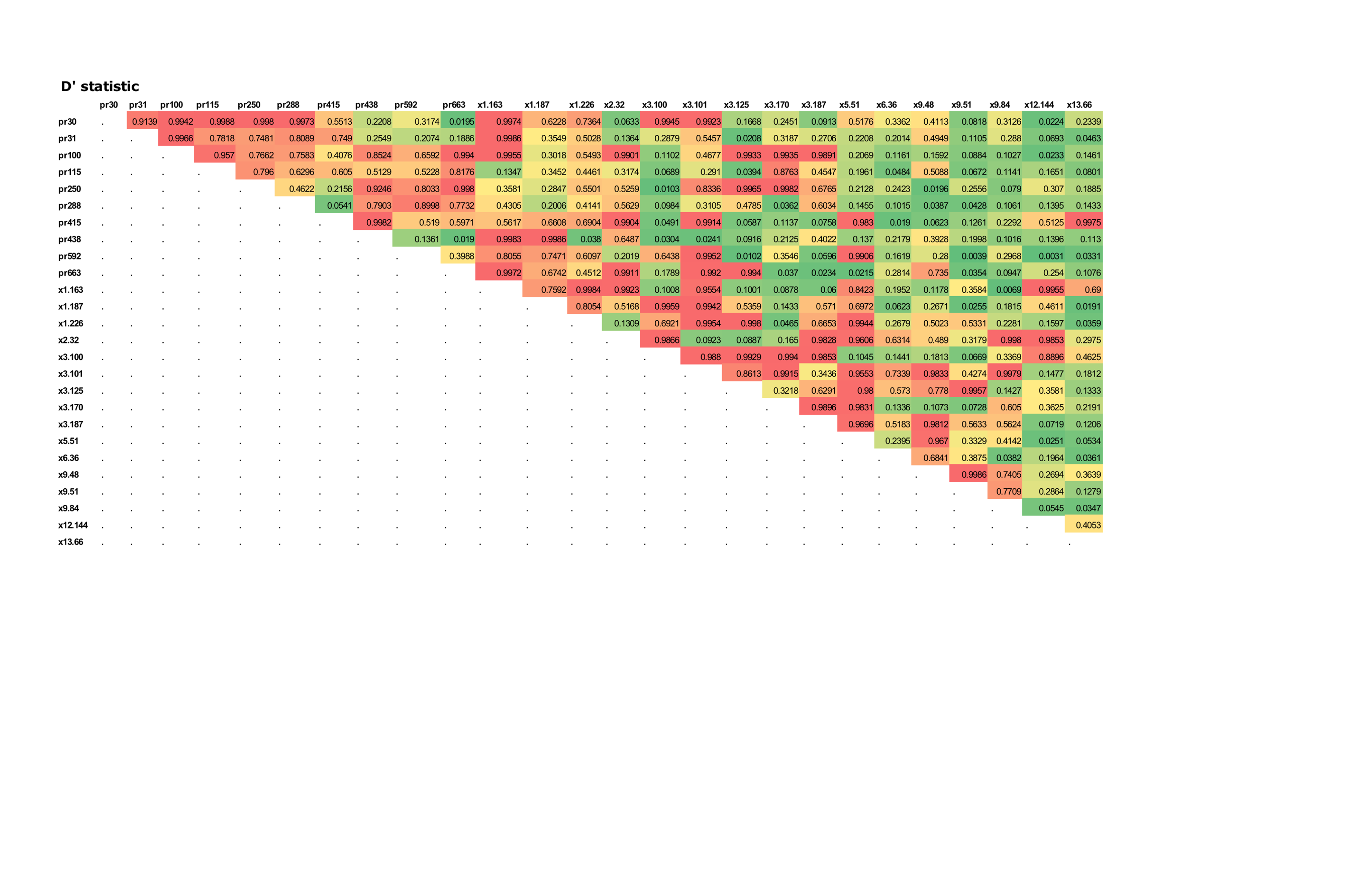
**

*Darker red values are closer to 1 and darker green values are closer to 0.

**
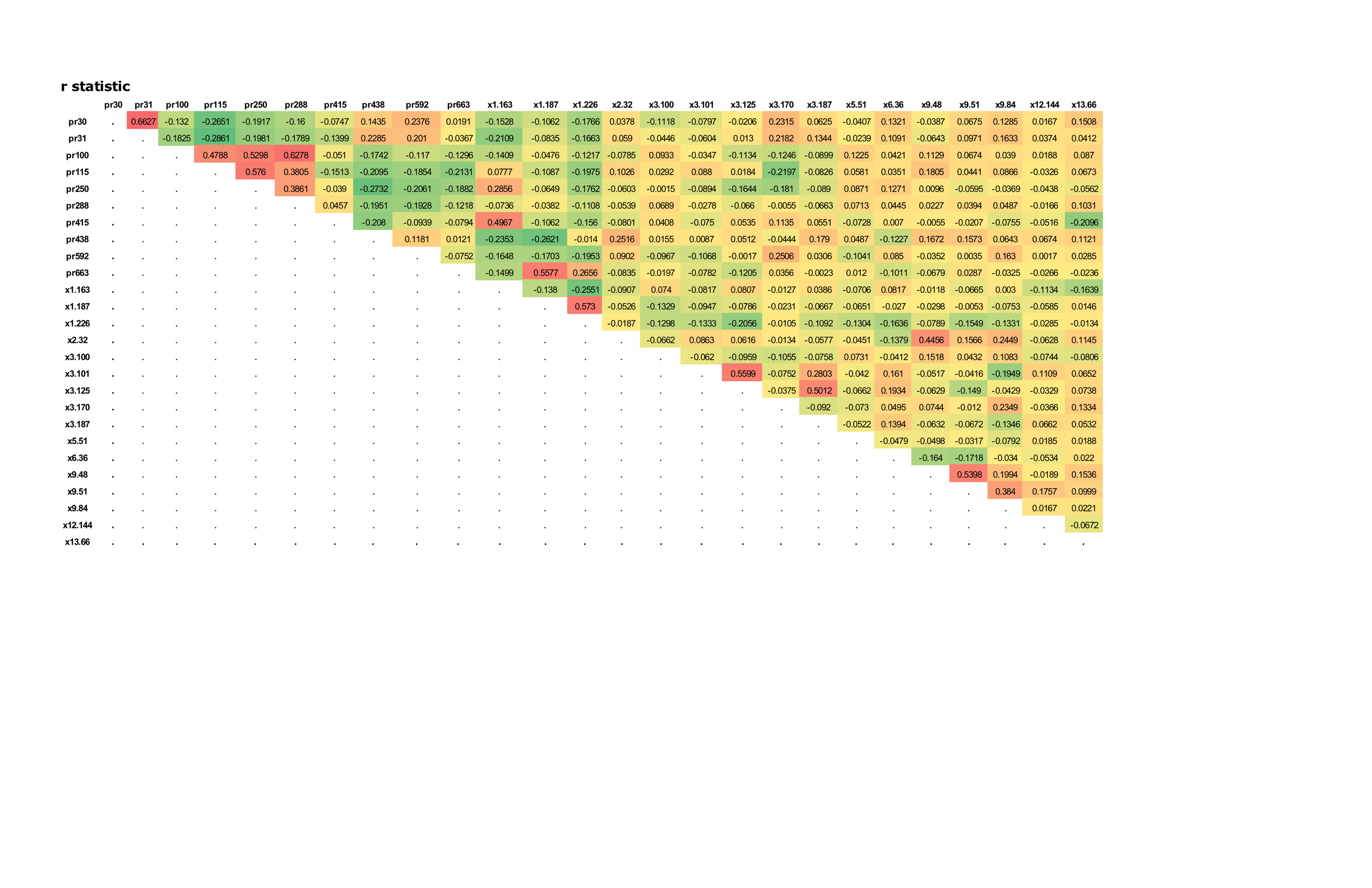
**

*Darker red colors indicate larger positive values and darker green colors indicate larger negative values.

**
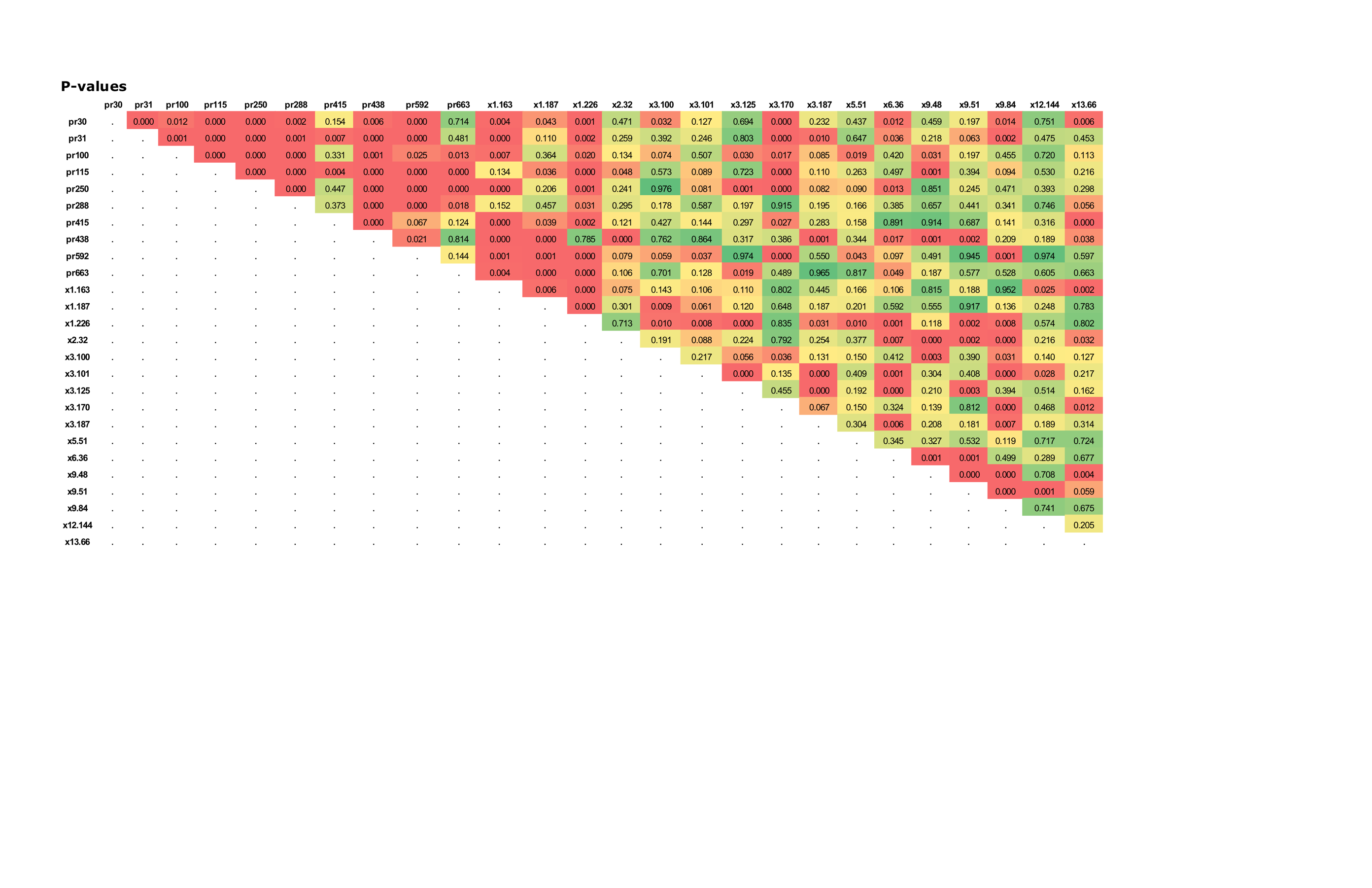
**

*Darker red colors are closer to 0 and darker green colors are closer to 1

**Figure S1**. Location of amino acid changes caused by three nonsynonymous SNPs within the great tit SERT protein, derived from the web-based application Protter (Omasits et al. 2013, *Bioinformatics*). The E26D amino acid change caused by SNP226 in exon 1 is located within the N-terminus, whereas the changes A231V and L260Q caused by SNP100 and SNP187 in exon 3, respectively, are located within extracellular loop 2.

**Table S3.** General generic LMMs for relationships between four SNPs in the promoter region and latency of female great tits to return to the nest during novel object tests. Each SNP was entered into a separate model: a) SNP31, b) SNP100, c) SNP250, d) SNP438.

| 1. **SNP31** |  |  |  |  |  |  |  |
| --- | --- | --- | --- | --- | --- | --- | --- |
| **Fixed effects** | ***β* ± SE** | ***t*** | ***d.f.*** | ***P*** | ***F*** | ***d.f.*** | ***P*** |
| Intercept | 2.45 ± 0.115 | 21.2 | 154 | < 0.001 |  |  |  |
| SNP31 AT | -0.118 ± 0.212 | -0.558 | 167 | 0.577 |  |  |  |
| SNP31 TT | -0.238 ± 0.421 | -0.566 | 181 | 0.571 | 0.437 | 2,98.8 | 0.647 |
| Trial | 0.718 ± 0.214 | 3.36 | 154 | <0.001 | 16.1 | 1,156 | <0.001 |
| Trial order | -0.104 ± 0.139 | -0.749 | 162 | 0.454 | 0.561 | 1,162 | 0.454 |
| SNP31 AT × trial | -0.035 ± 0.216 | -0.16 | 157 | 0.873 |  |  |  |
| SNP31 TT × trial | 0.747 ± 0.463 | 1.611 | 156 | 0.1092 | 1.37 | 2,156 | 0.258 |
| **Random effects** | **Variance** | **SD** | **N** |  |  |  |  |
| Individual | 0.306 | 0.554 | 81 |  |  |  |  |
| Study site | <0.001 | <0.001 | 4 |  |  |  |  |
| Year | <0.001 | <0.001 | 2 |  |  |  |  |
| Residual | 0.478 | 0.691 | 227 |  |  |  |  |
| 1. **SNP100** |  |  |  |  |  |  |  |
| **Fixed effects** | ***β* ± SE** | ***t*** | ***d.f.*** | ***P*** | ***F*** | ***d.f.*** | ***P*** |
| Intercept | 2.40 ± 0.114 | 20.9 | 6.23 | <0.001 |  |  |  |
| SNP100 AG | 0.123 ± 0.238 | 0.518 | 159 | 0.604 |  |  |  |
| SNP100 GG | -0.833 ± 0.576 | -1.44 | 126 | 0.150 | 0.854 | 2,84 | 0.429 |
| Trial | 0.759 ± 0.205 | 3.71 | 156 | <0.001 | 12.3 | 1,154 | <0.001 |
| Trial order | -0.117 ± 0.139 | -0.847 | 163 | 0.398 | 0.717 | 1,164 | 0.398 |
| SNP100 AG × trial | -0.037 ± 0.243 | -0.155 | 162 | 0.876 |  |  |  |
| SNP100 GG × trial | 0.518 ± 0.571 | 0.909 | 147 | 0.364 | 0.438 | 2,155 | 0.645 |
| **Random effects** | **Variance** | **SD** | **N** |  |  |  |  |
| Individual | 0.313 | 0.559 | 81 |  |  |  |  |
| Study site | <0.001 | 0.002 | 4 |  |  |  |  |
| Year | 0.003 | 0.054 | 2 |  |  |  |  |
| Residual | 0.470 | 0.686 | 227 |  |  |  |  |
| 1. **SNP250** | |  |  |  |  |  |  |
| **Fixed effects** | ***β* ± SE** | ***t*** | ***d.f.*** | ***P*** | ***F*** | ***d.f.*** | ***P*** |
| Intercept | 2.16 ± 0.299 | 7.21 | 74.8 | <0.001 |  |  |  |
| SNP250 AG | 0.113 ± 0.344 | 0.329 | 161 | 0.742 |  |  |  |
| SNP250 GG | 0.263 ± 0.316 | 0.833 | 156 | 0.406 | 0.883 | 2,70 | 0.418 |
| Trial | 0.609 ± 0.343 | 1.77 | 163 | 0.078 | 3.85 | 1,166 | <0.001 |
| Trial order | -0.086 ± 0.134 | -0.643 | 175 | 0.521 | 0.413 | 1,175 | 0.521 |
| SNP250 AG × trial | 0.196 ± 0.356 | 0.550 | 166 | 0.582 |  |  |  |
| SNP250 GG × trial | 0.132 ± 0.322 | 0.411 | 162 | 0.681 | 0.152 | 2,167 | 0.858 |
| **Random effects** | **Variance** | **SD** | **N** |  |  |  |  |
| Individual | 0.295 | 0.544 | 87 |  |  |  |  |
| Study site | 0.003 | 0.055 | 4 |  |  |  |  |
| Year | 0.003 | 0.058 | 2 |  |  |  |  |
| Residual | 0.482 | 0.695 | 244 |  |  |  |  |
| 1. **SNP438** |  |  |  |  |  |  |  |
| **Fixed effects** | ***β* ± SE** | ***t*** | ***d.f.*** | ***P*** | ***F*** | ***d.f.*** | ***P*** |
| Intercept | 2.31 ± 0.135 | 17.1 | 6.33 | <0.001 |  |  |  |
| SNP438 TC | 0.179 ± 0.196 | 0.916 | 159 | 0.361 |  |  |  |
| SNP438 TT | -0.285 ± 0.363 | -0.787 | 175 | 0.432 | 0.044 | 2,89 | 0.957 |
| Trial | 0.868 ± 0.210 | 4.14 | 167 | <0.001 | 17.6 | 1,168 | <0.001 |
| Trial order | -0.107 ± 0.132 | -0.808 | 174 | 0.420 | 0.654 | 1,174 | 0.420 |
| SNP438 TC × trial | -0.362 ± 0.194 | -1.86 | 163 | 0.063 |  |  |  |
| SNP438 TT × trial | 0.400 ± 0.346 | 1.15 | 164 | 0.248 | 3.01 | 2, 164 | 0.052 |
| **Random effects** | **Variance** | **SD** | **N** |  |  |  |  |
| Individual | 0.314 | 0.560 | 87 |  |  |  |  |
| Study site | <0.001 | <0.001 | 4 |  |  |  |  |
| Year | 0.007 | 0.086 | 2 |  |  |  |  |
| Residual | 0.464 | 0.681 | 244 |  |  |  |  |

**Reference genotypes are AA for SNP31, SNP100, SNP250 and CC for SNP438.*

**Table S4**. General generic LMMs for the relationship between eight exonic SNPs and latency of female great tits to return to the nest during novel object tests. Separate models were performed for each of eight SNPs: a) SNP163 exon1, b) SNP187 exon 1, c) SNP226 exon 1, d) SNP170 exon 3, e) SNP36 exon 6, f) SNP51 exon 9, g) SNP84 exon9, h) SNP66 exon 13.

| 1. **SNP 163 exon 1** | | | |  |  |  |  |
| --- | --- | --- | --- | --- | --- | --- | --- |
| **Fixed effects** | ***β* ± SE** | ***t*** | ***d.f.*** | ***P*** | ***F*** | ***d.f.*** | ***P*** |
| Intercept | 2.51 ± 0.457 | 5.507 | 127 | <0.001 |  |  |  |
| SNP163 AG | -0.052 ± 0.489 | -0.107 | 180 | 0.915 |  |  |  |
| SNP163 GG | -0.129 ± 0.461 | -0.28 | 181 | 0.779 | 0.204 | 2, 106 | 0.816 |
| Trial | 0.972 ± 0.496 | 1.959 | 165 | 0.051 | 10.7 | 1, 170 | 0.001 |
| Trial order | -0.061 ± 0.128 | -0.48 | 179 | 0.631 | 0.230 | 1, 180 | 0.631 |
| SNP163 AG × trial | -0.411 ± 0.520 | -0.791 | 165 | 0.430 |  |  |  |
| SNP163 GG × trial | -0.224 ± 0.491 | -0.456 | 164 | 0.648 | 0.496 | 2, 170 | 0.609 |
| **Random effects** | **Variance** | **SD** | **N** |  |  |  |  |
| Individual | 0.345 | 0.587 | 91 |  |  |  |  |
| Study site | <0.001 | <0.001 | 4 |  |  |  |  |
| Year | 0.016 | 0.125 | 2 |  |  |  |  |
| Residual | 0.460 | 0.678 | 255 |  |  |  |  |
| 1. **SNP187 exon 1** | |  |  |  |  |  |  |
| **Fixed effects** | ***β* ± SE** | ***t*** | ***d.f.*** | ***P*** | ***F*** | ***d.f.*** | ***P*** |
| Intercept | 2.337 ± 0.154 | 15.106 | 4.8 | <0.001 |  |  |  |
| SNP187 TC | 0.259 ± 0.199 | 1.298 | 188.7 | 0.196 | 1.949 | 2, 103.1 | 0.147 |
| SNP187 TT | -0.077 ± 0.524 | -0.148 | 180.7 | 0.882 |  |  |  |
| Trial | 0.627 ± 0.107 | 5.847 | 173.7 | <0.001 | 6.329 | 1, 167.5 | 0.012 |
| Trial order | 0.005 ± 0.044 | 0.115 | 33.1 | 0.909 | 0.013 | 1, 33.1 | 0.909 |
| SNP187 TC × trial | 0.042 ± 0.194 | 0.220 | 172.6 | 0.826 | 0.554 | 2, 169.7 | 0.575 |
| SNP187 TT × trial | -0.529 ± 0.529 | -1.000 | 167.0 | 0.319 |  |  |  |
| **Random effects** | **Variance** | **SD** | **N** |  |  |  |  |
| Individual | 0.336 | 0.580 | 91 |  |  |  |  |
| Study site | <0.001 | <0.001 | 4 |  |  |  |  |
| Year | 0.009 | 0.095 | 2 |  |  |  |  |
| Residual | 0.452 | 0.672 | 254 |  |  |  |  |
| 1. **SNP226 exon 1** | |  |  |  |  |  |  |
| **Fixed effects** | ***β* ± SE** | ***t*** | ***d.f.*** | ***P*** | ***F*** | ***d.f.*** | ***P*** |
| Intercept | 2.637 ± 0.273 | 9.646 | 43.7 | <0.001 |  |  |  |
| SNP226 AT | -0.171 ± 0.293 | -0.582 | 172.8 | 0.562 |  |  |  |
| SNP226 TT | -0.349 ± 0.283 | -1.236 | 171.5 | 0.218 | 1.236 | 2,98 | 0.294 |
| Trial | 0.560 ± 0.245 | 2.278 | 165.5 | 0.024 | 33.92 | 1,169 | <0.001 |
| Trial order | 0.010 ± 0.451 | 0.229 | 34.0 | 0.821 | 0.052 | 1,34 | 0.820 |
| SNP226 AT × Trial | 0.083 ± 0.284 | 0.293 | 166.8 | 0.770 |  |  |  |
| SNP226 TT × Trial | 0.064 ± 0.275 | 0.235 | 167.4 | 0.814 | 0.042 | 2,169 | 0.958 |
| **Random effects** | **Variance** | **SD** | **N** |  |  |  |  |
| Individual | 0.340 | 0.583 | 91 |  |  |  |  |
| Study site | <0.001 | <0.001 | 4 |  |  |  |  |
| Year | 0.009 | 0.098 | 2 |  |  |  |  |
| Residual | 0.456 | 0.675 | 254 |  |  |  |  |
| 1. **SNP170 exon 3** | |  |  |  |  |  |  |
| **Fixed effects** | ***β* ± SE** | ***t*** | ***d.f.*** | ***P*** | ***F*** | ***d.f.*** | ***P*** |
| Intercept | 2.45 ± 0.143 | 17.1 | 2.17 | 0.002 |  |  |  |
| SNP163 AG | -0.032 ± 0.255 | -0.126 | 207 | 0.899 |  |  |  |
| SNP163 GG | -0.435 ± 0.421 | -1.03 | 142 | 0.303 | 1.16 | 2,94 | 0.318 |
| Trial | 0.767 ± 0.189 | 4.05 | 176 | <0.001 | 10.2 | 1,174 | 0.001 |
| Trial order | -0.098 ± 0.127 | -0.773 | 183 | 0.440 | 0.597 | 1,183 | 0.440 |
| SNP163AG × trial | 0.036 ± 0.251 | 0.143 | 175 | 0.886 |  |  |  |
| SNP163GG × trial | -0.227 ± 0.384 | -0.591 | 169 | 0.555 | 0.195 | 2,172 | 0.822 |
| **Random effects** | **Variance** | **SD** | **N** |  |  |  |  |
| Individual | 0.345 | 0.587 | 91 |  |  |  |  |
| Study site | <0.001 | <0.001 | 4 |  |  |  |  |
| Year | 0.016 | 0.125 | 2 |  |  |  |  |
| Residual | 0.460 | 0.678 | 255 |  |  |  |  |
| 1. **SNP36 exon 6** | |  |  |  |  |  |  |
| **Fixed effects** | ***β* ± SE** | ***t*** | ***d.f.*** | ***P*** | ***F*** | ***d.f.*** | ***P*** |
| Intercept | 2.40 ± 0.205 | 11.7 | 17.3 | <0.001 |  |  |  |
| SNP36 CT | 0.045 ± 0.230 | 0.198 | 193 | 0.843 |  |  |  |
| SNP36 TT | -0.033 ± 0.245 | -0.138 | 177 | 0.890 | 0.635 | 2,104 | 0.532 |
| Trial | 0.918 ± 0.242 | 3.78 | 173 | <0.001 | 18.1 | 1,174 | <0.001 |
| Trial order | -0.062 ± 0.127 | -0.497 | 181 | 0.619 | 0.247 | 1,181 | 0.620 |
| SNP36 CT × trial | -0.447 ± 0.227 | -1.97 | 172 | 0.049 |  |  |  |
| SNP36 TT × trial | 0.029 ± 0.239 | 0.125 | 172 | 0.900 | 3.57 | 2,173 | 0.030 |
| **Random effects** | **Variance** | **SD** | **N** |  |  |  |  |
| Individual | 0.352 | 0.593 | 93 |  |  |  |  |
| Study site | <0.001 | <0.001 | 4 |  |  |  |  |
| Year | 0.013 | 0.114 | 2 |  |  |  |  |
| Residual | 0.451 | 0.671 | 259 |  |  |  |  |
| 1. **SNP51 exon 9** | |  |  |  |  |  |  |
| **Fixed effects** | ***β* ± SE** | ***t*** | ***d.f.*** | ***P*** | ***F*** | ***d.f.*** | ***P*** |
| Intercept | 2.30 ± 0.134 | 17.1 | 2.8 | <0.001 |  |  |  |
| SNP51 CT | 0.331 ± 0.229 | 1.44 | 192.9 | 0.150 |  |  |  |
| SNP51 TT | 0.377 ± 0.380 | 0.993 | 151.8 | 0.322 | 1.92 | 2,97 | 0.152 |
| Trial | 0.766 ± 0.196 | 3.91 | 176.4 | <0.001 | 10.9 | 1,176 | <0.001 |
| Trial order | -0.090 ± 0.129 | -0.699 | 183.3 | 0.485 | 0.488 | 1,183 | 0.485 |
| SNP51 CT × trial | 0.023 ± 0.224 | 0.106 | 172.8 | 0.915 |  |  |  |
| SNP51 TT × trial | -0.214 ± 0.381 | -0.564 | 175.7 | 0.573 | 0.174 | 2,174 | 0.537 |
| **Random effects** | **Variance** | **SD** | **N** |  |  |  |  |
| Individual | 0.319 | 0.565 | 92 |  |  |  |  |
| Study site | <0.001 | <0.001 | 4 |  |  |  |  |
| Year | 0.015 | 0.122 | 2 |  |  |  |  |
| Residual | 0.470 | 0.686 | 257 |  |  |  |  |
| 1. **SNP84 exon 9** | |  |  |  |  |  |  |
| **Fixed effects** | ***β* ± SE** | ***t*** | ***d.f.*** | ***P*** | ***F*** | ***d.f.*** | ***P*** |
| Intercept | 2.37 ± 0.201 | 11.787 | 10.3 | <0.001 |  |  |  |
| SNP84 CT | 0.022 ± 0.220 | 0.102 | 184 | 0.919 |  |  |  |
| SNP84 TT | 0.043 ± 0.248 | 0.176 | 178 | 0.860 | 0.071 | 2,103 | 0.931 |
| Trial | 0.840 ± 0.232 | 3.619 | 171 | <0.001 | 15.7 | 1,175 | <0.001 |
| Trial order | -0.079 ± 0.129 | -0.617 | 181 | 0.538 | 0.380 | 1,181 | 0.538 |
| SNP84 CT × trial | -0.061 ± 0.218 | -0.282 | 171 | 0.778 |  |  |  |
| SNP84 TT × trial | -0.227 ± 0.246 | -0.922 | 172 | 0.357 | 0.474 | 2,173 | 0.623 |
| **Random effects** | **Variance** | **SD** | **N** |  |  |  |  |
| Individual | 0.340 | 0.583 | 92 |  |  |  |  |
| Study site | <0.001 | <0.001 | 4 |  |  |  |  |
| Year | 0.018 | 0.134 | 2 |  |  |  |  |
| Residual | 0.468 | 0.685 | 259 |  |  |  |  |
| 1. **SNP66 exon 13** | |  |  |  |  |  |  |
| **Fixed effects** | ***β* ± SE** | ***t*** | ***d.f.*** | ***P*** | ***F*** | ***d.f.*** | ***P*** |
| Intercept | 2.405 ± 0.185 | 12.99 | 4.499 | <0.001 |  |  |  |
| SNP66 CT | 0.038 ± 0.196 | 0.193 | 177 | 0.847 |  |  |  |
| SNP66 TT | -0.135 ± 0.364 | -0.373 | 144 | 0.710 | 0.747 | 2,90 | 0.477 |
| Trial | 0.506 ± 0.119 | 4.274 | 146 | <0.001 | 31.9 | 1,162 | <0.001 |
| Trial order | -0.002 ± 0.047 | -0.049 | 61.6 | 0.961 | 0.002 | 1,62 | 0.961 |
| SNP66 CT × trial | 0.315 ± 0.195 | 1.611 | 167 | 0.109 |  |  |  |
| SNP66 TT × trial | 0.336 ± 0.346 | 0.971 | 159 | 0.333 | 1.51 | 2,162 | 0.224 |
| **Random effects** | **Variance** | **SD** | **N** |  |  |  |  |
| Individual | 0.344 | 0.586 | 88 |  |  |  |  |
| Study site | <0.001 | <0.001 | 4 |  |  |  |  |
| Year | 0.020 | 0.141 | 2 |  |  |  |  |
| Residual | 0.466 | 0.682 | 246 |  |  |  |  |

**Reference genotypes are AA for SNP163 exon 1, SNP226 exon 1, and SNP170 exon 3, and CC for SNP187 exon 1, SNP36 exon 6, SNP51 exon 9, SNP84 exon 9 and SNP66 exon 13.*

**Table S5**. General generic GLMMs with Poisson distributions for relationships between four SNPs in the promoter region and the novel environment exploration behavior of great tits. Each SNP was entered into a separate model: a) SNP31, b) SNP100, c) SNP250, d) SNP438.

| 1. **SNP31** |  |  |  |  |  |
| --- | --- | --- | --- | --- | --- |
| **Fixed effects** | ***β* ± SE** | ***Z*** | ***P*** | **χ^2^_2_** | ***P*** |
| Intercept | 1.69 ± 0.192 | 8.85 | <0.001 |  |  |
| Date | -0.280 ± 0.105 | -2.66 | 0.001 |  |  |
| Test number | 0.301 ± 0.094 | 3.19 | 0.001 |  |  |
| SNP31 AT | -0.179 ± 0.262 | -0.684 | 0.494 |  |  |
| SNP31 TT | -0.050 ± 0.561 | -0.090 | 0.928 | 0.456 | 0.796 |
| Sex | 0.262 ± 0.253 | 1.03 | 0.300 |  |  |
| **Random effects** | **Variance** | **SD** | **N** |  |  |
| Observation | 0.886 | 0.942 | 203 |  |  |
| Individual | 1.04 | 1.02 | 140 |  |  |
| Study site | 0.015 | 0.124 | 5 |  |  |
| Year | <0.001 | <0.001 | 3 |  |  |
| 1. **SNP100** |  |  |  |  |  |
| **Fixed effects** | ***β* ± SE** | ***Z*** | ***P*** | **χ^2^_1_** | ***P*** |
| Intercept | 1.76 ± 0.187 | 9.41 | < 0.001 |  |  |
| Date | -0.416 ± 0.169 | -2.46 | 0.014 |  |  |
| Test number | 0.344 ± 0.108 | 3.18 | 0.001 |  |  |
| SNP100 AG/GG | -0.276 ± 0.314 | -0.879 | 0.379 | 0.780 | 0.377 |
| Sex | 0.286 ± 0.259 | 1.11 | 0.268 |  |  |
| **Random effects** | **Variance** | **SD** | **N** |  |  |
| Observation | 0.898 | 0.948 | 204 |  |  |
| Individual | 1.09 | 1.04 | 141 |  |  |
| Study site | 0.013 | 0.114 | 5 |  |  |
| Year | < 0.001 | < 0.001 | 3 |  |  |
| 1. **SNP250** |  |  |  |  |  |
| **Fixed effects** | ***β* ± SE** | ***Z*** | ***P*** | **χ^2^_2_** | ***P*** |
| Intercept | 1.95 ± 0.446 | 4.38 | <0.001 |  |  |
| Date | -0.471 ± 0.167 | -2.81 | 0.004 |  |  |
| Test number | 0.322 ± 0.108 | 2.99 | 0.002 |  |  |
| SNP31 AT | -0.421 ± 0.493 | -0.855 | 0.392 |  |  |
| SNP31 TT | -0.186 ± 0.462 | -0.404 | 0.686 | 1.08 | 0.582 |
| Sex | 0.298 ± 0.242 | 1.22 | 0.219 |  |  |
| **Random effects** | **Variance** | **SD** | **N** |  |  |
| Observation | 0.976 | 0.988 | 211 |  |  |
| Individual | 1.05 | 1.02 | 147 |  |  |
| Study site | <0.001 | <0.001 | 5 |  |  |
| Year | <0.001 | <0.001 | 3 |  |  |
| 1. **SNP438** |  |  |  |  |  |
| **Fixed effects** | ***β* ± SE** | **Z** | ***P*** | **χ^2^_2_** | ***P*** |
| Intercept | 1.58 ± 0.206 | 7.66 | <0.001 |  |  |
| Date | -0.449 ± 0.168 | -2.67 | 0.007 |  |  |
| Test number | 0.319 ± 0.108 | 2.94 | 0.003 |  |  |
| SNP438 CT | 0.466 ± 0.248 | 1.88 | 0.060 |  |  |
| SNP438 TT | -0.703 ± 0.551 | -1.27 | 0.201 | 6.12 | 0.047 |
| Sex | 0.266 ± 0.248 | 1.07 | 0.283 |  |  |
| **Random effects** | **Variance** | **SD** | **N** |  |  |
| Observation | 0.982 | 0.991 | 211 |  |  |
| Individual | 1.05 | 1.02 | 147 |  |  |
| Study site | 0.013 | 0.117 | 5 |  |  |
| Year | <0.001 | <0.001 | 3 |  |  |

**Reference genotypes are AA for SNP31, SNP100, SNP250 and CC for SNP438.*

**Table S6**. General generic GLMMs with Poisson distribution for relationships between eight exonic SNPs and the novel environment exploration behavior of great tits. Each SNP was entered into a separate model: a) SNP163 exon 1, b) SNP187 exon 1, c) SNP226 exon 1, d) SNP170 exon 3, e) SNP36 exon 6, f) SNP51 exon 9, g) SNP84 exon 9, h) SNP66 exon 13.

| 1. **SNP 163 exon 1** | |  |  |  |  |
| --- | --- | --- | --- | --- | --- |
| **Fixed effects** | ***β* ± SE** | ***Z*** | ***P*** | **χ^2^_2_** | ***P*** |
| Intercept | 1.42 ± 0.844 | 1.69 | 0.090 |  |  |
| Date | -0.522 ± 0.168 | -3.12 | 0.001 |  |  |
| Test number | 0.345 ± 0.105 | 3.27 | 0.001 |  |  |
| SNP163 AG | 0.191 ± 0.865 | 0.222 | 0.824 |  |  |
| SNP163 GG | 0.309 ± 0.844 | 0.367 | 0.713 | 0.299 | 0.861 |
| Sex | 0.277 ± 0.242 | 1.14 | 0.251 |  |  |
| **Random effects** | **Variance** | **SD** | **N** |  |  |
| Observation | 0.960 | 0.980 | 218 |  |  |
| Individual | 1.11 | 1.05 | 152 |  |  |
| Study site | <0.001 | <0.001 | 5 |  |  |
| Year | <0.001 | <0.001 | 3 |  |  |
| 1. **SNP226 exon 1** | |  |  |  |  |
| **Fixed effects** | ***β* ± SE** | ***Z*** | ***P*** | **χ^2^_2_** | ***P*** |
| Intercept | 1.776 ± 0.407 | 4.359 | < 0.001 |  |  |
| Date | -0.519 ± 0.164 | -3.153 | 0.001 |  |  |
| Test | 0.296 ± 0.092 | 3.207 | 0.001 |  |  |
| SNP226 AT | -0.329 ± 0.440 | -0.748 | 0.454 | 2.170 | 0.337 |
| SNP226 TT | 0.030 ± 0.428 | 0.072 | 0.942 |  |  |
| Sex | 0.283 ± 0.239 | 1.185 | 0.236 |  |  |
| **Random effects** | **Variance** | **SD** | **N** |  |  |
| Observation | 0.975 | 0.987 | 218 |  |  |
| Individual | 1.047 | 1.023 | 152 |  |  |
| Study site | < 0.001 | < 0.001 | 5 |  |  |
| Year | < 0.001 | < 0.001 | 3 |  |  |
| 1. **SNP187 exon 1** | |  |  |  |  |
| **Fixed effects** | ***β* ± SE** | ***Z*** | ***P*** | **χ^2^_2_** | ***P*** |
| Intercept | 1.728 ± 0.184 | 9.383 | < 0.001 |  |  |
| Date | -0.521 ± 0.165 | -3.163 | 0.001 |  |  |
| Test | 0.302 ± 0.092 | 3.268 | 0.001 |  |  |
| SNP187 TC | -0.231 ± 0.258 | -0.898 | 0.369 | 1.119 | 0.571 |
| SNP187 TT | 0.534 ± 1.081 | 0.494 | 0.621 |  |  |
| Sex | 0.279 ± 0.241 | 1.157 | 0.247 |  |  |
| **Random effects** | **Variance** | **SD** | **N** |  |  |
| Observation | 0.966 | 0.983 | 218 |  |  |
| Individual | 1.082 | 1.040 | 152 |  |  |
| Study site | <0.001 | <0.001 | 5 |  |  |
| Year | <0.001 | <0.001 | 3 |  |  |
| 1. **SNP170 exon 3** | |  |  |  |  |
| **Fixed effects** | ***β* ± SE** | ***Z*** | ***P*** | **χ^2^_1_** | ***P*** |
| Intercept | 1.68 ± 0.172 | 9.83 | < 0.001 |  |  |
| Date | -0.485 ± 0.162 | -3.00 | 0.002 |  |  |
| Test number | 0.339 ± 0.101 | 3.36 | < 0.001 |  |  |
| SNP170 CG | 0.338 ± 0.300 | 1.12 | 0.260 |  |  |
| SNP170 GG | 0.483 ± 0.782 | 0.618 | 0.536 | 1.56 | 0.458 |
| Sex | 0.200 ± 0.242 | 0.828 | 0.407 |  |  |
| **Random effects** | **Variance** | **SD** | **N** |  |  |
| Observation | 0.887 | 0.942 | 223 |  |  |
| Individual | 1.16 | 1.08 | 155 |  |  |
| Study site | <0.001 | <0.001 | 5 |  |  |
| Year | <0.001 | <0.001 | 3 |  |  |
| 1. **SNP36 exon 6** | |  |  |  |  |
| **Fixed effects** | ***β* ± SE** | ***Z*** | ***P*** | **χ^2^_2_** | ***P*** |
| Intercept | 1.93 ± 0.265 | 7.28 | <0.001 |  |  |
| Date | -0.484 ± 0.161 | -3.01 | 0.002 |  |  |
| Test number | 0.333 ± 0.101 | 3.31 | <0.001 |  |  |
| SNP36 CT | -0.065 ± 0.281 | -0.233 | 0.815 |  |  |
| SNP36 TT | -0.497 ± 0.322 | -1.54 | 0.122 | 2.96 | 0.228 |
| Sex | 0.190 ± 0.240 | 0.794 | 0.427 |  |  |
| **Random effects** | **Variance** | **SD** | **N** |  |  |
| Observation | 0.887 | 0.942 | 223 |  |  |
| Individual | 1.12 | 1.06 | 155 |  |  |
| Study site | <0.001 | <0.001 | 5 |  |  |
| Year | <0.001 | <0.001 | 3 |  |  |
| 1. **SNP51 exon 9** | |  |  |  |  |
| **Fixed effects** | ***β* ± SE** | ***Z*** | ***P*** | **χ^2^_2_** | ***P*** |
| Intercept | 1.77 ± 0.178 | 9.94 | < 0.001 |  |  |
| Date | -0.502 ± 0.162 | -3.10 | 0.001 |  |  |
| Test number | 0.346 ± 0.101 | 3.42 | <0.001 |  |  |
| SNP51 CT | 0.020 ± 0.279 | 0.074 | 0.941 |  |  |
| SNP51 TT | -0.485 ± 0.500 | -0.971 | 0.331 | 0.986 | 0.612 |
| Sex | 0.253 ± 0.238 | 1.06 | 0.286 |  |  |
| **Random effects** | **Variance** | **SD** | **N** |  |  |
| Observation | 0.907 | 0.953 | 223 |  |  |
| Individual | 1.12 | 1.06 | 155 |  |  |
| Study site | <0.001 | <0.001 | 5 |  |  |
| Year | <0.001 | <0.001 | 3 |  |  |
| 1. **SNP84 exon 9** | |  |  |  |  |
| **Fixed effects** | ***β* ± SE** | ***Z*** | ***P*** | **χ^2^_2_** | ***P*** |
| Intercept | 1.84 ± 0.267 | 6.91 | <0.001 |  |  |
| Date | -0.495 ± 0.161 | -3.06 | 0.002 |  |  |
| Test number | 0.342 ± 0.101 | 3.39 | <0.001 |  |  |
| SNP84 CT | -0.054 ± 0.297 | -0.182 | 0.855 |  |  |
| SNP84 TT | -0.222 ±0.327 | -0.679 | 0.497 | 0.552 | 0.759 |
| Sex | 0.237 ± 0.239 | 0.991 | 0.321 |  |  |
| **Random effects** | **Variance** | **SD** | **N** |  |  |
| Observation | 0.896 | 0.947 | 223 |  |  |
| Individual | 1.14 | 1.07 | 155 |  |  |
| Study site | <0.001 | <0.001 | 5 |  |  |
| Year | <0.001 | <0.001 | 3 |  |  |
| 1. **SNP66 exon 13** | |  |  |  |  |
| **Fixed effects** | ***β* ± SE** | ***Z*** | ***P*** | **χ^2^_2_** | ***P*** |
| Intercept | 1.60 ± 0.198 | 8.11 | <0.001 |  |  |
| Date | -0.463 ± 0.166 | -2.80 | 0.005 |  |  |
| Test number | 0.321 ± 0.104 | 3.11 | 0.001 |  |  |
| SNP66 CT | 0.356 ± 0.257 | 1.39 | 0.164 |  |  |
| SNP66 TT | 0.176 ± 0.492 | 0.359 | 0.719 | 1.92 | 0.383 |
| Sex | 0.212 ± 0.251 | 0.847 | 0.397 |  |  |
| **Random effects** | **Variance** | **SD** | **N** |  |  |
| Observation | 0.923 | 0.961 | 205 |  |  |
| Individual | 1.08 | 1.04 | 140 |  |  |
| Study site | <0.001 | <0.001 | 5 |  |  |
| Year | <0.001 | <0.001 | 3 |  |  |

**Reference genotypes are AA for SNP163 exon 1, SNP226 exon 1 and SNP170 exon 3, and CC for SNP187 exon 1, SNP36 exon 6, SNP51 exon 9, SNP84 exon 9 and SNP66 exon 13.*

**Table S7.** Poisson GLMMs for the effect of SNPs in the promoter region on incorrect attempts on the barrier during obstacle removal problem-solving tests in females, with time on the nest box as an offset variable; a) SNP31, b) SNP100, c) SNP250, and d) SNP438. General generic models are shown.

| 1. **SNP31** |  |  |  |  |  |
| --- | --- | --- | --- | --- | --- |
| **Fixed Effects** | ***β* ± SE** | ***Z*** | ***P*** |  |  |
| Intercept | 2.677 ± 0.882 | 3.035 | 0.002 |  |  |
| SNP31 AT^a^ | -0.245 ± 0.644 | -0.380 | 0.704 |  |  |
| Trial number | -1.715 ± 0.672 | -2.551 | 0.010 |  |  |
| **Random effects** | **Variance** | **SD** | **N** |  |  |
| Observation | 2.951 | 1.718 | 64 |  |  |
| Individual | 0.989 | 0.994 | 44 |  |  |
| Study site | <0.001 | <0.001 | 3 |  |  |
| 1. **SNP100** | |  |  |  |  |
| **Fixed Effects** | ***β* ± SE** | ***Z*** | ***P*** |  |  |
| Intercept | 2.128 ± 0.926 | 2.299 | 0.021 |  |  |
| SNP100 AG^b^ | 0.779 ± 0.844 | 0.922 | 0.356 |  |  |
| Trial number | -1.704 ± 0.692 | -2.462 | 0.014 |  |  |
| **Random effects** | **Variance** | **SD** | **N** |  |  |
| Observation | 3.117 | 1.766 | 65 |  |  |
| Individual | 1.529 | 1.237 | 45 |  |  |
| Study site | <0.001 | <0.001 | 3 |  |  |
| 1. **SNP250** | |  |  |  |  |
| **Fixed Effects** | ***β* ± SE** | ***Z*** | ***P*** | ***χ^2^_2_*** | ***P*** |
| Intercept | 2.896 ± 2.045 | 1.416 | 0.156 |  |  |
| SNP250 AG | -0.036 ± 1.965 | -0.018 | 0.985 |  |  |
| SNP250 GG | -0.531 ± 1.912 | -0.278 | 0.781 | 0.489 | 0.783 |
| Trial number | -1.791 ± 0.684 | -2.619 | 0.008 |  |  |
| **Random effects** | **Variance** | **SD** | **N** |  |  |
| Observation | 3.009 | 1.735 | 69 |  |  |
| Individual | 1.424 | 1.193 | 48 |  |  |
| Study site | <0.001 | <0.001 | 3 |  |  |
| 1. **SNP438** |  |  |  |  |  |
| **Fixed Effects** | ***β* ± SE** | ***Z*** | ***P*** | ***χ^2^_2_*** | ***P*** |
| Intercept | 2.925 ± 0.943 | 3.099 | 0.002 |  |  |
| SNP438 TC | -0.611 ± 0.655 | -0.932 | 0.352 |  |  |
| SNP438 TT | -1.688 ± 1.495 | -1.129 | 0.259 | 1.782 | 0.410 |
| Trial number | -1.818 ± 0.696 | -2.609 | 0.009 |  |  |
| **Random effects** | **Variance** | **SD** | **N** |  |  |
| Observation | 3.322 | 1.823 | 69 |  |  |
| Individual | 1.029 | 1.014 | 48 |  |  |
| Study site | <0.001 | <0.001 | 3 |  |  |

**Reference genotypes are AA for SNP31, SNP100, SNP250 and CC for SNP438.*

*^a^Three individuals with genotype TT dropped to facilitate model convergence.*

*^b^One individual with genotype GG dropped to facilitate model convergence.*

**Table S8.** Poisson GLMMs for the effect of exonic SNPs on incorrect attempts on the barrier in females, with time on the nest box as an offset variable; a) SNP163 exon 1, b) SNP170 exon 3, c) SNP51 exon 9, e) SNP84 exon 9, and f) SNP66 exon 13. General generic models are shown.

| 1. **SNP163 exon 1** |  |  |  |  |  |
| --- | --- | --- | --- | --- | --- |
| **Fixed Effects** | ***β* ± SE** | ***Z*** | ***P*** |  |  |
| Intercept | 2.284 ± 1.169 | 1.954 | 0.051 |  |  |
| SNP163 GG | 0.259 ± 0.908 | 0.285 | 0.776 |  |  |
| Trial number | -1.748 ± 0.660 | -2.651 | 0.008 |  |  |
| **Random effects** | **Variance** | **SD** | ***N*** |  |  |
| Observation | 2.890 | 1.699 | 73 |  |  |
| Individual | 1.146 | 1.070 | 52 |  |  |
| Study site | < 0.001 | < 0.001 | 3 |  |  |
| 1. **SNP170 exon 3** |  |  |  |  |  |
| **Fixed Effects** | ***β* ± SE** | ***Z*** | ***P*** | ***χ^2^_2_*** | ***P*** |
| Intercept | 2.676 ± 0.847 | 3.158 | 0.002 |  |  |
| SNP170 CG | -0.484 ± 0.838 | -0.578 | 0.563 |  |  |
| SNP170 GG | -1.334 ± 1.409 | -0.947 | 0.343 | 1.171 | 0.556 |
| Trial number | -1.766 ± 0.664 | 2.662 | 0.008 |  |  |
| **Random effects** | **Variance** | **SD** | **N** |  |  |
| Observation | 2.925 | 1.710 | 73 |  |  |
| Individual | 1.104 | 1.051 | 52 |  |  |
| Study site | < 0.001 | < 0.001 | 3 |  |  |
| 1. **SNP51 exon 9** |  |  |  |  |  |
| **Fixed Effects** | ***β* ± SE** | ***Z*** | ***P*** | ***χ^2^_2_*** | ***P*** |
| Intercept | 2.355 ± 0.856 | 2.750 | 0.005 |  |  |
| SNP51 CT | 0.717 ± 0.692 | 1.036 | 0.300 |  |  |
| SNP51 TT | -0.209 ± 1.354 | -0.155 | 0.877 | 1.165 | 0.558 |
| Trial number | -1.759 ± 0.660 | -2.663 | 0.007 |  |  |
| **Random effects** | **Variance** | **SD** | **N** |  |  |
| Observation | 2.944 | 1.716 | 73 |  |  |
| Individual | 1.052 | 1.026 | 52 |  |  |
| Study site | 0 | 0 | 3 |  |  |
| 1. **SNP84 exon 9** |  |  |  |  |  |
| **Fixed Effects** | ***β* ± SE** | ***Z*** | ***P*** | ***χ^2^_2_*** | ***P*** |
| Intercept | 2.636 ± 1.105 | 2.596 | 0.009 |  |  |
| SNP84 CT | -0.024 ± 0.720 | -0.035 | 0.972 |  |  |
| SNP84 TT | -0.722 ± 0.896 | -0.807 | 0.419 | 0.872 | 0.648 |
| Trial number | -1.716 ± 0.660 | -2.598 | 0.009 |  |  |
| **Random effects** | **Variance** | **SD** | **N** |  |  |
| Observation | 2.732 | 1.653 | 73 |  |  |
| Individual | 1.301 | 1.140 | 53 |  |  |
| Study site | < 0.001 | < 0.001 | 3 |  |  |
| 1. **SNP66 exon 13** |  |  |  |  |  |
| **Fixed Effects** | ***β* ± SE** | ***Z*** | ***P*** | ***χ^2^_2_*** | ***P*** |
| Intercept | 2.224 ± 0.847 | 2.626 | 0.008 |  |  |
| SNP66 CT | -0.361 ± 0.645 | -0.559 | 0.576 |  |  |
| SNP66 TT | -0.178 ± 1.462 | -0.122 | 0.902 | 0.315 | 0.854 |
| Trial number | -1.231 ± 0.647 | -1.900 | 0.057 |  |  |
| **Random effects** | **Variance** | **SD** | **N** |  |  |
| Observation | 2.417 | 1.555 | 64 |  |  |
| Individual | 1.078 | 1.038 | 47 |  |  |
| Study site | <0.001 | <0.001 | 3 |  |  |

**Reference genotypes are AA for SNP163 exon 1and SNP170 exon 3, and CC for SNP36 exon 6, SNP51 exon 9, SNP84 exon 9 and SNP66 exon 13.*

**Table S9.** Poisson GLMMs for the effect of SNPs in the promoter region on incorrect attempts on the barrier during obstacle removal problem-solving tests in males, with time on the nest box as an offset variable; a) SNP31, b) SNP100, c) SNP250, and d) SNP438. General generic models are shown.

| 1. **SNP31** |  |  |  |  |  |
| --- | --- | --- | --- | --- | --- |
| **Fixed Effects** | ***β* ± SE** | ***Z*** | ***P*** | ***χ^2^_2_*** | ***P*** |
| Intercept | -0.966 ± 0.704 | -1.373 | 0.169 |  |  |
| SNP31 AT | -3.411 ± 1.634 | -2.088 | 0.037^a^ |  |  |
| SNP31 TT | 0.175 ± 1.678 | 0.105 | 0.916 | 5.50 | 0.064 |
| **Random effects** | **Variance** | **SD** | **N** |  |  |
| Observation | 2.553 | 1.598 | 54 |  |  |
| Individual | 2.972 | 1.724 | 36 |  |  |
| Study site | <0.001 | <0.001 | 3 |  |  |
| 1. **SNP100** |  |  |  |  |  |
| **Fixed Effects** | ***β* ± SE** | ***Z*** | ***P*** |  |  |
| Intercept | -1.837 ± 0.858 | -2.138 | 0.032 |  |  |
| SNP100 AG^b^ | 0.049 ± 1.278 | 0.038 | 0.970 |  |  |
| **Random effects** | **Variance** | **SD** | **N** |  |  |
| Observation | 2.786 | 1.669 | 54 |  |  |
| Individual | 4.689 | 2.165 | 36 |  |  |
| Study site | <0.001 | <0.001 | 3 |  |  |
| 1. **SNP250** |  |  |  |  |  |
| **Fixed Effects** | ***β* ± SE** | ***Z*** | ***P*** | ***χ^2^_2_*** | ***P*** |
| Intercept | -0.102 ± 1.966 | -0.052 | 0.959 |  |  |
| SNP250 AG | -1.660 ± 2.224 | -0.747 | 0.455 |  |  |
| SNP250 GG | -1.620 ± 2.127 | -0.762 | 0.446 | 0.638 | 0.727 |
| **Random effects** | **Variance** | **SD** | **N** |  |  |
| Observation | 2.649 | 1.628 | 58 |  |  |
| Individual | 4.699 | 2.168 | 39 |  |  |
| Study site | <0.001 | <0.001 | 3 |  |  |
| 1. **SNP438** |  |  |  |  |  |
| **Fixed Effects** | ***β* ± SE** | ***Z*** | ***P*** | ***χ^2^_2_*** | ***P*** |
| Intercept | -1.287 ± 0.733 | -1.754 | 0.079 |  |  |
| SNP438 TC | -1.989 ± 1.441 | -1.381 | 0.167 |  |  |
| SNP438 TT | 1.931 ± 2.579 | 0.749 | 0.454 | 1.782 | 0.410 |
| **Random effects** | **Variance** | **SD** | **N** |  |  |
| Observation | 2.420 | 1.556 | 58 |  |  |
| Individual | 4.844 | 2.201 | 39 |  |  |
| Study site | <0.001 | <0.001 | 3 |  |  |

**Reference genotypes are AA for SNP31, SNP100, SNP250 and CC for SNP438.*

*^a^P = 0.092 after adjusting for multiple comparisons.*

*^b^No male had genotype GG.*

**Table S10.** Poisson GLMMs for the effect of exonic SNPs on incorrect attempts on the barrier in males, with time on the nest box as an offset variable; a) SNP163 exon 1, b) SNP187 exon 1, c) SNP226 exon 1, d) SNP170 exon 3, e) SNP36 exon 6, f) SNP51 exon 9, g) SNP84 exon 9, and h) SNP66 exon 13. General generic models are shown.

| 1. **SNP163 exon 1** |  |  |  |  |  |
| --- | --- | --- | --- | --- | --- |
| **Fixed Effects** | ***β* ± SE** | ***Z*** | ***P*** |  |  |
| Intercept | -2.341 ± 1.193 | -1.961 | 0.049 |  |  |
| SNP163 GG | 0.960 ± 1.185 | 0.810 | 0.418 |  |  |
| **Random effects** | **Variance** | **SD** | **N** |  |  |
| Observation | 2.666 | 1.633 | 59 |  |  |
| Individual | 4.546 | 2.132 | 40 |  |  |
| Study site | < 0.001 | < 0.001 | 3 |  |  |
| 1. **SNP187 exon 1** |  |  |  |  |  |
| **Fixed Effects** | ***β* ± SE** | ***Z*** | ***P*** |  |  |
| Intercept | -1.949 ± 0.827 | -2.355 | 0.018 |  |  |
| SNP187 TC | 1.039 ± 1.081 | 0.961 | 0.336 |  |  |
| **Random effects** | **Variance** | **SD** | **N** |  |  |
| Observation | 2.616 | 1.617 | 59 |  |  |
| Individual | 4.402 | 2.098 | 40 |  |  |
| Study site | <0.001 | <0.001 | 3 |  |  |
| 1. **SNP226 exon 1** |  |  |  |  |  |
| **Fixed Effects** | ***β* ± SE** | ***Z*** | ***P*** | ***χ^2^_2_*** | ***P*** |
| Intercept | 1.000 ± 2.038 | 0.491 | 0.624 |  |  |
| SNP226 AT | -2.266 ± 2.149 | -1.055 | 0.292 |  |  |
| SNP226 TT | -2.886 ± 2.113 | -1.366 | 0.172 | 1.851 | 0.396 |
| **Random effects** | **Variance** | **SD** | **N** |  |  |
| Observation | 2.504 | 1.582 | 59 |  |  |
| Individual | 3.779 | 2.120 | 40 |  |  |
| Study site | <0.001 | <0.001 | 3 |  |  |
| 1. **SNP170 exon 3** |  |  |  |  |  |
| **Fixed Effects** | ***β* ± SE** | ***Z*** | ***P*** | ***χ^2^_2_*** | ***P*** |
| Intercept | -1.964 ± 0.868 | -2.261 | 0.023 |  |  |
| SNP170 CG | 0.442 ± 1.189 | 0.372 | 0.709 | 0.969 | 0.615 |
| SNP170 GG | 2.710 ± 2.921 | 0.928 | 0.353 |  |  |
| **Random effects** | **Variance** | **SD** | **N** |  |  |
| Observation | 2.814 | 1.677 | 60 |  |  |
| Individual | 4.750 | 2.179 | 41 |  |  |
| Study site | <0.001 | <0.001 | 3 |  |  |
| 1. **SNP36 exon 6** |  |  |  |  |  |
| **Fixed Effects** | ***β* ± SE** | ***Z*** | ***P*** | ***χ^2^_2_*** | ***P*** |
| Intercept | -1.133 ± 0.959 | -1.181 | 0.238 |  |  |
| SNP36 CT | -0.655 ± 1.289 | -0.508 | 0.611 |  |  |
| SNP36 TT | -1.230 ± 1.321 | -0.931 | 0.352 | 0.904 | 0.637 |
| **Random effects** | **Variance** | **SD** | **N** |  |  |
| Observation | 2.815 | 1.678 | 60 |  |  |
| Individual | 4.729 | 2.175 | 41 |  |  |
| Study site | < 0.001 | < 0.001 | 3 |  |  |
| 1. **SNP51 exon 9** |  |  |  |  |  |
| **Fixed Effects** | ***β* ± SE** | ***Z*** | ***P*** | ***χ^2^_2_*** | ***P*** |
| Intercept | -1.666 ± 0.806 | -2.069 | 0.039 |  |  |
| SNP51 CT^a^ | -0.060 ± 1.198 | -0.050 | 0.960 |  |  |
| **Random effects** | **Variance** | **SD** | **N** |  |  |
| Observation | 2.710 | 1.646 | 58 |  |  |
| Individual | 4.654 | 2.157 | 40 |  |  |
| Study site | < 0.001 | < 0.001 | 3 |  |  |
| 1. **SNP84 exon 9** |  |  |  |  |  |
| **Fixed Effects** | ***β* ± SE** | ***Z*** | ***P*** | ***χ^2^_2_*** | ***P*** |
| Intercept | -0.596 ± 1.257 | -0.474 | 0.635 |  |  |
| SNP84 CT | -0.945 ± 1.489 | -0.635 | 0.525 |  |  |
| SNP84 TT | -1.918 ± 1.571 | -1.221 | 0.222 | 1.726 | 0.422 |
| **Random effects** | **Variance** | **SD** | **N** |  |  |
| Observation | 2.746 | 1.6576 | 60 |  |  |
| Individual | 4.645 | 2.155 | 41 |  |  |
| Study site | <0.001 | <0.001 | 3 |  |  |
| 1. **SNP66 exon 13** |  |  |  |  |  |
| **Fixed Effects** | ***β* ± SE** | ***Z*** | ***P*** | ***χ^2^_2_*** | ***P*** |
| Intercept | -1.972 ± 0.871 | -2.265 | 0.023 |  |  |
| SNP66 CT | 0.116 ± 1.065 | 0.109 | 0.913 |  |  |
| SNP66 TT | -1.281 ± 1.952 | -0.656 | 0.511 | 0.506 | 0.776 |
| **Random effects** | **Variance** | **SD** | **N** |  |  |
| Observation | 3.907 | 1.977 | 53 |  |  |
| Individual | 2.085 | 1.444 | 36 |  |  |
| Study site | <0.001 | <0.001 | 3 |  |  |

**Reference genotypes are AA for SNP163 exon 1and SNP170 exon 3, and CC for SNP36 exon 6, SNP51 exon 9, SNP84 exon 9 and SNP66 exon 13.*

*^a^Two individuals with genotype TT dropped to facilitate model convergence.*

**Table S11**. General generic LMMs for the relationship between SNPs in the promoter region and latency of female great tits to contact the test apparatus during obstacle removal problem-solving tests; a) SNP31, b) SNP100, c) SNP250, d) SNP438.

| 1. **SNP31** | |  |  |  |  |  |  |
| --- | --- | --- | --- | --- | --- | --- | --- |
| **Fixed effects** | ***β* ± SE** | ***d.f.*** | ***t*** | ***P*** | ***F*** | ***d.f.*** | ***P*** |
| Intercept | 2.427 ± 0.283 | 2.591 | 8.555 | 0.005 |  |  |  |
| SNP31 AT | 0.181 ± 0.299 | 39.9 | 0.609 | 0.546 |  |  |  |
| SNP31 TT | 0.819 ± 0.562 | 45.2 | 1.458 | 0.151 | 1.093 | 2,43 | 0.344 |
| **Random effects** | **Variance** | **SD** | **N** |  |  |  |  |
| Individual | 0.209 | 0.457 | 47 |  |  |  |  |
| Study site | 0.148 | 0.385 | 3 |  |  |  |  |
| Residual | 0.771 | 0.878 | 68 |  |  |  |  |
| 1. **SNP100** | |  |  |  |  |  |  |
| **Fixed effects** | ***β* ± SE** | ***d.f*** | ***t*** | ***P*** | ***F*** | ***d.f.*** | ***P*** |
| Intercept | 2.439 ± 0.153 | 1.657 | 15.9 | 0.008 |  |  |  |
| SNP100 AG | 0.302 ± 0.328 | 13.1 | 0.922 | 0.373 |  |  |  |
| SNP100 GG | 1.281 ± 0.784 | 22.0 | 1.635 | 0.16 | 1.629 | 2, 18 | 0.224 |
| **Random effects** | **Variance** | **SD** | **N** |  |  |  |  |
| Individual | 0.161 | 0.402 | 67 |  |  |  |  |
| Study site | 0.005 | 0.074 | 3 |  |  |  |  |
| Residual | 0.851 | 0.922 | 46 |  |  |  |  |
| 1. **SNP250** | |  |  |  |  |  |  |
| **Fixed effects** | ***β* ± SE** | ***d.f*** | ***t*** | ***P*** | ***F*** | ***d.f.*** | ***P*** |
| Intercept | 2.321 ± 0.828 | 24.5 | 2.801 | 0.009 |  |  |  |
| SNP250 AG | 0.257 ± 0.845 | 25.5 | 0.304 | 0.764 |  |  |  |
| SNP250 GG | 0.176 ± 0.841 | 25.7 | 0.210 | 0.835 | 0.067 | 2,27 | 0.935 |
| **Random effects** | **Variance** | **SD** | **N** |  |  |  |  |
| Individual | 0.237 | 0.487 | 48 |  |  |  |  |
| Study site | 0.043 | 0.208 | 3 |  |  |  |  |
| Residual | 0.834 | 0.913 | 69 |  |  |  |  |
| 1. **SNP438** | |  |  |  |  |  |  |
| **Fixed effects** | ***β* ± SE** | ***d.f*** | ***t*** | ***P*** | ***F*** | ***d.f.*** | ***P*** |
| Intercept | 2.673 ± 0.194 | 4.513 | 13.7 | <0.001 |  |  |  |
| SNP438 TC | -0.230 ± 0.274 | 34.6 | -0.840 | 0.407 |  |  |  |
| SNP438 TT | -0.809 ± 0.577 | 43.9 | -1.402 | 0.168 | 1.120 | 2,41 | 0.336 |
| **Random effects** | **Variance** | **SD** | **N** |  |  |  |  |
| Individual | 0.257 | 0.507 | 48 |  |  |  |  |
| Study site | 0.004 | 0.064 | 3 |  |  |  |  |
| Residual | 0.807 | 0.898 | 69 |  |  |  |  |

**Reference genotypes are AA for SNP31, SNP100, SNP250 and CC for SNP438.*

**Table S12**. General generic LMMs for relationships between exonic SNPs and latency of female great tits to contact the test apparatus during obstacle removal problem-solving tests; a) SNP163 exon 1, b) SNP187 exon 1, c) SNP226 exon 1, d) SNP170 exon 3, e) SNP36 exon 6, f) SNP51 exon 9, g) SNP84 exon9, h) SNP66 exon 13.

| 1. **SNP163 exon 1** | |  |  |  |  |  |  |
| --- | --- | --- | --- | --- | --- | --- | --- |
| **Fixed effects** | ***β* ± SE** | ***d.f.*** | ***t*** | ***P*** |  |  |  |
| Intercept | 2.286 ± 0.419 | 23.9 | 5.451 | <0.001 |  |  |  |
| SNP163 GG | 0.266 ± 0.427 | 36.4 | 0.624 | 0.537 |  |  |  |
| **Random effects** | **Variance** | **SD** | **N** |  |  |  |  |
| Individual | 0.227 | 0.476 | 73 |  |  |  |  |
| Study site | 0.049 | 0.221 | 3 |  |  |  |  |
| Residual | 0.855 | 0.925 | 52 |  |  |  |  |
| 1. **SNP187 exon 1** | |  |  |  |  |  |  |
| **Fixed effects** | ***β* ± SE** | ***d.f.*** | ***t*** | ***P*** | ***F*** | ***d.f.*** | ***P*** |
| Intercept | 2.450 ± 0.217 | 2.956 | 11.3 | 0.001 |  |  |  |
| SNP187 TC | 0.189 ± 0.296 | 43.9 | 0.641 | 0.524 |  |  |  |
| SNP187 TT | 0.243 ± 0.542 | 34.3 | 0.449 | 0.656 | 0.261 | 2,38 | 0.771 |
| **Random effects** | **Variance** | **SD** | **N** |  |  |  |  |
| Individual | 0.255 | 0.505 | 52 |  |  |  |  |
| Study site | 0.060 | 0.245 | 3 |  |  |  |  |
| Residual | 0.842 | 0.917 | 73 |  |  |  |  |
| 1. **SNP226 exon 1** | |  |  |  |  |  |  |
| **Fixed effects** | ***β* ± SE** | ***d.f.*** | ***t*** | ***P*** | ***F*** | ***d.f.*** | ***P*** |
| Intercept | 2.301 ± 0.318 | 19.2 | 7.222 | <0.001 |  |  |  |
| SNP226 AT | 0.040 ± 0.394 | 38.3 | 0.102 | 0.919 |  |  |  |
| SNP226 TT | 0.379 ± 0.358 | 34.2 | 1.056 | 0.298 | 0.928 | 2,39 | 0.404 |
| **Random effects** | **Variance** | **SD** | **N** |  |  |  |  |
| Individual | 0.223 | 0.472 | 52 |  |  |  |  |
| Study site | 0.011 | 0.109 | 3 |  |  |  |  |
| Residual | 0.867 | 0.931 | 73 |  |  |  |  |
| 1. **SNP170 exon 3** | |  |  |  |  |  |  |
| **Fixed effects** | ***β* ± SE** | ***d.f.*** | ***t*** | ***P*** | ***F*** | ***d.f.*** | ***P*** |
| (Intercept) | 2.599 ± 0.154 | 2.491 | 16.8 | <0.001 |  |  |  |
| SNP170 CG | -0.417 ± 0.374 | 35.1 | -1.115 | 0.272 |  |  |  |
| SNP170 GG | -0.346 ± 0.574 | 45.9 | -0.603 | 0.549 | 0.741 | 2,40 | 0.483 |
| **Random effects** | **Variance** | **SD** | **N** |  |  |  |  |
| Individual | 0.264 | 0.514 | 52 |  |  |  |  |
| Study site | 0.007 | 0.083 | 3 |  |  |  |  |
| Residual | 0.842 | 0.917 | 73 |  |  |  |  |
| 1. **SNP36 exon 6** | |  |  |  |  |  |  |
| **Fixed effects** | ***β* ± SE** | ***d.f*** | ***t*** | ***P*** | ***F*** | ***d.f.*** | ***P*** |
| Intercept | 2.866 ± 0.341 | 17.5 | 8.415 | <0.001 |  |  |  |
| SNP36 CT | -0.523 ± 0.357 | 44.4 | -1.467 | 0.149 |  |  |  |
| SNP36 TT | -0.224 ± 0.393 | 44.4 | -0.569 | 0.569 | 1.264 | 2,44 | 0.293 |
| **Random effects** | **Variance** | **SD** | **N** |  |  |  |  |
| Individual | 0.219 | 0.468 | 52 |  |  |  |  |
| Study site | 0.053 | 0.231 | 3 |  |  |  |  |
| Residual | 0.844 | 0.919 | 73 |  |  |  |  |
| 1. **SNP51 exon 9** | |  |  |  |  |  |  |
| **Fixed effects** | ***β* ± SE** | ***d.f*** | ***t*** | ***P*** | ***F*** | ***d.f.*** | ***P*** |
| Intercept | 2.621 ± 0.194 | 2.910 | 13.5 | 0.001 |  |  |  |
| SNP51 CT | -0.377 ± 0.325 | 48.6 | -1.164 | 0.250 |  |  |  |
| SNP51 TT | -0.315 ± 0.604 | 27.3 | -0.523 | 0.605 | 0.747 | 2,35 | 0.481 |
| **Random effects** | **Variance** | **SD** | **N** |  |  |  |  |
| Individual | 0.256 | 0.506 | 52 |  |  |  |  |
| Study site | 0.042 | 0.205 | 3 |  |  |  |  |
| Residual | 0.830 | 0.911 | 73 |  |  |  |  |
| 1. **SNP84 exon 9** | |  |  |  |  |  |  |
| **Fixed effects** | ***β* ± SE** | ***d.f*** | ***t*** | ***P*** | ***F*** | ***d.f.*** | ***P*** |
| Intercept | 2.547 ± 0.276 | 11.0 | 9.234 | <0.001 |  |  |  |
| SNP84 CT | 0.070 ± 0.314 | 31.3 | 0.223 | 0.825 |  |  |  |
| SNP84 TT | -0.256 ± 0.366 | 33.3 | -0.702 | 0.487 | 0.538 | 2,36 | 0.588 |
| **Random effects** | **Variance** | **SD** | **N** |  |  |  |  |
| Individual | 0.174 | 0.418 | 52 |  |  |  |  |
| Study site | 0.031 | 0.177 | 3 |  |  |  |  |
| Residual | 0.911 | 0.955 | 73 |  |  |  |  |
| 1. **SNP66 exon 13** | |  |  |  |  |  |  |
| **Fixed effects** | ***β* ± SE** | ***d.f*** | ***t*** | ***P*** | ***F*** | ***d.f.*** | ***P*** |
| Intercept | 2.521 ± 0.231 | 3.083 | 10.9 | 0.001 |  |  |  |
| SNP66 CT | 0.097 ± 0.317 | 36.5 | 0.306 | 0.761 |  |  |  |
| SNP66 TT | -0.466 ± 0.543 | 43.7 | 0.858 | 0.395 | 0.375 | 2,40 | 0.690 |
| **Random effects** | **Variance** | **SD** | **N** |  |  |  |  |
| Individual | 0.184 | 0.429 | 47 |  |  |  |  |
| Study site | 0.054 | 0.233 | 3 |  |  |  |  |
| Residual | 0.985 | 0.993 | 64 |  |  |  |  |

**Reference genotypes are AA for SNP163 exon 1and SNP170 exon 3, and CC for SNP36 exon 6, SNP51 exon 9, SNP84 exon 9 and SNP66 exon 13.*

**Table S13**. General generic LMMs for the relationship between SNPs in the promoter region and latency of male great tits to contact the test apparatus during obstacle removal problem-solving tests; a) SNP31, b) SNP100, c) SNP250, d) SNP438.

| 1. **SNP31** | |  |  |  |  |  |  |
| --- | --- | --- | --- | --- | --- | --- | --- |
| **Fixed effects** | ***β* ± SE** | ***d.f*** | ***t*** | ***P*** | ***F*** | ***d.f.*** | ***P*** |
| Intercept | 2.475 ± 0.355 | 2.504 | 6.956 | 0.010 |  |  |  |
| SNP31 AT | -0.027 ± 0.355 | 50.7 | -0.076 | 0.939 |  |  |  |
| SNP31 TT | 0.938 ± 0.593 | 50.0 | 1.580 | 0.121 | 1.329 | 2,50 | 0.274 |
| **Random effects** | **Variance** | **SD** | **N** |  |  |  |  |
| Individual | <0.001 | <0.001 | 36 |  |  |  |  |
| Study site | 0.271 | 0.520 | 3 |  |  |  |  |
| Residual | 1.216 | 1.103 | 54 |  |  |  |  |
| 1. **SNP100** | |  |  |  |  |  |  |
| **Fixed effects** | ***β* ± SE** | ***d.f*** | ***t*** | ***P*** | ***F*** | ***d.f.*** | ***P*** |
| Intercept | 2.404 ± 0.331 | 2.343 | 7.273 | 0.011 |  |  |  |
| SNP100 AG^a^ | 0.538 ± 0.355 | 34.9 | 1.514 | 0.139 |  |  |  |
| **Random effects** | **Variance** | **SD** | **N** |  |  |  |  |
| Individual | 0.043 | 0.208 | 36 |  |  |  |  |
| Study site | 0.237 | 0.487 | 3 |  |  |  |  |
| Residual | 1.166 | 1.080 | 54 |  |  |  |  |
| 1. **SNP250** | |  |  |  |  |  |  |
| **Fixed effects** | ***β* ± SE** | ***d.f*** | ***t*** | ***P*** | ***F*** | ***d.f.*** | ***P*** |
| Intercept | 2.594 ± 0.741 | 30.1 | 3.502 | 0.001 |  |  |  |
| SNP250 AG | -0.081 ± 0.732 | 54.9 | -0.111 | 0.912 |  |  |  |
| SNP250 GG | -0.095 ± 0.723 | 54.9 | -0.132 | 0.895 | 0.008 | 2,54 | 0.991 |
| **Random effects** | **Variance** | **SD** | **N** |  |  |  |  |
| Individual | <0.001 | <0.001 | 39 |  |  |  |  |
| Study site | 0.232 | 0.482 | 3 |  |  |  |  |
| Residual | 1.293 | 1.137 | 58 |  |  |  |  |
| 1. **SNP438** | |  |  |  |  |  |  |
| **Fixed effects** | ***β* ± SE** | ***d.f*** | ***t*** | ***P*** | ***F*** | ***d.f.*** | ***P*** |
| Intercept | 2.587 ± 0.361 | 2.351 | 7.175 | 0.012 |  |  |  |
| SNP438 TC | -0.381 ± 0.323 | 53.7 | -1.180 | 0.243 |  |  |  |
| SNP438 TT | 1.129 ± 0.816 | 54.0 | 1.384 | 0.172 | 1.910 | 2,54 | 0.158 |
| **Random effects** | **Variance** | **SD** | **N** |  |  |  |  |
| Individual | <0.001 | <0.001 | 39 |  |  |  |  |
| Study site | 0.297 | 0.545 | 3 |  |  |  |  |
| Residual | 1.199 | 1.095 | 58 |  |  |  |  |

**Reference genotypes are AA for SNP31, SNP100, SNP250 and CC for SNP438.*

*^a^No male had genotype GG*.

**Table S14**. General generic LMMs for the relationship between exonic SNPs and latency of male great tits to contact the test apparatus during obstacle removal problem-solving tests; a) SNP163 exon 1, b) SNP187 exon 1, c) SNP226 exon 1, d) SNP170 exon 3, e) SNP36 exon 6, f) SNP51 exon 9, g) SNP84 exon9, h) SNP66 exon 13.

| 1. **SNP163 exon 1** | |  |  |  |  |  |  |
| --- | --- | --- | --- | --- | --- | --- | --- |
| **Fixed effects** | ***β* ± SE** | ***d.f.*** | ***t*** | ***P*** |  |  |  |
| Intercept | 2.661 ± 0.355 | 3.940 | 7.489 | 0.001 |  |  |  |
| SNP163 GG | -0.258 ± 0.302 | 55.4 | -0.857 | 0.395 |  |  |  |
| **Random effects** | **Variance** | **SD** | **N** |  |  |  |  |
| Individual | <0.001 | <0.001 | 40 |  |  |  |  |
| Study site | 0.208 | 0.456 | 3 |  |  |  |  |
| Residual | 1.240 | 1.114 | 59 |  |  |  |  |
| 1. **SNP187 exon 1** | |  |  |  |  |  |  |
| **Fixed effects** | ***β* ± SE** | ***d.f.*** | ***t*** | ***P*** |  |  |  |
| Intercept | 2.532 ± 0.320 | 2.472 | 7.918 | 0.008 |  |  |  |
| SNP187 TC^a^ | -0.109 ± 0.318 | 55.3 | -0.345 | 0.732 |  |  |  |
| **Random effects** | **Variance** | **SD** | **N** |  |  |  |  |
| Individual | <0.001 | <0.001 | 40 |  |  |  |  |
| Study site | 0.215 | 0.463 | 4 |  |  |  |  |
| Residual | 1.254 | 1.119 | 59 |  |  |  |  |
| 1. **SNP226 exon 1** | |  |  |  |  |  |  |
| **Fixed effects** | ***β* ± SE** | ***d.f.*** | ***t*** | ***P*** | ***F*** | ***d.f.*** | ***P*** |
| Intercept | 2.204 ± 0.841 | 44.4 | 2.622 | 0.011 |  |  |  |
| SNP226 AT | 0.133 ± 0.835 | 54.6 | 0.160 | 0.874 |  |  |  |
| SNP226 TT | 0.412 ± 0.821 | 54.5 | 0.502 | 0.617 | 0.485 | 2,54 | 0.618 |
| **Random effects** | **Variance** | **SD** | **N** |  |  |  |  |
| Individual | <0.001 | <0.001 | 40 |  |  |  |  |
| Study site | 0.207 | 0.455 | 3 |  |  |  |  |
| Residual | 1.258 | 1.122 | 59 |  |  |  |  |
| 1. **SNP170 exon 3** | |  |  |  |  |  |  |
| **Fixed effects** | ***β* ± SE** | ***d.f.*** | ***t*** | ***P*** | ***F*** | ***d.f.*** | ***P*** |
| (Intercept) | 2.574 ± 0.300 | 2.368 | 8.570 | 0.007 |  |  |  |
| SNP170 CG | -0.382 ± 0.351 | 56.2 | -1.090 | 0.280 |  |  |  |
| SNP170 GG | -1.265± 1.152 | 55.8 | -1.098 | 0.276 | 1.149 | 2, 56 | 0.325 |
| **Random effects** | **Variance** | **SD** | **N** |  |  |  |  |
| Individual | <0.001 | <0.001 | 41 |  |  |  |  |
| Study site | 0.185 | 0.431 | 3 |  |  |  |  |
| Residual | 1.270 | 1.127 | 60 |  |  |  |  |
| 1. **SNP36 exon 6** | |  |  |  |  |  |  |
| **Fixed effects** | ***β* ± SE** | ***d.f*** | ***t*** | ***P*** | ***F*** | ***d.f.*** | ***P*** |
| Intercept | 2.724 ± 0.376 | 4.953 | 7.252 | <0.001 |  |  |  |
| SNP36 CT | -0.388 ± 0.370 | 56.0 | -1.049 | 0.298 |  |  |  |
| SNP36 TT | -0.358 ± 0.387 | 56.9 | -0.924 | 0.359 | 0.640 | 2,56 | 0.531 |
| **Random effects** | **Variance** | **SD** | **N** |  |  |  |  |
| Individual | <0.001 | <0.001 | 41 |  |  |  |  |
| Study site | 0.198 | 0.445 | 3 |  |  |  |  |
| Residual | 1.290 | 1.136 | 60 |  |  |  |  |
| 1. **SNP51 exon 9** | |  |  |  |  |  |  |
| **Fixed effects** | ***β* ± SE** | ***d.f*** | ***t*** | ***P*** | ***F*** | ***d.f.*** | ***P*** |
| Intercept | 2.507 ± 0.300 | 2.364 | 8.353 | 0.008 |  |  |  |
| SNP51 CT | -0.176 ± 0.367 | 55.7 | -0.479 | 0.634 |  |  |  |
| SNP51 TT | -0.163 ± 0.848 | 55.5 | -0.192 | 0.848 | 0.128 | 2,56 | 0.880 |
| **Random effects** | **Variance** | **SD** | **N** |  |  |  |  |
| Individual | <0.001 | 0.019 | 41 |  |  |  |  |
| Study site | 0.182 | 0.426 | 3 |  |  |  |  |
| Residual | 1.316 | 1.147 | 60 |  |  |  |  |
| 1. **SNP84 exon 9** | |  |  |  |  |  |  |
| **Fixed effects** | ***β* ± SE** | ***d.f*** | ***t*** | ***P*** | ***F*** | ***d.f.*** | ***P*** |
| Intercept | 2.766 ± 0.465 | 10.4 | 5.948 | <0.001 |  |  |  |
| SNP84 CT | -0.411 ± 0.440 | 55.2 | -0.934 | 0.354 |  |  |  |
| SNP84 TT | -0.291 ± 0.456 | 56.3 | -0.638 | 0.526 | 0.439 | 2,56 | 0.647 |
| **Random effects** | **Variance** | **SD** | **N** |  |  |  |  |
| Individual | <0.001 | <0.001 | 41 |  |  |  |  |
| Study site | 0.205 | 0.453 | 3 |  |  |  |  |
| Residual | 1.298 | 1.139 | 60 |  |  |  |  |
| 1. **SNP66 exon 13** | |  |  |  |  |  |  |
| **Fixed effects** | ***β* ± SE** | ***d.f*** | ***t*** | ***P*** | ***F*** | ***d.f.*** | ***P*** |
| Intercept | 2.701 ± 0.207 | 50 | 13.0 | <0.001 |  |  |  |
| SNP66 CT | -0.424 ± 0.325 | 50 | -1.303 | 0.199 |  |  |  |
| SNP66 TT | -0.904 ± 0.597 | 50 | -1.515 | 0.136 | 1.638 | 2,50 | 0.205 |
| **Random effects** | **Variance** | **SD** | **N** |  |  |  |  |
| Individual | <0.001 | <0.001 | 36 |  |  |  |  |
| Residual | 1.251 | 1.118 | 53 |  |  |  |  |

*^a^No males had genotype TT.*

**Reference genotypes are AA for SNP163 exon 1and SNP170 exon 3, and CC for SNP36 exon 6, SNP51 exon 9, SNP84 exon 9 and SNP66 exon 13.*

**Table S15.** GLMMs with binomial distributions for the effect of SNPs in the promoter region on the success of female great tits on obstacle removal problem-solving tests; a) SNP31, b) SNP100, c) SNP250, and d) SNP438. General generic models are shown.

| 1. **SNP31** | |  |  |  |  |
| --- | --- | --- | --- | --- | --- |
| **Fixed Effects** | ***β* ± SE** | ***Z*** | ***P*** | ***χ^2^_2_*** | ***P*** |
| Intercept | -0.878 ± 0.754 | -1.165 | 0.244 |  |  |
| SNP31 AT | -0.412 ± 0.450 | -0.915 | 0.36 |  |  |
| SNP31 TT | -0.196 ± 0.761 | -0.258 | 0.797 | 0.805 | 0.668 |
| Trial | -0.105 ± 0.420 | -0.252 | 0.801 |  |  |
| **Random effects** | **Variance** | **SD** | **N** |  |  |
| Observation | <0.001 | <0.001 | 68 |  |  |
| Individual | <0.001 | <0.001 | 47 |  |  |
| Study site | 0.401 | 0.633 | 3 |  |  |
| 1. **SNP100^a^** | |  |  |  |  |
| **Fixed Effects** | ***β* ± SE** | ***Z*** | ***P*** | ***χ^2^_2_*** | ***P*** |
| Intercept | 0.306 ± 1.041 | 0.294 | 0.769 |  |  |
| SNP100 AG | -24.1 ± 362.0 | -0.067 | 0.947 |  |  |
| Trial | -0.346 ± 0.781 | -0.444 | 0.657 |  |  |
| **Random effects** | **Variance** | **SD** | **N** |  |  |
| Observation | <0.001 | <0.001 | 65 |  |  |
| Individual | <0.001 | <0.001 | 45 |  |  |
| Study site | 0.266 | 0.516 | 3 |  |  |
| 1. **SNP250** | |  |  |  |  |
| **Fixed Effects** | ***β* ± SE** | ***Z*** | ***P*** |  |  |
| Intercept | -1.827 ± 0.857 | -2.131 | 0.0331 |  |  |
| SNP250 GG | 1.085 ± 0.733 | 1.480 | 0.1389 |  |  |
| Trial | -0.032 ± 0.429 | -0.076 | 0.9397 |  |  |
| **Random effects** | **Variance** | **SD** | **N** |  |  |
| Observation | <0.001 | <0.001 | 67 |  |  |
| Individual | <0.001 | <0.001 | 47 |  |  |
| Study site | 0.069 | 0.263 | 3 |  |  |
| 1. **SNP438** | |  |  |  |  |
| **Fixed Effects** | ***β* ± SE** | ***Z*** | ***P*** | ***χ^2^_2_*** | ***P*** |
| Intercept | -1.199 ± 0.703 | -1.706 | 0.088 |  |  |
| SNP438 TC | 0.443 ± 0.467 | 0.949 | 0.343 |  |  |
| SNP438 TT | 0.263 ± 0.819 | 0.321 | 0.748 | 0.918 | 0.631 |
| Trial | -0.141 ± 0.426 | -0.332 | 0.740 |  |  |
| **Random effects** | **Variance** | **SD** | **N** |  |  |
| Observation | <0.001 | <0.001 | 69 |  |  |
| Individual | <0.001 | <0.001 | 48 |  |  |
| Study site | 0.304 | 0.552 | 3 |  |  |

**Reference genotypes are AA for SNP31, SNP100, SNP250 and CC for SNP438.*

*^a^One individual with genotype GG dropped. Model convergence still suboptimal.*

**Table S16.** GLMMs with binomial distributions for the effect of exonic SNPs on the success of female great tits on obstacle removal problem-solving tests; a) SNP163 exon 6, b) SNP187 exon 1, c) SNP170 exon 3, d) SNP36 exon 6, e) SNP51 exon 9, f) SNP84 exon 9, and g) SNP66 exon 13. General generic models are shown.

| 1. **SNP163 exon 1** | |  |  |  |  |
| --- | --- | --- | --- | --- | --- |
| **Fixed Effects** | ***β* ± SE** | ***Z*** | ***P*** |  |  |
| Intercept | -0.931 ± 0.900 | -1.034 | 0.301 |  |  |
| SNP163 GG | -0.364 ± 0.657 | -0.554 | 0.58 |  |  |
| Trial number | 0.026 ± 0.420 | 0.063 | 0.949 |  |  |
| **Random effects** | **Variance** | **SD** | **N** |  |  |
| Observation | <0.001 | <0.001 | 72 |  |  |
| Individual | <0.001 | <0.001 | 53 |  |  |
| Study site | 0.401 | 0.633 | 3 |  |  |
| 1. **SNP187 exon 1** | |  |  |  |  |
| **Fixed Effects** | ***β* ± SE** | ***Z*** | ***P*** | ***χ^2^_2_*** | ***P*** |
| Intercept | -1.330 ± 0.680 | -1.956 | 0.050 |  |  |
| SNP187 TC | 0.279 ± 0.444 | 0.629 | 0.529 |  |  |
| SNP187 TT | 0.752 ± 0.601 | 1.251 | 0.211 | 1.468 | 0.480 |
| Trial | -0.001 ± 0.423 | -0.003 | 0.997 |  |  |
| **Random effects** | **Variance** | **SD** | **N** |  |  |
| Observation | <0.001 | <0.001 | 72 |  |  |
| Individual | <0.001 | <0.001 | 53 |  |  |
| Study site | 0.241 | 0.491 | 3 |  |  |
| 1. **SNP170 exon 3** | |  |  |  |  |
| **Fixed Effects** | ***β* ± SE** | ***Z*** | ***P*** | ***χ^2^_2_*** | ***P*** |
| Intercept | -1.275 ± 0.681 | -1.871 | 0.061 |  |  |
| SNP170 CG | 0.256 ± 0.492 | 0.520 | 0.603 |  |  |
| SNP170 GG | 0.196 ± 0.753 | 0.260 | 0.794 | 0.281 | 0.868 |
| Trial | 0.022 ± 0.421 | 0.054 | 0.956 |  |  |
| **Random effects** | **Variance** | **SD** | **N** |  |  |
| Observation | <0.001 | <0.001 | 72 |  |  |
| Individual | <0.001 | <0.001 | 53 |  |  |
| Study site | 0.301 | 0.549 | 3 |  |  |
| 1. **SNP36 exon 6** | |  |  |  |  |
| **Fixed Effects** | ***β* ± SE** | ***Z*** | ***P*** | ***χ^2^_2_*** | ***P*** |
| Intercept | -1.564 ± 0.856 | -1.827 | 0.0677 |  |  |
| SNP36 CT | 0.188 ± 0.643 | 0.292 | 0.7699 |  |  |
| SNP36 TT | 0.804 ± 0.647 | 1.242 | 0.2143 | 2.579 | 0.275 |
| Trial | -0.027 ± 0.422 | -0.066 | 0.9474 |  |  |
| **Random effects** | **Variance** | **SD** | **N** |  |  |
| Observation | <0.001 | <0.001 | 72 |  |  |
| Individual | <0.001 | <0.001 | 53 |  |  |
| Study site | 0.318 | 0.563 | 3 |  |  |
| 1. **SNP51 exon 9** | |  |  |  |  |
| **Fixed Effects** | ***β* ± SE** | ***Z*** | ***P*** | ***χ^2^_2_*** | ***P*** |
| Intercept | -1.078 ± 0.679 | -1.588 | 0.112 |  |  |
| SNP51 CT | -0.546 ± 0.628 | -0.869 | 0.385 |  |  |
| SNP51 TT | 0.841 ± 0.571 | 1.472 | 0.141 | 3.118 | 0.210 |
| Trial | -0.067 ± 0.426 | -0.159 | 0.874 |  |  |
| **Random effects** | **Variance** | **SD** | **N** |  |  |
| Observation | <0.001 | <0.001 | 72 |  |  |
| Individual | <0.001 | <0.001 | 53 |  |  |
| Study site | 0.276 | 0.525 | 3 |  |  |
| 1. **SNP84 exon 9** | |  |  |  |  |
| **Fixed Effects** | ***β* ± SE** | ***Z*** | ***P*** | ***χ^2^_2_*** | ***P*** |
| Intercept | -0.641 ± 0.753 | -0.851 | 0.394 |  |  |
| SNP84 CT | -0.832 ± 0.483 | -1.721 | 0.085 |  |  |
| SNP84 TT | -0.279 ± 0.487 | -0.573 | 0.566 | 3.057 | 0.216 |
| Trial | -0.086 ± 0.428 | -0.202 | 0.839 |  |  |
| **Random effects** | **Variance** | **SD** | **N** |  |  |
| Observation | <0.001 | <0.001 | 72 |  |  |
| Individual | <0.001 | <0.001 | 53 |  |  |
| Study site | 0.218 | 0.467 | 3 |  |  |
| 1. **SNP66 exon 13** | |  |  |  |  |
| **Fixed Effects** | ***β* ± SE** | ***Z*** | ***P*** | ***χ^2^_2_*** | ***P*** |
| Intercept | -0.897 ± 0.743 | -1.208 | 0.227 |  |  |
| SNP66 CT | -0.062 ± 0.544 | -0.114 | 0.909 |  |  |
| SNP66 TT | -23.2 ± 512 | -0.045 | 0.964 | 3.060 | 0.216 |
| Trial | -0.184 ± 0.514 | -0.358 | 0.72 |  |  |
| **Random effects** | **Variance** | **SD** | **N** |  |  |
| Observation | <0.001 | <0.001 | 64 |  |  |
| Individual | <0.001 | <0.001 | 47 |  |  |
| Study site | 0.133 | 0.365 | 3 |  |  |

**Reference genotypes are AA for SNP163 exon 1 and SNP170 exon 3, and CC for SNP187 exon 1, SNP36 exon 6, SNP51 exon 9, SNP84 exon 9 and SNP66 exon 13.*

**Table S17.** GLMMs with binomial distributions for the effect of SNPs in the promoter region on the success of male great tits on obstacle removal problem-solving tests during first trials; a) SNP31, b) SNP100, c) SNP250, and d) SNP438. General generic models are shown.

| 1. **SNP31 (n = 36)** | |  |  |  |  |
| --- | --- | --- | --- | --- | --- |
| **Fixed Effects** | ***β* ± SE** | ***Z*** | ***P*** | ***χ^2^_2_*** | ***P*** |
| Intercept | -1.609 ± 0.548 | -2.938 | 0.003 |  |  |
| SNP31 AT | 0.762 ± 0.881 | 0.865 | 0.387 |  |  |
| SNP31 TT | 1.6094 ± 1.517 | 1.061 | 0.288 | 1.522 | 0.467 |
| **Random effects** | **Variance** | **SD** | **N** |  |  |
| Study site | <0.001 | <0.001 | 3 |  |  |
| 1. **SNP100 (n =36)** | | | | | |
| **Fixed Effects** | ***β* ± SE** | ***Z*** | ***P*** |  |  |
| Intercept | -1.250 ± 0.463 | -2.706 | 0.007 |  |  |
| SNP100 AG^a^ | <0.001 ± 0.926 | 0 | 1 |  |  |
| **Random effects** | **Variance** | **SD** | **N** |  |  |
| Study site | <0.001 | <0.001 | 3 |  |  |
| 1. **SNP250 (n = 37)** | | | | | |
| **Fixed Effects** | ***β* ± SE** | ***Z*** | ***P*** |  |  |
| Intercept | -1.386 ± 0.646 | -2.148 | 0.032 |  |  |
| SNP250 GG^b^ | 0.405 ± 0.804 | 0.505 | 0.614 |  |  |
| **Random effects** | **Variance** | **SD** | **N** |  |  |
| Study site | <0.001 | <0.001 | 3 |  |  |
| 1. **SNP438 (n = 39)** | |  |  |  |  |
| **Fixed Effects** | ***β* ± SE** | ***Z*** | ***P*** |  |  |
| Intercept | -1.253 ± 0.463 | -2.706 | 0.007 |  |  |
| SNP438 TC^c^ | 0.272 ± 0.820 | 0.332 | 0.740 |  |  |
| **Random effects** | **Variance** | **SD** | **N** |  |  |
| Study site | 0.304 | 0.552 | 3 |  |  |

**Reference genotypes are AA for SNP31 and SNP100, AG for SNP250 and CC for SNP438*

*^a^No individuals with genotype GG*

*^b^Two individuals with genotype AA were dropped to facilitate model convergence.*

*^c^One individual with genotype TT was dropped to facilitate model convergence.*

**Table S18.** GLMMs with binomial distributions for the effect of exonic SNPs on the success of male great tits on obstacle removal problem-solving tests; a) SNP163 exon 6, b) SNP187 exon 1, c) SNP170 exon 3, d) SNP36 exon 6, e) SNP51 exon 9, f) SNP84 exon 9, and g) SNP66 exon 13. General generic models are shown.

| 1. **SNP163 exon 1 (*N* = 41)** | |  |  |  |  |
| --- | --- | --- | --- | --- | --- |
| **Fixed Effects** | ***β* ± SE** | ***Z*** | ***P*** |  |  |
| Intercept | -1.203 ± 0.658 | -1.829 | 0.067 |  |  |
| SNP163 GG^a^ | -0.048 ± 0805 | -0.061 | 0.951 |  |  |
| **Random effects** | **Variance** | **SD** | **N** |  |  |
| Study site | <0.001 | <0.001 | 3 |  |  |
| 1. **SNP187 exon 1 (*N =* 41)** | |  |  |  |  |
| **Fixed Effects** | ***β* ± SE** | ***Z*** | ***P*** |  |  |
| Intercept | -1.049 ± 0.439 | -2.391 | 0.016 |  |  |
| SNP187 TC^b^ | -0.654 ± 0.885 | -0.740 | 0.459 |  |  |
| **Random effects** | **Variance** | **SD** | **N** |  |  |
| Study site | <0.001 | <0.001 | 3 |  |  |
| 1. **SNP170 exon 3 (*N* = 41)** | |  |  |  |  |
| **Fixed Effects** | ***β* ± SE** | ***Z*** | ***P*** |  |  |
| Intercept | -1.427 ± 0.454 | -3.139 | 0.002 |  |  |
| SNP170 CG^c^ | 0.734 ± 0.841 | 0.873 | 0.382 |  |  |
| **Random effects** | **Variance** | **SD** | **N** |  |  |
| Study site | <0.001 | <0.001 | 3 |  |  |
| 1. **SNP36 exon 6 (*N* = 41)** | |  |  |  |  |
| **Fixed Effects** | ***β* ± SE** | ***Z*** | ***P*** | ***χ^2^_2_*** | ***P*** |
| Intercept | -1.705 ± 0.769 | -2.218 | 0.026 |  |  |
| SNP36 CT | 0.318 ± 1.004 | 0.317 | 0.751 |  |  |
| SNP36 TT | 0.894 ± 0.976 | 0.916 | 0.359 | 0.933 | 0.627 |
| **Random effects** | **Variance** | **SD** | **N** |  |  |
| Study site | 0.318 | 0.563 | 3 |  |  |
| 1. **SNP51 exon 9 (*N*= 40)** | |  |  |  |  |
| **Fixed Effects** | ***β* ± SE** | ***Z*** | ***P*** |  |  |
| Intercept | -1.909 ± 0.535 | -3.564 | <0.001 |  |  |
| SNP51 CT^d^ | 1.686 ± 0.859 | 1.964 | 0.049 |  |  |
| **Random effects** | **Variance** | **SD** | **N** |  |  |
| Study site | <0.001 | <0.001 | 3 |  |  |
| 1. **SNP84 exon 9 (*N* = 41)** | |  |  |  |  |
| **Fixed Effects** | ***β* ± SE** | ***Z*** | ***P*** | ***χ^2^_2_*** | ***P*** |
| Intercept | 1.531 ± 1.307 | 1.172 | 0.2413 |  |  |
| SNP84 CT | -3.771 ± 1.585 | -2.38 | 0.045 |  |  |
| SNP84 TT | -3.075 ± 1.607 | -1.914 | 0.134 | 9.77 | 0.007 |
| **Random effects** | **Variance** | **SD** | **N** |  |  |
| Study site | 0.218 | 0.467 | 3 |  |  |
| 1. **SNP66 exon 13 (*N* = 33)** | |  |  |  |  |
| **Fixed Effects** | ***β* ± SE** | ***Z*** | ***P*** |  |  |
| Intercept | -0.619 ± 0.469 | -1.32 | 0.187 |  |  |
| SNP66 CT^e^ | -1.086 ± 0.900 | -1.206 | 0.228 |  |  |
| **Random effects** | **Variance** | **SD** | **N** |  |  |
| Study site | <0.001 | <0.001 | 3 |  |  |

**Reference genotypes are AG for SNP163 exon, AA for SNP170 exon 3, and CC for SNP187 exon 1, SNP36 exon 6, SNP51 exon 9, SNP84 exon 9 and SNP66 exon 13*

*^a^No individuals with genotype AA*

*^b^No individuals with genotype TT*

*^c^One individual with genotype GG was dropped to facilitate model convergence*

*^d^One individual with genotype TT was dropped to facilitate model convergence*

*^e^Three individuals with genotype TT were dropped to facilitate model convergence*

**Table S19**. General generic LMMs with Satterthwaite approximations for degrees of freedom for the relationship between SNPs in the promoter region and distance from the nearest road or path (square-root transformed). a) SNP31, b) SNP100, c) SNP250, d) SNP438.

| 1. **SNP 31** | | | |  |  |  |  |
| --- | --- | --- | --- | --- | --- | --- | --- |
| **Fixed effects** | ***β* ± SE** | ***t*** | ***d.f.*** | ***P*** | ***F*** | ***d.f.*** | ***P*** |
| Intercept | 5.51 ± 1.73 | 3.18 | 3.06 | 0.048 |  |  |  |
| SNP31 AT | -0.110 ± 0.532 | -0.208 | 147 | 0.835 |  |  |  |
| SNP31 TT | -0.521 ± 1.13 | -0.463 | 147 | 0.644 | 0.116 | 2, 147 | 0.890 |
| **Random effects** | **Variance** | **SD** | **N** |  |  |  |  |
| Study site | 11.5 | 3.40 | 4 |  |  |  |  |
| Residual | 8.00 | 2.83 | 153 |  |  |  |  |
| 1. **SNP 100** | | | | | | | |
| **Fixed effects** | ***β* ± SE** | ***t*** | ***d.f.*** | ***P*** | ***F*** | ***d.f.*** | ***P*** |
| Intercept | 5.67 ± 1.75 | 3.24 | 3.02 | 0.047 |  |  |  |
| SNP100 AG/GG | -0.810 ± 0.552 | -1.47 | 148 | 0.144 | 2.15 | 1, 149 | 0.145 |
| **Random effects** | **Variance** | **SD** | **N** |  |  |  |  |
| Study site | 11.9 | 3.45 | 4 |  |  |  |  |
| Residual | 7.76 | 2.78 | 153 |  |  |  |  |
| 1. **SNP 250** | | | | | | | |
| **Fixed effects** | ***β* ± SE** | ***t*** | ***d.f.*** | ***P*** | ***F*** | ***d.f.*** | ***P*** |
| Intercept | 5.34 ± 1.91 | 2.79 | 4.22 | 0.046 |  |  |  |
| SNP250 AG | -0.207 ± 0.892 | -0.233 | 154 | 0.816 |  |  |  |
| SNP250 GG | 0.320 ± 0.830 | 0.386 | 154 | 0.700 | 0.540 | 2, 154 | 0.584 |
| **Random effects** | **Variance** | **SD** | **N** |  |  |  |  |
| Study site | 12.0 | 3.47 | 4 |  |  |  |  |
| Residual | 7.78 | 2.78 | 169 |  |  |  |  |
| 1. **SNP438** | | | | | | | |
| **Fixed effects** | ***β* ± SE** | ***t*** | ***d.f.*** | ***P*** | ***F*** | ***d.f.*** | ***P*** |
| Intercept | 5.63 ± 1.70 | 3.30 | 3.09 | 0.043 |  |  |  |
| SNP438 TC | -0.288 ± 0.484 | -0.596 | 154 | 0.552 |  |  |  |
| SNP438 TT | -0.105 ± 0.895 | -0.117 | 154 | 0.907 | 0.177 | 2, 154 | 0.838 |
| **Random effects** | **Variance** | **SD** | **N** |  |  |  |  |
| Study site | 11.2 | 3.34 | 4 |  |  |  |  |
| Residual | 7.82 | 2.79 | 160 |  |  |  |  |

**Reference genotypes are AA for SNP31, SNP100, SNP250 and CC for SNP438.*

**Table S20**. General generic LMMs for the relationship between exonic SNPs and distance from the nearest road or path (square-root transformed). (a) nonsynonymous SNP226 exon 1, b) SNP163 exon 1, c) SNP187 exon 1, d) SNP170 exon 3, e) SNP36 exon 6, f) SNP51 exon 9, g) SNP84 exon 9, h) SNP66 exon 13.

| 1. **SNP226 exon 1** | | | |  |  |  |  |
| --- | --- | --- | --- | --- | --- | --- | --- |
| **Fixed effects** | ***β* ± SE** | ***t*** | ***d.f.*** | ***P*** | ***F*** | ***d.f.*** | ***P*** |
| Intercept | 6.13 ± 1.80 | 3.39 | 3.84 | 0.029 |  |  |  |
| SNP226 AT | -0.592 ± 0.758 | -0.780 | 163 | 0.436 |  |  |  |
| SNP226 TT | -0.688 ± 0.750 | -0.917 | 164 | 0.360 | 0.422 | 2,163 | 0.656 |
| **Random effects** | **Variance** | **SD** | **N** |  |  |  |  |
| Study site | 11.2 | 3.35 | 4 |  |  |  |  |
| Residual | 7.78 | 2.79 | 169 |  |  |  |  |
| 1. **SNP163 exon 1** | | | | | | | |
| **Fixed effects** | ***β* ± SE** | ***t*** | ***d.f.*** | ***P*** | ***F*** | ***d.f.*** | ***P*** |
| Intercept | 5.61 ± 2.02 | 2.773 | 6.64 | 0.029 |  |  |  |
| SNP163 AG | -0.162 ± 1.24 | -0.130 | 163 | 0.896 |  |  |  |
| SNP163 GG | 0.008 ± 1.20 | 0.007 | 163 | 0.994 | 0.057 | 2, 163 | 0.843 |
| **Random effects** | **Variance** | **SD** | **N** |  |  |  |  |
| Study site | 10.7 | 3.28 | 4 |  |  |  |  |
| Residual | 7.82 | 2.80 | 169 |  |  |  |  |
| 1. **SNP187 exon 1** | | | | | | | |
| **Fixed effects** | ***β* ± SE** | ***t*** | ***d.f.*** | ***P*** | ***F*** | ***d.f.*** | ***P*** |
| Intercept | 5.50 ± 0.166 | 3.30 | 3.07 | 0.044 |  |  |  |
| SNP187 TC | 0.241 ± 0.475 | 0.509 | 163 | 0.611 |  |  |  |
| SNP187 TT | -0.145 ± 1.19 | -0.123 | 163 | 0.903 | 0.147 | 2, 163 | 0.863 |
| **Random effects** | **Variance** | **SD** | **N** |  |  |  |  |
| Study site | 10.8 | 3.28 | 4 |  |  |  |  |
| Residual | 7.82 | 2.76 | 169 |  |  |  |  |
| 1. **SNP170 exon 3** | | | | | | | |
| **Fixed effects** | ***β* ± SE** | ***t*** | ***d.f.*** | ***P*** | ***F*** | ***d.f.*** | ***P*** |
| Intercept | 5.58 ± 1.64 | 1.64 | 3.04 | 0.041 |  |  |  |
| SNP170 CG | -0.072 ± 0.601 | 0.601 | 168 | 0.904 |  |  |  |
| SNP170 GG | -0.537 ± 1.19 | 1.18 | 168. | 0.650 | 0.106 | 2,168 | 0.899 |
| **Random effects** | **Variance** | **SD** | **N** |  |  |  |  |
| Study site | 10.5 | 3.24 | 4 |  |  |  |  |
| Residual | 7.83 | 2.80 | 174 |  |  |  |  |
| 1. **SNP36 exon 6** | |  |  |  |  |  |  |
| **Fixed effects** | ***β* ± SE** | ***t*** | ***d.f.*** | ***P*** | ***F*** | ***d.f.*** | ***P*** |
| Intercept | 5.11 ± 1.74 | 2.94 | 3.32 | 0.053 |  |  |  |
| SNP36 CT | 0.645 ± 0.542 | 1.19 | 167 | 0.235 |  |  |  |
| SNP36 TT | 0.302 ± 0.564 | 0.536 | 167 | 0.592 | 0.732 | 2,167 | 0.483 |
| **Random effects** | **Variance** | **SD** | **N** |  |  |  |  |
| Study site | 11.2 | 3.34 | 4 |  |  |  |  |
| Residual | 7.75 | 2.78 | 173 |  |  |  |  |
| 1. **SNP51 exon 9** | |  |  |  |  |  |  |
| **Fixed effects** | ***β* ± SE** | ***t*** | ***d.f.*** | ***P*** | ***F*** | ***d.f.*** | ***P*** |
| Intercept | 5.36 ± 1.72 | 3.12 | 3.04 | 0.051 |  |  |  |
| SNP51 CT | 0.326 ± 0.529 | 0.617 | 166 | 0.537 |  |  |  |
| SNP51 TT | 1.41 ± 1.05 | 1.34 | 166 | 0.180 | 1.04 | 2,166 | 0.355 |
| **Random effects** | **Variance** | **SD** | **N** |  |  |  |  |
| Study site | 11.5 | 3.39 | 4 |  |  |  |  |
| Residual | 7.70 | 2.78 | 172 |  |  |  |  |
| 1. **SNP84 exon 9** | |  |  |  |  |  |  |
| **Fixed effects** | ***β* ± SE** | ***t*** | ***d.f.*** | ***P*** | ***F*** | ***d.f.*** | ***P*** |
| Intercept | 5.35 ± 1.74 | 3.08 | 3.33 | 0.046 |  |  |  |
| SNP84 CT | 0.125 ± 0.567 | 0.222 | 166 | 0.825 |  |  |  |
| SNP84 TT | 0.317 ± 0.605 | 0.525 | 166 | 0.600 | 0.149 | 2,166 | 0.862 |
| **Random effects** | **Variance** | **SD** | **N** |  |  |  |  |
| Study site | 11.2 | 3.34 | 4 |  |  |  |  |
| Residual | 7.78 | 2.79 | 172 |  |  |  |  |
| 1. **SNP66 exon 13** | |  |  |  |  |  |  |
| **Fixed effects** | ***β* ± SE** | ***t*** | ***d.f.*** | ***P*** | ***F*** | ***d.f.*** | ***P*** |
| Intercept | 5.42 ± 1.64 | 3.31 | 3.08 | 0.043 |  |  |  |
| SNP66 CT | 0.348 ± 0.473 | 0.737 | 154 | 0.462 |  |  |  |
| SNP66 TT | -0.717 ± 0.853 | -0.841 | 154 | 0.401 | 0.794 | 2, 154 | 0.454 |
| **Random effects** | **Variance** | **SD** | **N** |  |  |  |  |
| Study site | 10.3 | 3.21 | 4 |  |  |  |  |
| Residual | 7.50 | 2.74 | 160 |  |  |  |  |

**Reference genotypes are AA for SNP163 exon 1 and SNP170 exon 3, and CC for SNP187 exon 1, SNP36 exon 6, SNP51 exon 9, SNP84 exon 9 and SNP66 exon 13.*

**Table S21**. General generic LMMs for the relationship between SNPs in the promoter region and log-transformed laying date in females. a) SNP31, b) SNP100, c) SNP250, d) SNP438.

| 1. **SNP31** | | | |  |  |  |  |
| --- | --- | --- | --- | --- | --- | --- | --- |
| **Fixed effects** | ***β* ± SE** | ***t*** | ***d.f.*** | ***P*** | ***F*** | ***d.f.*** | ***P*** |
| Intercept | 4.658 ± 0.039 | 119.0 | 1.09 | 0.003 |  |  |  |
| SNP31 AT | 0.012 ± 0.009 | 1.298 | 70.2 | 0.198 |  |  |  |
| SNP31 TT | 0.021 ± 0.021 | 0.995 | 65.6 | 0.323 | 1.145 | 2,67 | 0.324 |
| **Random effects** | **Variance** | **SD** | **N** |  |  |  |  |
| Individual | <0.001 | 0.009 | 82 |  |  |  |  |
| Study site | <0.001 | 0.014 | 4 |  |  |  |  |
| Year | 0.003 | 0.053 | 2 |  |  |  |  |
| Residual | 0.001 | 0.039 | 107 |  |  |  |  |
| 1. **SNP100** | | | | | | | |
| **Fixed effects** | ***β* ± SE** | ***t*** | ***d.f.*** | ***P*** | ***F*** | ***d.f.*** | ***P*** |
| Intercept | 4.663 ± 0.038 | 119.8 | 1.075 | 0.004 |  |  |  |
| SNP100 AG | -0.003 ± 0.010 | -0.303 | 69.2 | 0.763 |  |  |  |
| SNP100 GG | 0.022 ± 0.031 | 0.714 | 31.5 | 0.714 | 0.334 | 2,43 | 0.717 |
| **Random effects** | **Variance** | **SD** | **N** |  |  |  |  |
| Individual | <0.001 | 0.011 | 82 |  |  |  |  |
| Study site | <0.001 | 0.011 | 4 |  |  |  |  |
| Year | 0.002 | 0.053 | 2 |  |  |  |  |
| Residual | 0.001 | 0.039 | 107 |  |  |  |  |
| 1. **SNP250** | | | | | | | |
| **Fixed effects** | ***β* ± SE** | ***t*** | ***d.f.*** | ***P*** | ***F*** | ***d.f.*** | ***P*** |
| Intercept | 4.653 ± 0.040 | 114.3 | 1.329 | 0.001 |  |  |  |
| SNP250 AG | 0.015 ± 0.015 | 1.003 | 69.6 | 0.319 |  |  |  |
| SNP250 GG | 0.009 ± 0.015 | 0.631 | 76.2 | 0.529 | 0.558 | 2,72 | 0.574 |
| **Random effects** | **Variance** | **SD** | **N** |  |  |  |  |
| Individual | <0.001 | 0.008 | 86 |  |  |  |  |
| Study site | 1.099 | 0.010 | 4 |  |  |  |  |
| Year | 0.002 | 0.053 | 2 |  |  |  |  |
| Residual | 0.001 | 0.039 | 112 |  |  |  |  |
| 1. **SNP438** | | | | | | | |
| **Fixed effects** | ***β*  SE** | ***t*** | ***d.f.*** | ***P*** | ***F*** | ***d.f.*** | ***P*** |
| Intercept | 4.672 ± 0.039 | 117.6 | 1.052 | 0.004 |  |  |  |
| SNP 438 TC | -0.016 ± 0.008 | -1.928 | 74.6 | 0.057 |  |  |  |
| SNP 438 TT | -0.014 ± 0.014 | -1.000 | 73.8 | 0.320 | 2.029 | 2,73 | 0.138 |
| **Random effects** | **Variance** | **SD** | **N** |  |  |  |  |
| Individual | < 0.001 | 0.008 | 86 |  |  |  |  |
| Study site | < 0.001 | 0.009 | 4 |  |  |  |  |
| Year | 0.003 | 0.055 | 2 |  |  |  |  |
| Residual | 0.001 | 0.039 | 112 |  |  |  |  |

**Reference genotypes are AA for SNP31, SNP100, SNP250 and CC for SNP438.*

**Table S22**. General generic LMMs for the relationship between exonic SNPs and log-transformed laying date in females; a) SNP226 exon 1, b) SNP163 exon 1, c) SNP187 exon 1, d) SNP36 exon 6, e) SNP51 exon 9, f) SNP66 exon 13.

| 1. **SNP226 exon 1** | | | |  |  |  |  |
| --- | --- | --- | --- | --- | --- | --- | --- |
| **Fixed effects** | ***β* ± SE** | ***t*** | ***d.f.*** | ***P*** | ***F*** | ***d.f.*** | ***P*** |
| Intercept | 4.654 ± 0.040 | 115.9 | 1.20 | 0.002 |  |  |  |
| SNP226 AT | 0.011 ± 0.013 | 0.863 | 88.2 | 0.390 |  |  |  |
| SNP226 TT | 0.011 ± 0.013 | 0.868 | 82.6 | 0.387 | 0.426 | 2,77 | 0.654 |
| **Random effects** | **Variance** | **SD** | **N** |  |  |  |  |
| Individual | < 0.001 | 0.012 | 90 |  |  |  |  |
| Study site | < 0.001 | 0.011 | 4 |  |  |  |  |
| Year | 0.002 | 0.053 | 2 |  |  |  |  |
| Residual | 0.001 | 0.040 | 116 |  |  |  |  |
| 1. **SNP163 exon 1** | | | | | | | |
| **Fixed effects** | ***β* ± SE** | ***t*** | ***d.f.*** | ***P*** | ***F*** | ***d.f.*** | ***P*** |
| Intercept | 4.680 ± 0.049 | 94.1 | 2.75 | <0.001 |  |  |  |
| SNP163 AG | -0.014 ± 0.032 | -0.443 | 108.7 | 0.659 |  |  |  |
| SNP163 GG | -1.067 ± 2.393 | -0.571 | 110.3 | 0.569 | 0.203 | 2,93 | 0.816 |
| **Random effects** | **Variance** | **SD** | **N** |  |  |  |  |
| Individual | < 0.001 | 0.014 | 90 |  |  |  |  |
| Study site | < 0.001 | 0.013 | 4 |  |  |  |  |
| Year | 0.002 | 0.054 | 2 |  |  |  |  |
| Residual | 0.001 | 0.040 | 116 |  |  |  |  |
| 1. **SNP187 exon 1** | | | | | | | |
| **Fixed effects** | ***β* ± SE** | ***t*** | ***d.f.*** | ***P*** | ***F*** | ***d.f.*** | ***P*** |
| Intercept | 4.664 ± 0.038 | 120.0 | 1.076 | 0.003 |  |  |  |
| SNP187 CT | -0.001 ± 0.009 | -0.118 | 71.7 | 0.906 |  |  |  |
| SNP187 TT | -0.001 ± 0.018 | -0.077 | 83.7 | 0.938 | 0.008 | 2,77 | 0.991 |
| **Random effects** | **Variance** | **SD** | **N** |  |  |  |  |
| Individual | < 0.001 | 0.013 | 90 |  |  |  |  |
| Study site | < 0.001 | 0.012 | 4 |  |  |  |  |
| Year | 0.002 | 0.053 | 2 |  |  |  |  |
| Residual | 0.001 | 0.040 | 116 |  |  |  |  |
| 1. **SNP36 exon 6** | |  |  |  |  |  |  |
| **Fixed effects** | ***β* ± SE** | ***t*** | ***d.f.*** | ***P*** | ***F*** | ***d.f.*** | ***P*** |
| Intercept | 4.671 ± 0.039 | 119.4 | 1.137 | 0.002 |  |  |  |
| SNP36 CT | -0.003 ± 0.010 | -0.344 | 67.5 | 0.731 |  |  |  |
| SNP36 TT | -0.017 ± 0.011 | -1.529 | 66.0 | 0.131 | 1.488 | 2,69 | 0.232 |
| **Random effects** | **Variance** | **SD** | **N** |  |  |  |  |
| Individual | < 0.001 | 0.011 | 91 |  |  |  |  |
| Study site | < 0.001 | 0.010 | 4 |  |  |  |  |
| Year | 0.002 | 0.053 | 2 |  |  |  |  |
| Residual | 0.001 | 0.040 | 117 |  |  |  |  |
| 1. **SNP51 exon 9** | |  |  |  |  |  |  |
| **Fixed effects** | ***β* ± SE** | ***t*** | ***d.f.*** | ***P*** | ***F*** | ***d.f.*** | ***P*** |
| Intercept | 4.665 ± 0.038 | 122.1 | 1.05 | 0.003 |  |  |  |
| SNP51 CT | 0.001 ± 0.010 | 0.147 | 73.8 | 0.749 |  |  |  |
| SNP51 TT | -0.033 ± 0.018 | -1.810 | 87.5 | 0.074 | 1.682 | 2, 80 | 0.192 |
| **Random effects** | **Variance** | **SD** | **N** |  |  |  |  |
| Individual | < 0.001 | 0.12 | 91 |  |  |  |  |
| Study site | < 0.001 | 0.011 | 4 |  |  |  |  |
| Year | 0.002 | 0.052 | 2 |  |  |  |  |
| Residual | 0.001 | 0.040 | 117 |  |  |  |  |
| 1. **SNP66 exon13** | |  |  |  |  |  |  |
| **Fixed effects** | ***β* ± SE** | ***t*** | ***d.f.*** | ***P*** | ***F*** | ***d.f.*** | ***P*** |
| Intercept | 4.663 ± 0.038 | 120.8 | 1.07 | 0.003 |  |  |  |
| SNP66 CT | 0.001 ± 0.008 | 0.231 | 77.1 | 0.818 |  |  |  |
| SNP66 TT | -0.014 ± 0.015 | -0.898 | 73.5 | 0.372 | 0.502 | 2, 75 | 0.607 |
| **Random effects** | **Variance** | **SD** | **N** |  |  |  |  |
| Individual | < 0.001 | 0.012 | 86 |  |  |  |  |
| Study site | < 0.001 | 0.012 | 4 |  |  |  |  |
| Year | 0.002 | 0.053 | 2 |  |  |  |  |
| Residual | 0.001 | 0.038 | 112 |  |  |  |  |

**Reference genotypes are AA for SNP163 exon 1 and SNP170 exon 3, and CC for SNP187 exon 1, SNP36 exon 6, SNP51 exon 9, SNP84 exon 9 and SNP66 exon 13.*

**Table S23**. General generic GLMMs with a Poisson distribution for relationships between SNPs in the promoter region and number of fledglings in females; a) SNP31, b) SNP100, c) SNP250, d) SNP438.

| 1. **SNP31** | | | | | |
| --- | --- | --- | --- | --- | --- |
| **Fixed effects** | ***β* ± SE** | ***Z*** | ***P*** | ***χ^2^_2_*** | ***P*** |
| Intercept | 2.007 ± 0.046 | 43.6 | <0.001 |  |  |
| SNP31 AT | -0.126 ± 0.080 | -1.566 | 0.117 |  |  |
| SNP31 TT | 0.266 ± 0.169 | 1.571 | 0.116 | 5.200 | 0.074 |
| **Random effects** | **Variance** | **SD** | **N** |  |  |
| Individual | 0.002 | 0.052 | 82 |  |  |
| Study site | <0.001 | <0.001 | 4 |  |  |
| Year | <0.001 | <0.001 | 2 |  |  |
| 1. **SNP100** | | | | | |
| **Fixed effects** | ***β* ± SE** | ***Z*** | ***P*** | ***χ^2^_2_*** | ***P*** |
| Intercept | 1.972 ± 0.047 | 41.7 | <0.001 |  |  |
| SNP100 AG | 0.017 ± 0.098 | 0.179 | 0.858 |  |  |
| SNP100 GG | 0.032 ± 0.300 | 0.108 | 0.914 | 0.039 | 0.980 |
| **Random effects** | **Variance** | **SD** | **N** |  |  |
| Individual | 0.018 | 0.135 | 82 |  |  |
| Study site | <0.001 | 0.013 | 4 |  |  |
| Year | <0.001 | <0.001 | 2 |  |  |
| 1. **SNP250** | | | | | |
| **Fixed effects** | ***β* ± SE** | ***Z*** | ***P*** | ***χ^2^_2_*** | ***P*** |
| Intercept | 2.090 ± 0.125 | 16.6 | <0.001 |  |  |
| SNP250 AG | -0.036 ± 0.141 | -0.258 | 0.796 |  |  |
| SNP250 GG | -0.149 ± 0.134 | -1.108 | 0.268 | 2.429 | 0.296 |
| **Random effects** | **Variance** | **SD** | **N** |  |  |
| Individual | 0.012 | 0.112 | 86 |  |  |
| Study site | <0.001 | <0.001 | 4 |  |  |
| Year | <0.001 | <0.001 | 2 |  |  |
| 1. **SNP438** | | | | | |
| **Fixed effects** | ***β* ± SE** | ***Z*** | ***P*** | ***χ^2^_2_*** | ***P*** |
| Intercept | 2.001 ± 0.057 | 35.0 | <0.001 |  |  |
| SNP438 TC | -0.068 ± 0.081 | -0.837 | 0.403 |  |  |
| SNP438 TT | 0.101 ± 0.134 | 0.751 | 0.452 | 1.606 | 0.447 |
| **Random effects** | **Variance** | **SD** | **N** |  |  |
| Individual | 0.010 | 0.100 | 86 |  |  |
| Study site | 0.002 | 0.041 | 4 |  |  |
| Year | <0.001 | <0.001 | 2 |  |  |

**Reference genotypes are AA for SNP31, SNP100, SNP250 and CC for SNP438.*

**Table S24**. General generic GLMMs with a Poisson distribution for relationships between SNPs in the promoter region and number of fledglings in males. a) SNP31, b) SNP100, c) SNP250, d) SNP438.

| 1. **SNP31** | | | | | |
| --- | --- | --- | --- | --- | --- |
| **Fixed effects** | ***β* ± SE** | ***Z*** | ***P*** | ***χ^2^_2_*** | ***P*** |
| Intercept | 2.061 ± 0.067 | 30.5 | <0.001 |  |  |
| SNP31 AT | -0.064 ± 0.129 | -0.499 | 0.618 |  |  |
| SNP31 TT | 0.058 ± 0.211 | 0.279 | 0.780 | 0.384 | 0.825 |
| **Random effects** | **Variance** | **SD** | **N** |  |  |
| Individual | < 0.001 | < 0.001 | 37 |  |  |
| Study site | < 0.001 | < 0.001 | 3 |  |  |
| Year | < 0.001 | < 0.001 | 2 |  |  |
| 1. **SNP100** | | | | | |
| **Fixed effects** | ***β* ± SE** | ***Z*** | ***P*** |  |  |
| Intercept | 2.008 ± 0.065 | 30.5 | <0.001 |  |  |
| SNP100 AG^a^ | 0.147 ± 0.121 | 1.211 | 0.226 |  |  |
| **Random effects** | **Variance** | **SD** | **N** |  |  |
| Individual | < 0.001 | < 0.001 | 37 |  |  |
| Study site | < 0.001 | < 0.001 | 4 |  |  |
| Year | < 0.001 | < 0.001 | 2 |  |  |
| 1. **SNP250** | | | | | |
| **Fixed effects** | ***β* ± SE** | ***Z*** | ***P*** | ***χ^2^_2_*** | ***P*** |
| Intercept | 1.945 ± 0.267 | 7.281 | < 0.001 |  |  |
| SNP250 AG | 0.187 ± 0.279 | 0.672 | 0.502 |  |  |
| SNP250 GG | 0.055 ± 0.277 | 0.200 | 0.841 | 1.624 | 0.443 |
| **Random effects** | **Variance** | **SD** | **N** |  |  |
| Individual | < 0.001 | < 0.001 | 40 |  |  |
| Study site | < 0.001 | < 0.001 | 3 |  |  |
| Year | < 0.001 | < 0.001 | 2 |  |  |
| 1. **SNP438** | | | | | |
| **Fixed effects** | ***β* ± SE** | ***Z*** | ***P*** | ***χ^2^_2_*** | ***P*** |
| Intercept | 2.071 ± 0.062 | 33.0 | < 0.001 |  |  |
| SNP438 TC | -0.075 ± 0.127 | -0.588 | 0.556 |  |  |
| SNP438 TT | -0.007 ± 0.257 | -0.030 | 0.976 | 0.361 | 0.834 |
| **Random effects** | **Variance** | **SD** | **N** |  |  |
| Individual | < 0.001 | < 0.001 | 40 |  |  |
| Study site | < 0.001 | < 0.001 | 3 |  |  |
| Year | < 0.001 | < 0.001 | 2 |  |  |

**Reference genotypes are AA for SNP31, SNP100, SNP250 and CC for SNP438.*

*^a^No males had genotype GG.*

**Table S25**. General generic GLMMs with a Poisson distribution for relationships between exonic SNPs and number of fledglings in females; (a) SNP226 exon 1, b) SNP163 exon 1, c) SNP187 exon 1, d) SNP170 exon 3, e) SNP36 exon 6, f) SNP51 exon 9, g) SNP84 exon 9, h) SNP66 exon 13.

| 1. **SNP226 exon 1** | | | | | |
| --- | --- | --- | --- | --- | --- |
| **Fixed effects** | ***β* ± SE** | ***Z*** | ***P*** | ***χ^2^_2_*** | ***P*** |
| Intercept | 2.005 ± 0.105 | 19.0 | < 0.001 |  |  |
| SNP226 AT | 0.003 ± 0.117 | 0.033 | 0.974 |  |  |
| SNP226 TT | -0.054 ± 0.118 | -0.464 | 0.643 | 0.529 | 0.767 |
| **Random effects** | **Variance** | **SD** | **N** |  |  |
| Individual | 0.010 | 0.103 | 90 |  |  |
| Study site | 0.003 | 0.062 | 4 |  |  |
| Year | < 0.001 | < 0.001 | 2 |  |  |
| 1. **SNP163 exon 1** | | | | | |
| **Fixed effects** | ***β* ± SE** | ***Z*** | ***P*** | ***χ^2^_2_*** | ***P*** |
| Intercept | 2.336 ± 0.233 | 9.99 | < 0.001 |  |  |
| SNP163 AG | -0.372 ± 0.246 | -1.512 | 0.131 |  |  |
| SNP163 GG | -0.357 ± 0.235 | -1.518 | 0.129 | 2.158 | 0.339 |
| **Random effects** | **Variance** | **SD** | **N** |  |  |
| Individual | 0.009 | 0.099 | 90 |  |  |
| Study site | 0.002 | 0.046 | 4 |  |  |
| Year | < 0.001 | < 0.001 | 2 |  |  |
| 1. **SNP187 exon 1** | | | | | |
| **Fixed effects** | ***β* ± SE** | ***Z*** | ***P*** | ***χ^2^_2_*** | ***P*** |
| Intercept | 1.942 ± 0.060 | 32.2 | < 0.001 |  |  |
| SNP187 TC | 0.087 ± 0.080 | 1.083 | 0.279 |  |  |
| SNP187 TT | 0.192 ± 0.158 | 1.212 | 0.225 | 2.124 | 0.345 |
| **Random effects** | **Variance** | **SD** | **N** |  |  |
| Individual | 0.008 | 0.093 | 90 |  |  |
| Study site | 0.004 | 0.066 | 4 |  |  |
| Year | < 0.001 | < 0.001 | 2 |  |  |
| 1. **SNP170 exon 3** | | | | | |
| **Fixed effects** | ***β* ± SE** | ***Z*** | ***P*** | ***χ^2^_2_*** | ***P*** |
| Intercept | 1.996 ± 0.048 | 41.0 | < 0.001 |  |  |
| SNP170 CG | -0.054 ± 0.106 | -0.435 | 0.663 |  |  |
| SNP170 GG | -0.123 ± 0.191 | -0.646 | 0.518 | 0.579 | 0.748 |
| **Random effects** | **Variance** | **SD** | **N** |  |  |
| Individual | 0.011 | 0.105 | 91 |  |  |
| Study site | 0.001 | 0.044 | 4 |  |  |
| Year | < 0.001 | < 0.001 | 2 |  |  |
| 1. **SNP36 exon 6** | |  |  |  |  |
| **Fixed effects** | ***β* ± SE** | ***Z*** | ***P*** | ***χ^2^_2_*** | ***P*** |
| Intercept | 2.136 ± 0.072 | 29.3 | < 0.001 |  |  |
| SNP36 CT | -0.216 ± 0.089 | -2.411 | 0.042^a^ |  |  |
| SNP36 TT | -0.136 ± 0.091 | -1.493 | 0.294^a^ | 5.260 | 0.072 |
| **Random effects** | **Variance** | **SD** | **N** |  |  |
| Individual | < 0.001 | < 0.001 | 91 |  |  |
| Study site | < 0.001 | 0.015 | 4 |  |  |
| Year | < 0.001 | < 0.001 | 2 |  |  |
| 1. **SNP51 exon 9** | |  |  |  |  |
| **Fixed effects** | ***β* ± SE** | ***Z*** | ***P*** | ***χ^2^_2_*** | ***P*** |
| Intercept | 1.971 ± 0.053 | 36.9 | < 0.001 |  |  |
| SNP51 CT | 0.026 ± 0.092 | 0.288 | 0.774 |  |  |
| SNP51 TT | 0.113 ± 0.166 | 0.680 | 0.497 | 0.514 | 0.773 |
| **Random effects** | **Variance** | **SD** | **N** |  |  |
| Individual | 0.010 | 0.103 | 91 |  |  |
| Study site | 0.003 | 0.057 | 4 |  |  |
| Year | < 0.001 | < 0.001 | 2 |  |  |
| 1. **SNP84 exon 9** | |  |  |  |  |
| **Fixed effects** | ***β* ± SE** | ***Z*** | ***P*** | ***χ^2^_2_*** | ***P*** |
| Intercept | 2.075 ± 0.084 | 24.5 | < 0.001 |  |  |
| SNP84 CT | -0.129 ± 0.084 | -1.367 | 0.171 |  |  |
| SNP84 TT | -0.109 ± 0.106 | -1.035 | 0.301 | 1.844 | 0.397 |
| **Random effects** | **Variance** | **SD** | **N** |  |  |
| Individual | 0.008 | 0.089 | 91 |  |  |
| Study site | 0.005 | 0.071 | 4 |  |  |
| Year | < 0.001 | < 0.001 | 2 |  |  |
| 1. **SNP66 exon 13** | |  |  |  |  |
| **Fixed effects** | ***β* ± SE** | ***Z*** | ***P*** | ***χ^2^_2_*** | ***P*** |
| Intercept | 1.964 ± 0.057 | 34.0 | <0.001 |  |  |
| SNP13.66 CT | 0.029 ± 0.086 | 0.343 | 0.732 |  |  |
| SNP13.66 TT | 0.039 ± 0.152 | 0.262 | 0.794 | 0.147 | 0.928 |
| **Random effects** | **Variance** | **SD** | **N** |  |  |
| Individual | 0.015 | 0.125 | 86 |  |  |
| Study site | 0.002 | 0.049 | 4 |  |  |
| Year | < 0.001 | < 0.001 | 2 |  |  |

**Reference genotypes are AA for SNP163 exon 1 and SNP170 exon 3, and CC for SNP187 exon 1, SNP36 exon 6, SNP51 exon 9, SNP84 exon 9 and SNP66 exon 13.*

*^a^P-value from posthoc Tukey tests.*

**Table S26.** General generic GLMMs with a Poisson distribution for the relationship between exonic SNPs and number of nestlings fledged in males. (a) SNP226 exon 1, b) SNP163 exon 1, c) SNP187 exon 1, d) SNP170 exon 3, e) SNP36 exon 6, f) SNP51 exon 9, g) SNP84 exon 9, h) SNP66 exon 13.

| 1. **SNP226 exon 1** | | | | | |
| --- | --- | --- | --- | --- | --- |
| **Fixed effects** | ***β* ± SE** | ***Z*** | ***P*** | ***χ^2^_2_*** | ***P*** |
| Intercept | 1.945 ± 0.218 | 8.917 | < 0.001 |  |  |
| SNP226 AT | 0.051 ± 0.234 | 0.219 | 0.827 |  |  |
| SNP226 TT | 0.159 ± 0.229 | 0.694 | 0.488 | 1.219 | 0.543 |
| **Random effects** | **Variance** | **SD** | **N** |  |  |
| Individual | < 0.001 | < 0.001 | 42 |  |  |
| Study site | < 0.001 | < 0.001 | 3 |  |  |
| Year | < 0.001 | < 0.001 | 2 |  |  |
| 1. **SNP163 exon 1** | | | | | |
| **Fixed effects** | ***β* ± SE** | ***Z*** | ***P*** |  |  |
| Intercept | 2.069 ± 0.098 | 21.0 | <0.001 |  |  |
| SNP163 GG^a^ | -0.025 ± 0.116 | -0.214 | 0.830 |  |  |
| **Random effects** | **Variance** | **SD** | **N** |  |  |
| Individual | <0.001 | <0.001 | 41 |  |  |
| Study site | <0.001 | <0.001 | 3 |  |  |
| Year | <0.001 | <0.001 | 2 |  |  |
| 1. **SNP187 exon 1** | | | | | |
| **Fixed effects** | ***β* ± SE** | ***Z*** | ***P*** |  |  |
| Intercept | 2.071 ± 0.063 | 32.4 | < 0.001 |  |  |
| SNP187 CT^b^ | -0.060 ± 0.113 | -0.534 | 0.593 |  |  |
| **Random effects** | **Variance** | **SD** | **N** |  |  |
| Individual | < 0.001 | < 0.001 | 41 |  |  |
| Study site | < 0.001 | < 0.001 | 3 |  |  |
| Year | < 0.001 | < 0.001 | 2 |  |  |
| 1. **SNP170 exon 3** | | | | | |
| **Fixed effects** | ***β* ± SE** | ***Z*** | ***P*** | ***χ^2^_2_*** | ***P*** |
| Intercept | 2.009 ± 0.061 | 32.4 | < 0.001 |  |  |
| SNP170 CG | 0.199 ± 0.121 | 1.635 | 0.102 |  |  |
| SNP170 GG | -0.304 ± 0.307 | -0.989 | 0.323 | 4.069 | 0.130 |
| **Random effects** | **Variance** | **SD** | **N** |  |  |
| Individual | < 0.001 | < 0.001 | 42 |  |  |
| Study site | < 0.001 | < 0.001 | 3 |  |  |
| Year | < 0.001 | < 0.001 | 2 |  |  |
| 1. **SNP36 exon 6** | | | | | |
| **Fixed effects** | ***β* ± SE** | ***Z*** | ***P*** | ***χ^2^_2_*** | ***P*** |
| Intercept | 1.998 ± 0.092 | 21.7 | < 0.001 |  |  |
| SNP36 CT | 0.124 ± 0.124 | 1.000 | 0.317 |  |  |
| SNP36 TT | -0.002 ± 0.134 | -0.018 | 0.986 | 1.352 | 0.508 |
| **Random effects** | **Variance** | **SD** | **N** |  |  |
| Individual | < 0.001 | < 0.001 | 42 |  |  |
| Study site | < 0.001 | < 0.001 | 3 |  |  |
| Year | < 0.001 | < 0.001 | 2 |  |  |
| 1. **SNP51 exon 9** | | | | | |
| **Fixed effects** | ***β* ± SE** | ***Z*** | ***P*** | ***χ^2^_2_*** | ***P*** |
| Intercept | 2.065 ± 0.059 | 34.8 | < 0.001 |  |  |
| SNP51 CT | -0.091 ± 0.131 | -0.693 | 0.489 |  |  |
| SNP51 TT | -0.119 ± 0.382 | -0.312 | 0.755 | 0.559 | 0.755 |
| **Random effects** | **Variance** | **SD** | **N** |  |  |
| Individual | < 0.001 | < 0.001 | 42 |  |  |
| Study site | < 0.001 | < 0.001 | 3 |  |  |
| Year | < 0.001 | < 0.001 | 2 |  |  |
| 1. **SNP84 exon 9** | | | | | |
| **Fixed effects** | ***β* ± SE** | ***Z*** | ***P*** | ***χ^2^_2_*** | ***P*** |
| Intercept | 2.036 ± 0.147 | 13.8 | < 0.001 |  |  |
| SNP84 CT | 0.077 ± 0.165 | 0.468 | 0.640 |  |  |
| SNP84 TT | -0.069 ± 0.169 | -0.412 | 0.681 | 1.715 | 0.424 |
| **Random effects** | **Variance** | **SD** | **N** |  |  |
| Individual | < 0.001 | < 0.001 | 42 |  |  |
| Study site | < 0.001 | < 0.001 | 3 |  |  |
| Year | < 0.001 | < 0.001 | 2 |  |  |
| 1. **SNP66 exon 13** | | | | | |
| **Fixed effects** | ***β* ± SE** | ***Z*** | ***P*** | ***χ^2^_2_*** | ***P*** |
| Intercept | 1.981 ± 0.075 | 26.1 | < 0.001 |  |  |
| SNP66 CT | 0.139 ± 0.117 | 1.188 | 0.235 |  |  |
| SNP66 TT | 0.055 ± 0.221 | 0.252 | 0.801 | 1.399 | 0.496 |
| **Random effects** | **Variance** | **SD** | **N** |  |  |
| Individual | < 0.001 | < 0.001 | 37 |  |  |
| Study site | < 0.001 | < 0.001 | 3 |  |  |
| Year | < 0.001 | < 0.001 | 2 |  |  |

**Reference genotypes are AA for SNP163 exon 1 and SNP170 exon 3, and CC for SNP187 exon 1, SNP36 exon 6, SNP51 exon 9, SNP84 exon 9 and SNP66 exon 13.*

*^a^No male had genotype GA.*

*^b^No male had genotype TT.*
